# Self-Assembly-Directed Organization of a Fullerene–Bisporphyrin into Supramolecular Giant Donut Structures for Excited-State Charge Stabilization

**DOI:** 10.1021/jacs.1c05133

**Published:** 2021-07-14

**Authors:** Rubén Caballero, Myriam Barrejón, Jesús Cerdá, Juan Aragó, Sairaman Seetharaman, Pilar de la Cruz, Enrique Ortí, Francis D’Souza, Fernando Langa

**Affiliations:** † Instituto de Nanociencia, Nanotecnología y Materiales Moleculares (INAMOL), Universidad de Castilla-La Mancha, Campus de la Fábrica de Armas, 45071 Toledo, Spain; ‡ Neural Repair and Biomaterials Laboratory, Hospital Nacional de Parapléjicos (SESCAM), Finca la Peraleda s/n, 45071 Toledo, Spain; § Instituto de Ciencia Molecular, Universidad de Valencia, 46950 Paterna, Spain; ∥ Department of Chemistry, University of North Texas, 1155 Union Circle 305070, Denton, Texas 76203-5017, United States

## Abstract

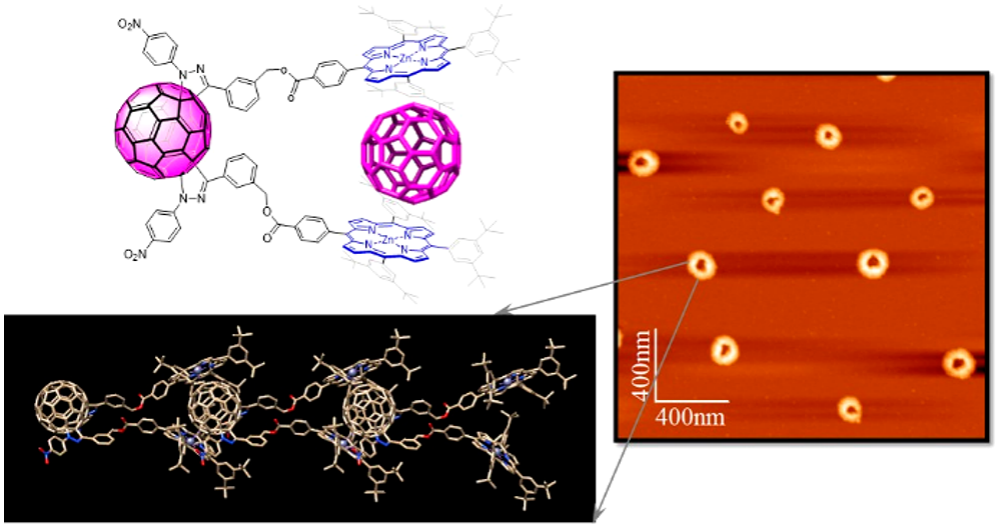

Functional materials composed of spontaneously self-assembled electron donor and acceptor entities capable of generating long-lived charge-separated states upon photoillumination are in great demand as they are key in building the next generation of light energy harvesting devices. However, creating such well-defined architectures is challenging due to the intricate molecular design, multistep synthesis, and issues associated in demonstrating long-lived electron transfer. In this study, we have accomplished these tasks and report the synthesis of a new fullerene–bis-Zn-porphyrin *e*-bisadduct by tether-directed functionalization of C_60_ via a multistep synthetic protocol. Supramolecular oligomers were subsequently formed involving the two porphyrin-bearing arms embracing a fullerene cage of the vicinal molecule as confirmed by MALDI-TOF spectrometry and variable temperature NMR. In addition, the initially formed worm-like oligomers are shown to evolve to generate donut-like aggregates by AFM monitoring that was also supported by theoretical calculations. The final supramolecular donuts revealed an inner cavity size estimated as 23 nm, close to that observed in photosynthetic antenna systems. Upon systematic spectral, computational, and electrochemical studies, an energy level diagram was established to visualize the thermodynamic feasibility of electron transfer in these donor–acceptor constructs. Subsequently, transient pump–probe spectral studies covering the wide femtosecond-to-millisecond time scale were performed to confirm the formation of long-lived charge-separated states. The lifetime of the final charge-separated state was about 40 μs, thus highlighting the significance of the current approach of building giant self-organized donor–acceptor assemblies for light energy harvesting applications.

## Introduction

Formation of self-assembled supramolecular systems involves spontaneous organization of one or more individual molecular components into an ordered architecture^1−3^ and has been Nature’s way of building molecular machinery to perform complex tasks necessary to sustain life on Earth.^4−7^ From a practical standpoint, supramolecular self-assembly offers an elegant solution to explore the limits placed between the top-down miniaturization approach and the bottom-up covalent building of large structures. The use of relatively smaller subunits (building blocks) in the construction of self-assembled supramolecular aggregates is the main advantage, as the synthesis of building blocks could be handled relatively easily and tuned to seek the desired physicochemical properties.^1−3^

Apart from the synthetic aspects of designing molecules that self-assemble spontaneously into ordered structures, the issue of achieving a functional response from the supramolecular architectures, either entirely new ones or a response better than that of the individual constituents, is of great interest to build the next generation of functional materials and devices. Consequently, research into the ordered assembly of functional molecules is very active, with potential applications in photonic and electronic devices, for example, light energy harvesting, light-emitting diodes, light modulators, sensors, and field effect transistors, to name a few.^8−20^ To provoke a collective response in self-assembled systems, the idea of molecular communication has been introduced to quantify electronic and/or geometric interactions between the molecular subunits. Individual organic molecules exhibit relatively sharp electronic transitions due to localized electron density distribution with minimal intermolecular electronic overlap integrals. Disadvantageously, this results in the reduction of charge carrier mobility, exciton delocalization, and electron/hole mobility. To circumvent this issue, intermolecular interactions can be used to direct how molecules self-assemble and interact to enhance electronic interactions.^20^

The great advantage of self-assembled supramolecular structures is the possible integration of functionalities using noncovalent interactions.^20^ This is particularly important for systems designed for light energy harvesting, where a combination of donor and acceptor molecules with at least one of them being photoactive, held in defined geometry and orientation, is essential.^8−24^ Over the years our teams have successfully demonstrated a range of novel self-assembled donor–acceptor systems and have confirmed the occurrence of photoinduced energy and electron transfer events leading to charge-separated states of appreciable lifetimes.^21−24^ In the majority of the reported studies, porphyrins, phthalocyanines, corroles, BF_2_-chelated dipyrromethene (BODIPY), and its azaBODIPY analogue formed the primarily photosensitizer/electron donor, while nanocarbons, viz. fullerenes (C_60_ and C_70_), endohedral fullerenes (Sc_3_N@C_60_ and Li^+^@C_60_), diameter-sorted single-wall carbon nanotubes (SWCNT(6,5) and SWCNT(7,6)), and exfoliated few-layer graphenes were used as electron acceptors. These studies improved our fundamental understanding of the self-assembly processes while enriching the supramolecular chemistry of carbon nanomaterial–photosensitizer derived donor–acceptor hybrids. Importantly, the kinetics of photoinduced events as a function of molecular structure and topology and the nature of the self-assembly protocol used were derived.^21−24^

**Chart 1:**
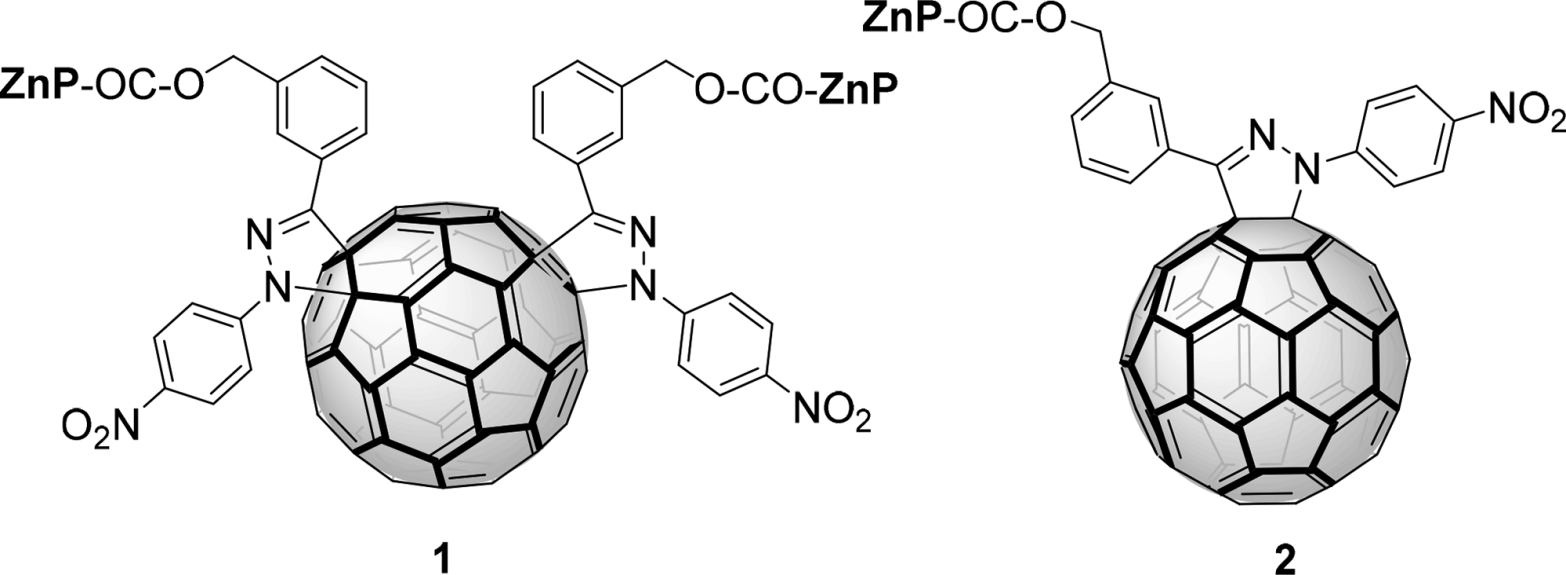


In this study, we hypothesize that by introducing electronic communication among the entities of self-assembled donor–acceptor supramolecular architectures, (D–A)_*n*_ (D–A are repeating units), we can improve migration of electron and/or hole located on the initially formed D^•+^–A^•–^ radical ion pair upon photoexcitation. This process would distantly separate the radical cations and radical anions along the supramolecular structure and subsequently slow the charge recombination process. As a consequence, the much-desired long-lived charge-separated states useful for designing a new generation of photocatalysts, especially for solar fuel production, could be produced efficiently. With this in mind, we have newly synthesized, by tether-directed functionalization, a [60]fullerene *e*-bisadduct (**1**) carrying two Zn porphyrins (Chart 1) and demonstrate supramolecular organization and photophysical events. Remarkably, the supramolecular assembly of the present bisporphyrin–C_60_ forms donut-shaped aggregates, primarily via π–π type charge transfer interactions, as demonstrated by means of optical absorption and emission, variable temperature ^1^H NMR, and AFM and supported by theoretical calculations. More importantly, upon photoexcitation, the supramolecular assembly generates long-lived charge-separated states of ∼1–40 μs lifetime due to electron/hole delocalization within the supramolecular structure. As a control system, monoadduct **2** was prepared and studied, showing significant differences in morphological and photophysical behavior with respect to **1**. In this case, neither donut-shaped aggregates nor long-lived charge-separated aggregates could be observed. Both facts demonstrate the need of both porphyrins for the supramolecular organization. The unprecedented results of this study demonstrate the success of supramolecular organization of donor–acceptor pairs in biomimetic light energy harvesting.

## Results and Discussion

### Synthesis and Characterization

**Scheme 1:**
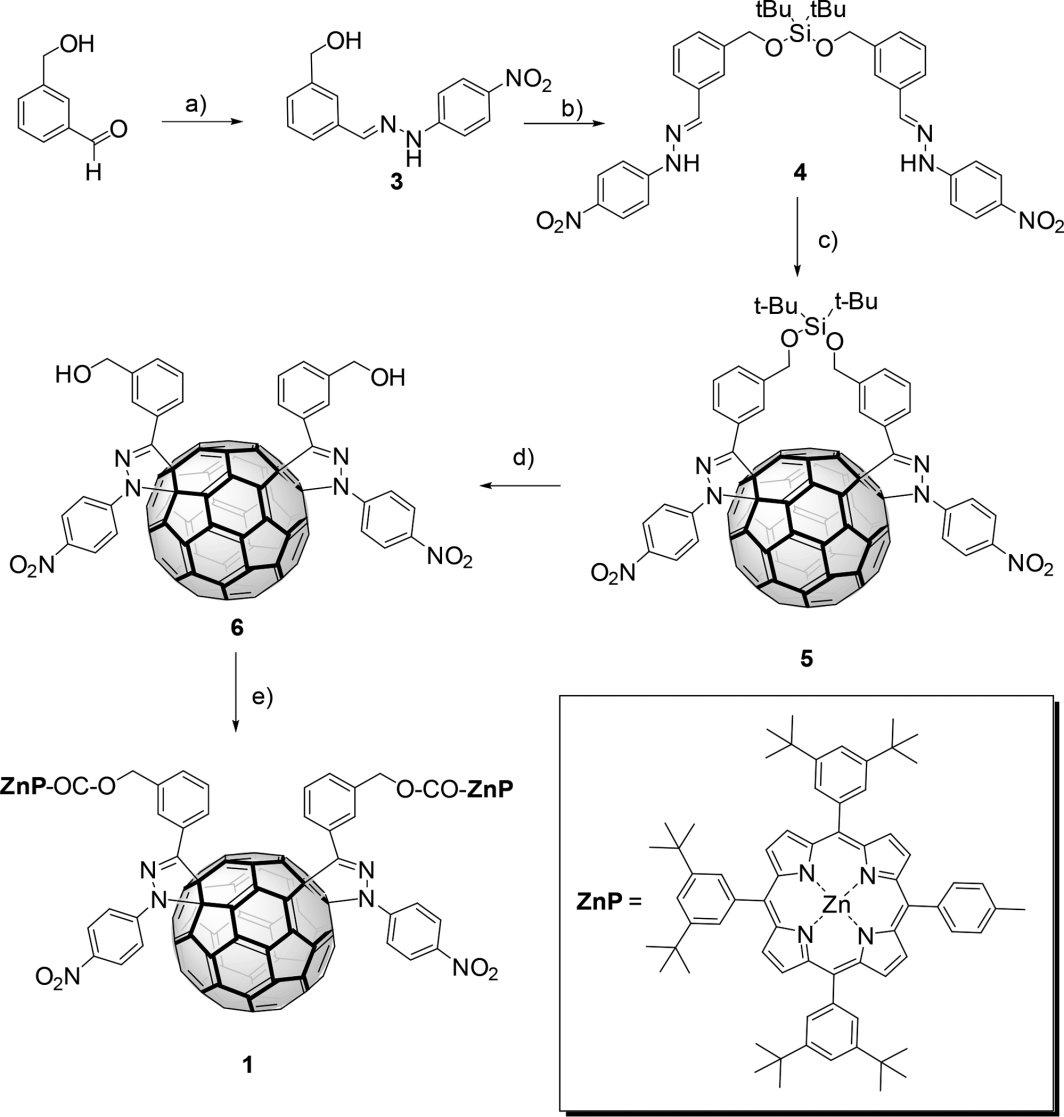
Synthetic Route to the Preparation of **1** Reagents and conditions: (a) *p*-nitrophenylhydrazine, acetic acid, EtOH, reflux, 10 min; (b) di-*tert*-butylsilylbis(trifluoromethanesulfonate), pyridine, CH_2_Cl_2_, 0 °C to rt, 12 h; (c) (i) NBS, CHCl_3_, 30 min, (ii) C_60_, (Et)_3_N, toluene, 12 h, 40 °C; (d) BF_3_·OEt_2_, CH_2_Cl_2_:CH_3_CN 2:1, rt, 36 h; (e) EDC, DMAP, ZnP-COOH (**7**), CH_2_Cl_2_, 0 °C to rt.

The synthetic route to bisadduct **1** is sketched in Scheme 1. Hydrazone **3** was prepared in 91% yield from *p*-nitrophenylhydrazine and 3-hydroxymethylbenzaldehide.^25^ The reaction of **3** with di-*tert*-butylsilylbis(trifluoromethanesulfonate) afforded **4** in 50% yield. Bisadduct **5** was prepared by the 1,3-dipolar cycloaddition of a bisnitrilimine, resulting from **4** and *N*-bromosuccinimide (NBS) in the presence of triethylamine and C_60_ by using the procedure previously described.^26^ Purification of the crude by column chromatography (silica gel, CS_2_:toluene from 1:1 to 0:1) afforded in 35% yield a mixture of isomers of **5** as determined by ^1^H NMR and HPLC (Figure S1), where two peaks with the ratio 2.2:1 were observed. Preparative HPLC allowed the separation of the two fractions. The major one (retention time = 9.2 min) corresponds to a single isomer (named **5a**) as inferred from the analysis of the ^1^H NMR signals associated with the *p*-nitrophenyl moieties (Figure S9). In contrast, the second fraction (retention time = 10.8 min) reveals, by ^1^H NMR (Figure S10), to be a mixture of two isomers (named **5b** and **5c**) in an approximately 3:1 ratio. The ^1^H NMR of **5a** (Figure S9) showed two inequivalent AA′XX′ systems for the *p*-nitrophenyl groups appearing between 8.0 and 8.4 ppm as four doublets; the aromatic protons of the spacer appear between 7.9 and 7.3 ppm, and the benzylic protons appear each one as a doublet with a geminal *J* = 13.4 Hz. The fact that the hydrogens of both *p*-nitrophenyl groups show different shifts evidences the lack of symmetry of the molecule.

**Figure 1:**
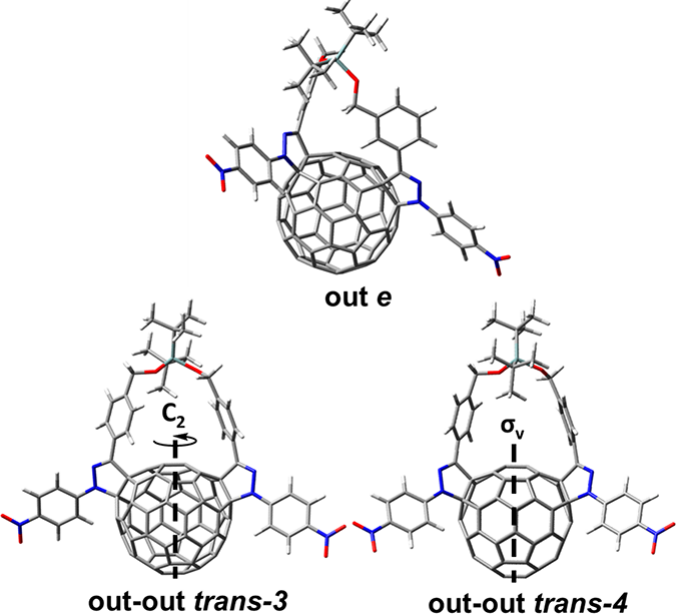
Calculated structures of the most stable regioisomers of compound **5**.

With the aim of obtaining additional information about the stability of the different regioisomers of **5**, theoretical calculations were performed by using the density functional density (DFT) B3LYP/6-31G method. As summarized in Table S1, the out *e*-isomer^27^ is the most stable followed by the out–out *trans*-3 (5.72 kcal mol^–1^) and out–out *trans*-**4** (6.55 kcal mol^–1^) isomers (Figure 1). Only the out *e*-isomer shows no molecular symmetry, which is consistent with the ^1^H NMR spectrum of the major isomer **5a** (*vide supra*), having two different *p*-nitrophenyl groups. Thus, **5a** is assigned to the out *e*-isomer structure. Tentatively, the minor isomers **5b** and **5c**, found in the second fraction, are associated with the out–out *trans*-**3** and out–out *trans*-**4** (see all the possible bisadduct isomers in Figure S33).

Rupture of the silyl ester group in out *e*-**5** with BF_3_·OEt_2_ yields after purification by column chromatography dialcohol out *e*-**6**, which was unambiguously characterized (see Figures S13–S17). The ^1^H NMR signal assignment of out *e*-**6** clearly indicates the presence of a single isomer (Figure S14). Interestingly, when the reaction is done with the mixture of isomers of **5** (*vide supra*), after purification of the crude by preparative thin layer chromatography, out *e*-**6** could be isolated as a pure isomer in 20% yield. This procedure allowed us to work with a higher amount of product.

Next, the pure out *e*-**6** isomer was reacted with ZnP-COOH (**7**)^28^ by using 1-ethyl-3-(3-(dimethylamino)propyl)carbodiimide hydrochloride (EDC) and 4-(dimethylamino)pyridine (DMAP) as activators (step “e” in Scheme 1), affording, after purification by column chromatography (SiO_2_, toluene) followed by gel permeation chromatography (Biobeads SX1, dichloromethane), **1** as a purple solid in 30% yield. The purity of **1** was checked by HPLC, and its structure was confirmed by several spectroscopic techniques such as ^1^H and ^13^C NMR, FT-IR, and MALDI-TOF mass spectrometry (see Figures S18–S22). Interestingly, in the ^1^H NMR spectrum of **1** (at 298 K) the two α-methylene groups, with respect to the ester moiety, appear as four doublets at 5.24, 5.15, 4.95, and 4.82 ppm, indicating that the four H atoms of both methylene groups have different environments (Figure S18). For the sake of comparison, monoadduct **2** (Chart 1) was prepared by a similar route (see the Supporting Information for further details). It is noteworthy that the above-mentioned methylene group in monoadduct **2** appears as a singlet in the ^1^H NMR spectrum (Figure S28).

Soft ionization mass spectrometric methods such as MALDI-TOF allow the analysis of the molar mass for large aggregates and polymers.^29^ Using this technique, we found the molecular ion peak of **1**, in MALDI-TOF MS (matrix: DCTB), at *m*/*z* = 3340.2 with well-resolved isotopic distribution. Interestingly, aggregates of up to 13 units of **1** were observed in the MALDI-TOF experiments (Figure S23), suggesting the formation of supramolecular oligomers.

**Figure 2:**
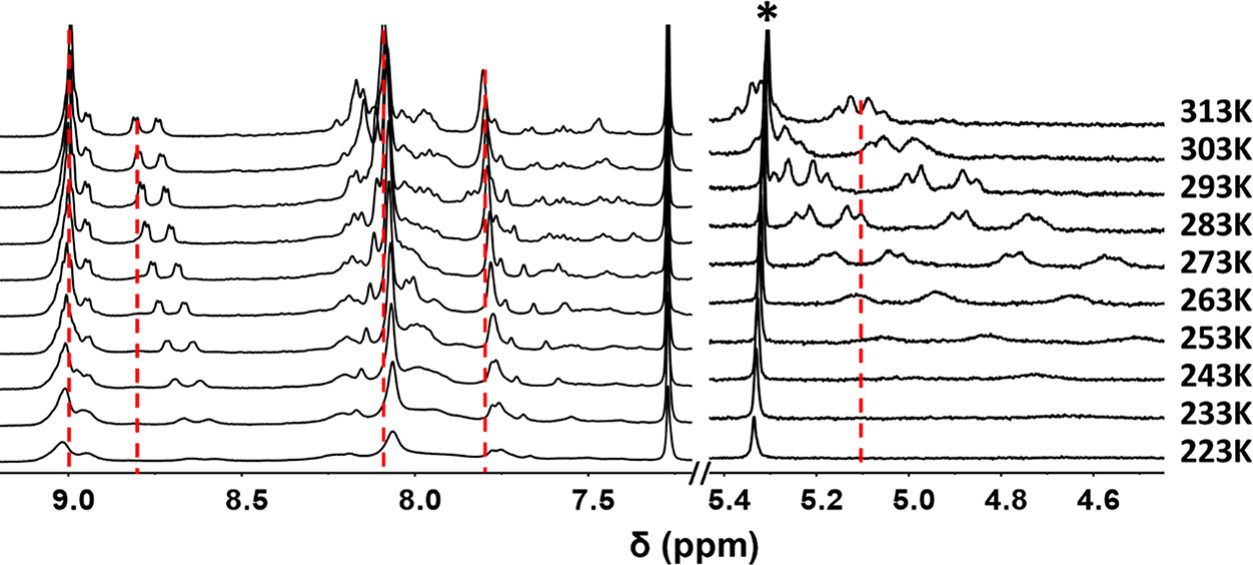
Parts of the ^1^H NMR spectra recorded for **1** at different temperatures (1.5 mM CDCl_3_, 400 MHz) (∗ denotes residual dichloromethane).

The intermolecular aggregation of **1** was further investigated by ^1^H NMR (1.5 mM CDCl_3_, 400 MHz) in the range from 313 to 223 K (Figure 2). In the spectra, significant changes are observed for all the signals upon decreasing the temperature, pointing out to the existence of high molecular weight aggregates in solution. The signal at 9.00 ppm at room temperature, attributed to the β-pyrrolic hydrogens in 12, 13, 17, and 18 positions of the porphyrins (those away from the linker to the fullerene), suffers a small deshielding (Δδ = −0.02 ppm) as temperature decreases. In contrast, those at 8.94 and 8.78 ppm, attributed to hydrogens in 2, 3, 7, and 8 positions, closer to the ester group, are shielded up by 0.22 ppm at 223 K. The characteristic aromatic signals of the 3,4-di-*tert-*butylphenyl groups appear at 8.08 and 7.86 ppm, the latter being assigned to the six H atoms in *para* positions and are differently affected by the temperature decrease with upfield shifts of 0.1–0.2 ppm. The signals associated with the methylene hydrogens between 4.5 and 5.5 ppm are shifted upfield up to 1.26 ppm, being the most significant change in the spectra upon cooling. Furthermore, a noticeable change is observed for the aliphatic signals corresponding to the *tert-*butyl hydrogens, which initially appear as a broad signal at 1.52 ppm, and some of them shield up upon temperature decrease (Figure S19). This indicates that the formation of the supramolecular aggregates does not affect equally to all the *tert*-butyl groups of **1** as theoretical calculations suggest (*vide infra*, see Figure 5).

To gain more insight into these spectral changes, a variable temperature ^1^H NMR study was also performed for monoadduct **2**, which only bears one porphyrin “arm” and where the formation of the supramolecular complex is not detected. As mentioned above, compared to **1**, the most significant difference is observed for the methylene H atoms which appear as a singlet (Figure S32), in contrast to the two doublet signals recorded for **1** (Figure 2). Moreover, the temperature-dependent NMR studies revealed that the singlet of the methylene group in **2** shields up by only 0.18 ppm when the spectra are registered at 223 K. The shift recorded for the rest of the H atoms differ by less than ±0.01 ppm (Figure S32).

### AFM Study of Intermolecular Aggregates

**Figure 3:**
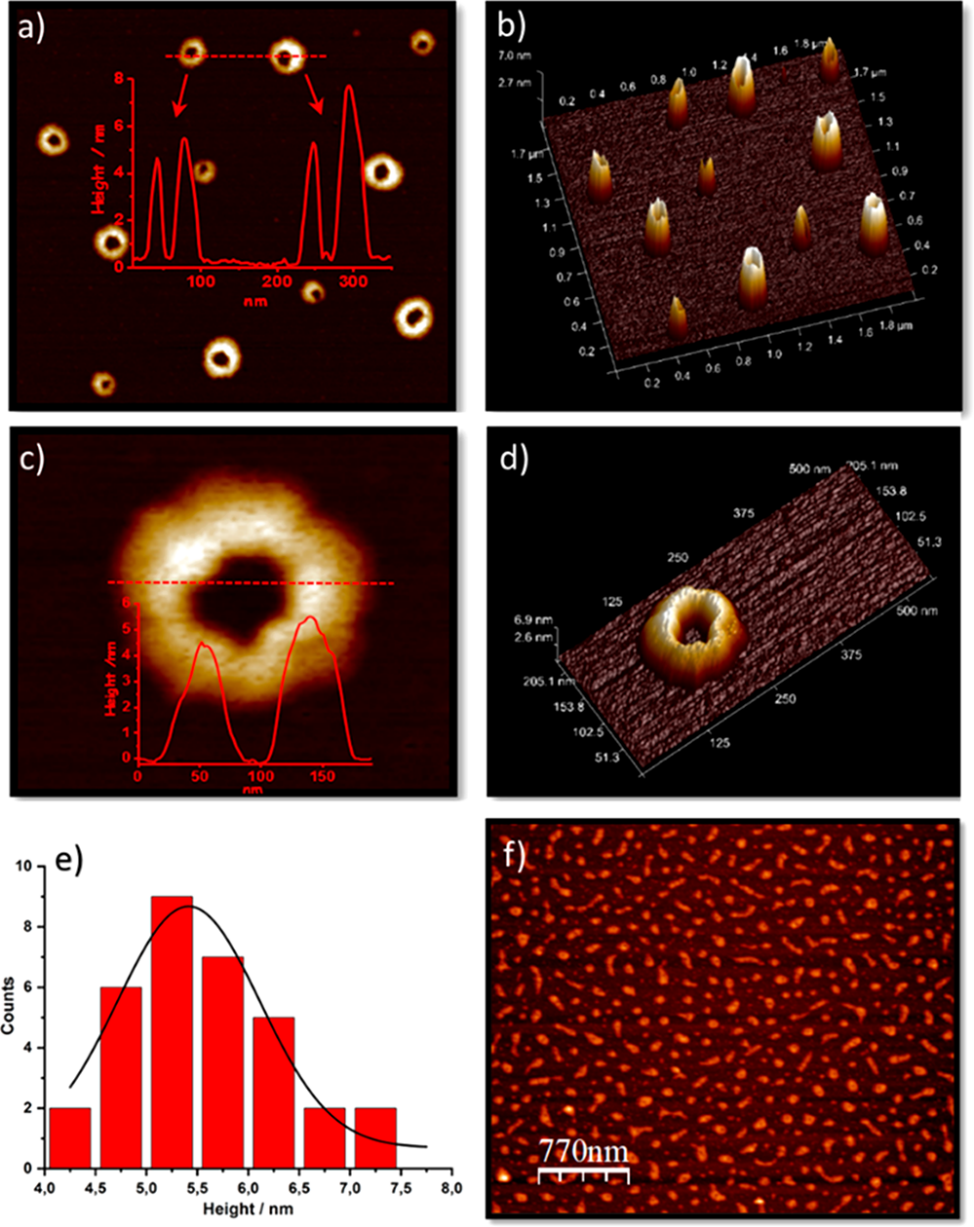
(a) AFM topographic images of the donut-shaped structures formed by **1** along with the representative height profile in red corresponding to the red dotted line. (b) 3D height image of (a). (c) Enlarged image of one donut-shaped structure along with its corresponding height profile in red. (d) 3D height image of (c). (e) Height distribution histogram resulting from the study of 35 donut-like different structures. (f) AFM topographic image showing fiber-type structures from a freshly prepared solution of **1**.

Atomic force microscopy (AFM) was expected to provide additional information about the supramolecular organization of the assemblies resulting from the aggregation of fullerene–bisporphyrin bisadduct **1**. AFM can be used to image the molecular structure of the assemblies in solution even though the drying process can inevitably cause some changes.^30^ To this end, dilute solutions of **1** in CH_3_Cl were spin-coated onto mica surfaces, and AFM studies were accomplished in tapping mode. The studies revealed the presence of spherical-shaped structures with a hollow core (donut-like structures) of different sizes (Figure 3), suggesting the formation of oligomers with variable length. The line profiles along the red dotted lines shown in Figures 3a and 3c revealed the presence of structures with an average height of 5.4 ± 0.7 nm, based on the statistical analysis of 35 donut-like structures (see the histogram Figure 3e). If we suppose that the aggregated structures are lying flatwise on the mica surfaces, the observed height may correspond to the distance existing between both porphyrins. However, because of the aggregation effect that may occur during the deposition process, the observed height could also be the result of overlapped bisadduct aggregates. If we consider the MALDI-TOF results that suggest the formation of aggregates consisting of up to 13 units of **1**, the donut-like structures could be attributed to aggregates that result from the roll-up of initial fiber-like structures formed in solution. To prove this, a 24 h AFM study was conducted where the time evolution of the aggregation process was assessed. Thus, a solution of **1** in CH_3_Cl was prepared and spin-coated immediately onto mica surfaces. As observed in Figure 3f and Figure S34a, the presence of fiber-like aggregates, consisting probably of assemblies of several bisadduct units, is dominant in freshly prepared solutions. The same solution was again spin-coated onto mica surfaces after 2 h; at this stage, the fiber-type structures started to show some curvature, and the donut-like aggregates seemed to be more evident by AFM (Figure S34b). A final study after 24 h revealed the presence of the above-described donut-like structures (Figure S34c), confirming the evolution of the first-formed fiber-like structures to the final donut-like aggregates, probably through head-to-tail interactions.

In an attempt to confirm the necessity of two porphyrins in the formation of donut-like aggregates, AFM images were also taken for monoadduct **2** (Figure S35). As observed, under identical conditions, the AFM images revealed that the monoadduct is randomly arranged with heights that vary from 8 to 12 nm. These results confirmed that the aggregation into donuts is only possible when two porphyrin units are present.

### Computational Study of Intermolecular Aggregates

**Figure 4:**
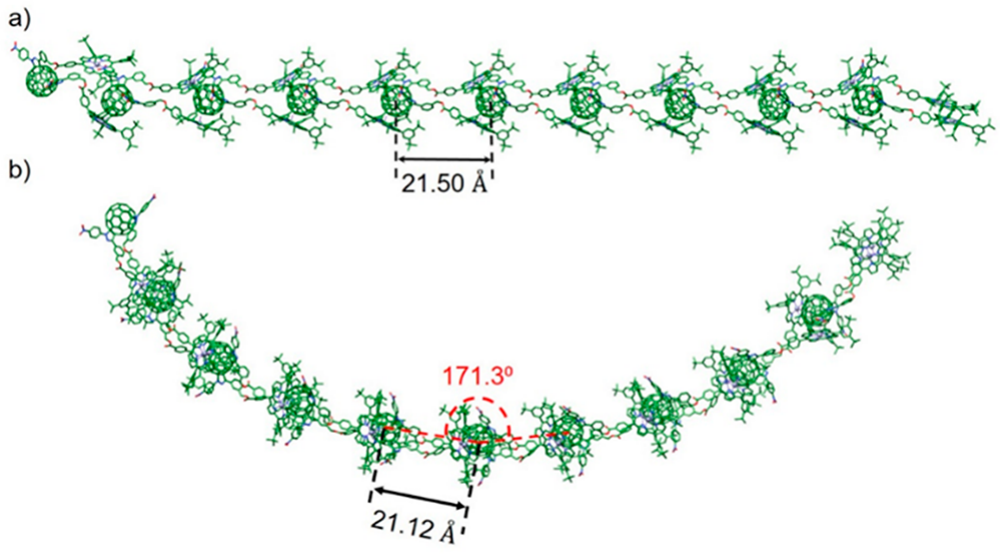
GFN-FF-optimized structures calculated for a linear (a) and curved (b) decamer aggregate of bisadduct **1**.

To get a deeper insight into the structure of the aggregates formed by bisadduct **1**, the self-assembly of this system was theoretically investigated at the molecular mechanics (MM) level by using the generic GFN-FF force field recently developed by Spicher and Grimme.^31^ GFN-FF is claimed to outperform other force fields in terms of generality and accuracy, approaching the performance of much more elaborate quantum-mechanical methods. Supramolecular (**1**)_*n*_ oligomers (*n* = 2, 3, 4, 5, 6, 8, 10, 12, 14, 16, and 18) with linear and curved structures were modeled at the GFN-FF level as tentative structures to the fibers and donuts experimentally observed. Figure 4 displays the fully optimized structures for both types of aggregates of the supramolecular decamer (*n* = 10) as a representative example (Figure S36 shows the optimized structures of shorter and longer oligomers). Curved structures are computed to be more stable than linear structures by 2.6 kcal mol^–1^ per monomeric unit. This energy difference remains constant from the hexamer and indicate that linear (fiber) and curved (donut) structures may be competitive. In both structures, bisadduct **1** exhibits a conformation with both porphyrin-bearing arms extended and wrapping the fullerene cage of a vicinal molecule. In linear aggregates, monomers are separated by 21.50 Å, which corresponds to the distance between the center of mass of adjacent fullerenes. In curved aggregates, fullerenes are separated by a slightly shorter distance of 21.12 Å and form angles of ∼171°. This angle defines the curvature of the aggregate and by adding more monomers would form a round donut-type structure with an estimated diameter of 23 nm. This value is smaller than that observed from AFM experiments, but it should be stressed that the theoretical structure is calculated in gas phase for ideal 1D chains without taking into account more complex agglomerated structures or the interactions with the surface. Calculations performed for the supramolecular (**1**)_9_ oligomer, as an intermediate oligomer, at the more accurate semiempirical GFN2-xTB level^32^ confirm the curved aggregate to be slightly more stable than the linear aggregate (see the Supporting Information and Figure S37 for more details). The small energy difference between linear and curved structures supports the evolution from fiber-type aggregates initially observed by AFM upon deposition from freshly prepared solutions to the donut-type aggregates evidenced after 24 h deposition from solution (Figure S34).

**Figure 5:**
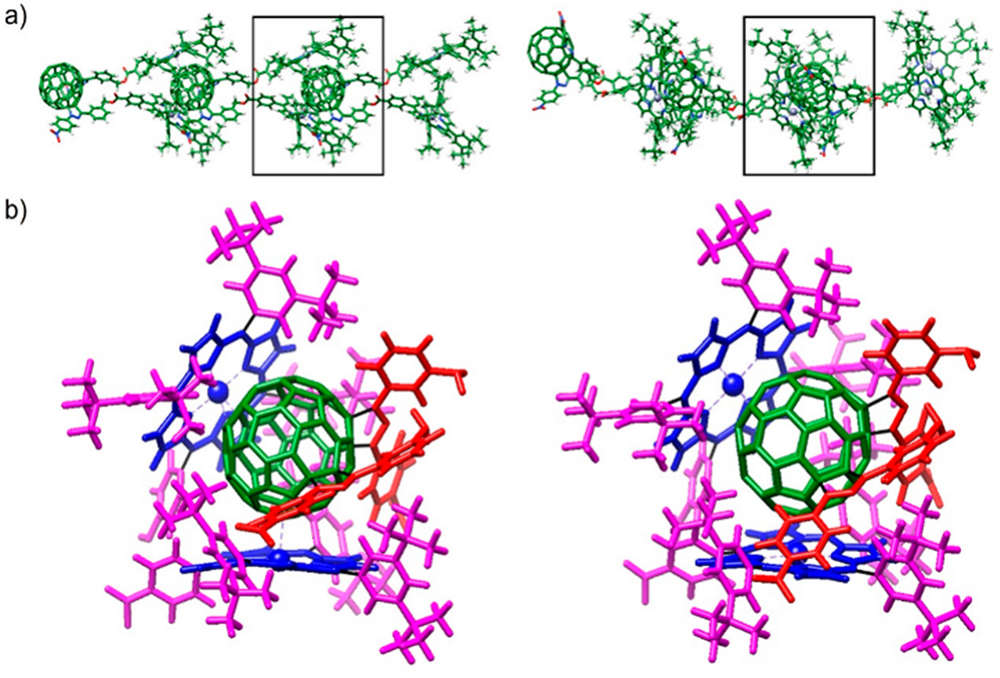
(a) GFN-FF-optimized structure of the central trimer of the linear (left) and curved (right) decamer aggregate of bisadduct **1**. (b) Zoomed-in view of the interaction center (square in (a)) for linear (left) and curved (right) aggregates. C_60_ fragment in green, C_60_ substituents in red, porphyrin ring in blue, and porphyrin di-*tert*-butylphenyl meso substituents in magenta.

To perform a more detailed analysis of the noncovalent interactions taking place in the (**1**)_*n*_ aggregates, Figure 5a shows the central trimers extracted from the fully optimized linear and curved decamers of Figure 4. Figure 5b displays a magnified view of the interaction center between two vicinal monomers in the aggregate, in which molecular fragments are drawn in different colors to make easy the identification of the interactions. The two benzyl benzoate moieties of the monomer extend in the same direction interacting between them through stabilizing C–H···π and C–H···O contacts. These interactions mainly involve the CH_2_ groups, which exhibit different chemical environments as suggested by ^1^H NMR experiments and facilitate the wrapping of a vicinal fullerene unit by the two porphyrins. The porphyrin moieties act as “hands” that catch the fullerene ball (in green) through π··· π interactions with the porphyrin fragment (in blue) and also C–H···π interactions with the *tert*-butyl pendant groups (in magenta). For both linear and curved aggregates, the Zn atoms are centered over C_60_ 5–6 bonds with short Zn–C distances in the 2.49–2.56 Å range. The nearest distance between *tert*-butyl hydrogens and C_60_ carbons is 2.55 Å. The distance between the two Zn atoms is on average 9.67 Å for the linear aggregate and slightly shorter (9.44 Å) for the curved aggregate. The distance between the most external *tert*-butyl hydrogens is of 24.23 Å for the linear disposition and increases to 25.76 Å for the curved one. These values are a half of the height measured by AFM for the structures deposited on mica, suggesting that more agglomerated structures can be formed. There is an important difference between the two porphyrin moieties. For that placed on the top of the fullerene cage in Figure 5b, the three di-*tert*-butylphenyl meso substituents are interacting with the ball. In contrast, the three di-*tert*-butylphenyl groups of the bottom porphyrin are interacting both with the π-surface of C_60_ and the C_60_ substituents. In particular, short C–H···O contacts between *tert-*butyl hydrogen atoms and nitro groups are visualized. These contacts are significantly shorter for curved aggregates (2.13–2.28 Å) than for linear aggregates (2.14–2.46 Å) and contribute to stabilize the former.

### Spectral and Electrochemical Studies

**Figure 6:**
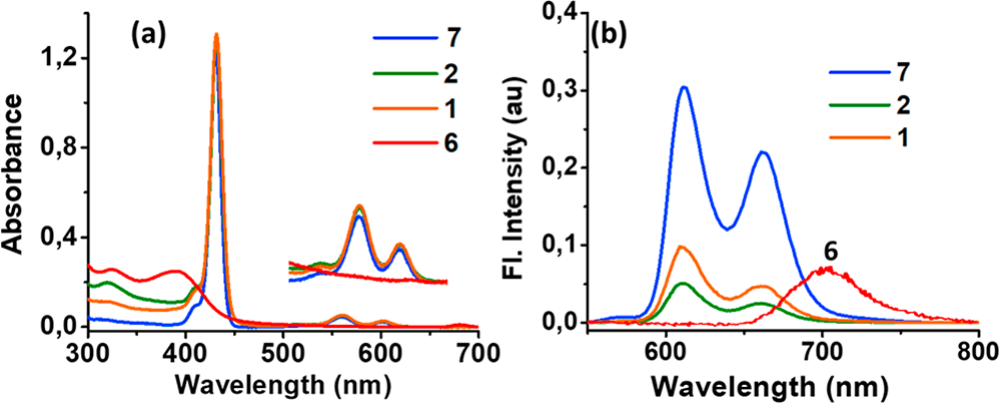
(a) Absorption (normalized to the Soret band) and (b) fluorescence spectra of the indicated compounds in benzonitrile. The compounds were excited at 432 nm corresponding to the Soret band. The inset in (a) shows the expanded visible spectral region. The fluorescence of control fullerene **6** is also shown in (b) for comparison (λ_ex_ = 330 nm, not drawn to concentration scale).

Figure 6a shows the absorption spectrum (normalized to the Soret porphyrin band) of compounds **1**, **2**, and **7**, along with that of the doubly functionalized compound **6** (Scheme 1), in benzonitrile. For **1**, the Soret band at 432 nm and the visible Q bands at 562 and 602 nm are slightly red-shifted by 1–2 nm compared to **7** and monoadduct **2**. Decreasing by half or increasing by a factor of 2 the concentration of **1** had virtually no spectral broadening or shift effect. This suggests that the supramolecular association described above is not perturbing the electronic structure of ZnP considerably. The fluorescence spectra shown in Figure 6b reveal two emission peaks of ZnP at 612 and 662 nm. As expected, substantial quenching is observed for both compound **1** (68%) and **2** (84%). In a control experiment, the fluorescence spectrum of compound **6** was also recorded, and a broad emission with a peak maximum at 704 nm was observed. Excitation of ZnP at either the Soret or visible bands of compounds **1** and **2** revealed no traces of emission of **6**, indicating that energy transfer from ZnP to C_60_ is not a likely mechanism of fluorescence quenching. It is also to be pointed out that at the Soret excitation of ZnP at 432 nm about 6% of absorbance is due to C_60_. It is likely that any weak emission of C_60_ is buried under the strong emission peaks of ZnP in the spectral range.

**Figure 7:**
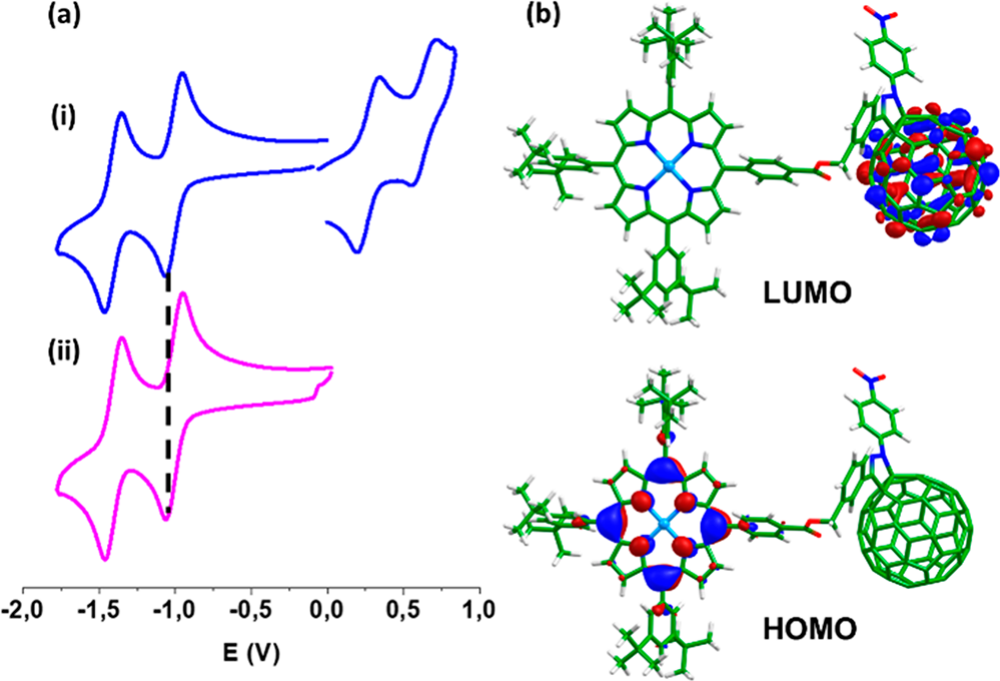
(a) Cyclic voltammograms of **1** (i) and C_60_ (ii) in *o*-dichlorobenzene:acetonitrile (4:1 v/v) containing 0.1 M (Bu_4_N)ClO_4_; scan rate = 100 mV s^–1^. (b) Isovalue contour plots (±0.03 au) calculated at the B3LYP-6-31G** level for the HOMO and LUMO of monoadduct **2**.

To probe the effect of the different number of pyrazolino rings and nitrophenyl substituents on the reduction potentials of C_60_, cyclic voltammetry (CV) and Osteryoung square-wave voltammetry (OSWV) studies were performed, as any change in the reduction potentials would perturb the energetics of the envisioned photodriven electron-transfer reactions. Figure 7a shows representative CVs of compounds **1** and C_60_ in *o*-dichlorobenzene:acetonitrile (4:1 v/v), and the measured redox potentials are summarized in Table S4 along with additional CVs and OSWVs. Both compounds **1** and **2** exhibit two reduction and two oxidation processes within the accessible potential window, displaying slightly better electron affinities (*E*^1^_red_ = −1.01 V for **1** and −1.00 V for **2**) than pristine C_60_ (−1.03 V) as described for other pyrazolinofullerene derivatives.^26^ In the anodic side of the voltammograms, the first oxidation process is associated with the porphyrin moieties and appears at +0.29 and +0.30 V for **1** and **2**, respectively. It may be mentioned here that for fulleropyrrolidine derivatives synthesized according to Prato’s method reduction potentials of C_60_ depend on the degree of functionalization.^33^ In general, reduction of C_60_ becomes harder by about 100 mV upon addition of one pyrrolidine ring on C_60_ due to conversion of two sp^2^ carbons to sp^3^ carbons. This trend is confirmed when saturating more sp^2^–sp^2^ carbon–carbon bonds, and for example, C_60_ becomes harder to reduce by about 200 mV upon addition of two pyrrolidine rings having different regioisomers.^33b^ In contrast, the bis- and monopyrazole functionalization in compounds **1** and **2** have made the C_60_ cage a slightly better electron acceptor. That is, the electron-acceptor ability of C_60_ is not compromised by the present synthetic strategy.

The electronic properties of compounds **1** and **2** were also theoretically investigated at the density functional theory (DFT) B3LYP/6-31G** level.^34^ The minimum-energy molecular structures calculated for **1** and **2** are drawn in Figure S38. As depicted in Figure 7b for monoadduct **2**, the HOMO is fully localized over the porphyrin moiety whereas the LUMO resides over the C_60_ fragment. Analogous HOMO and LUMO topologies are obtained for bisadduct **1** (Figure S39). The energy of the LUMO, calculated by using *o*-dichlorobenzene as solvent, was found to decrease in passing from pristine C_60_ (−3.10 eV), to monoadduct **2** (−3.22 eV), and to bisadduct **1** (−3.26 eV). This trend agrees with the gain in the electron-acceptor ability inferred from electrochemical data as pyrazoline units are attached to the C_60_ ball. The electron affinity, computed as the energy difference between the neutral molecule and the anion at the equilibrium geometry of the neutral system, actually increases in passing from C_60_ (3.08 eV), to monoadduct **2** (3.21 eV), to bisadduct **1** (3.25 eV). Figure S40 presents the spin densities calculated for the cation and anion radical species of **1** and **2** and shows that upon oxidation the electron is extracted from the porphyrin moiety, whereas the extra electron introduced upon reduction is fully injected in C_60_.

**Figure 8:**
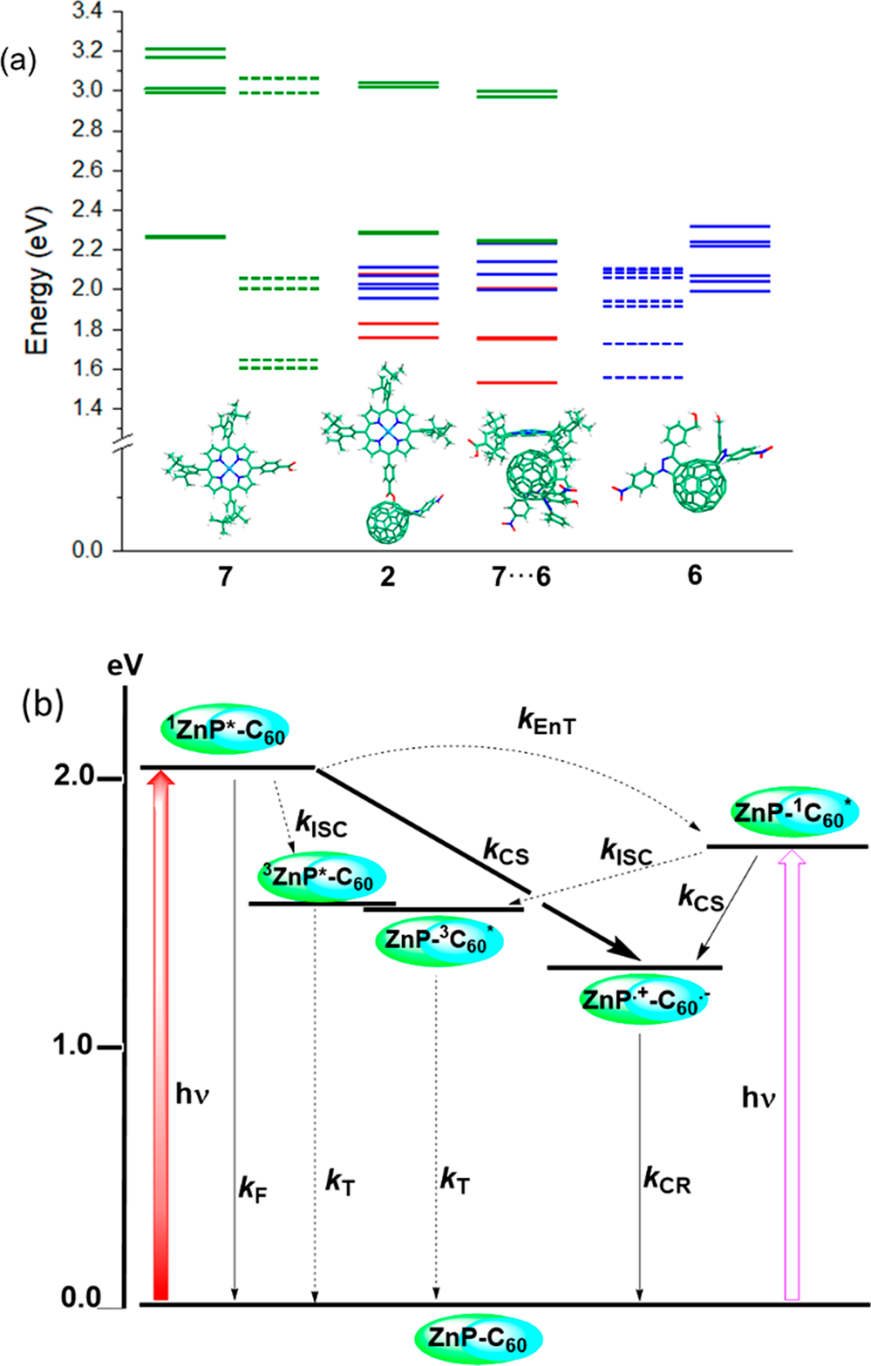
(a) Energy level diagram showing the vertical excitation energies calculated for the singlets (solid lines) and triplets (dashed lines) of ZnP–COOH (**7**) and **6** and for the singlets of monoadduct **2** and the **7···6** supramolecular dimer present in aggregates of bisadduct **1**. Excited states localized on the ZnP environment are colored in green, those on C_60_ in blue, and ZnP^·+^–C_60_^·–^ charge-transfer states in red. (b) Energy level diagram showing different photochemical events upon photoexcitation of ZnP in compounds **1** and **2**. Thick arrow: most likely process; thin and dashed arrow: less likely process (EnT = energy transfer, CS = charge separation, CR = charge recombination, ISC = intersystem crossing, T = triplet emission, and F = fluorescence emission).

Figure 8a shows the lowest-energy excited states computed for the different molecular fragments of compounds **1** and **2** by using the time-dependent DFT (TDDFT) B3LYP/6-31G** approach and benzonitrile as solvent. The lowest S_1_ and S_2_ singlet states of **7** are computed almost degenerate at 2.26 eV (549 nm), in good agreement with the Q-band observed at 2.21 eV (562 nm) and have low intensities with small oscillator strengths (*f*) of 0.079 and 0.041, respectively. The following two singlet states, S_3_ and S_4_, are calculated close in energy at 2.99 eV (414) and 3.01 eV (411 nm), respectively, with high intensity (*f* = 1.475 and 1.612, respectively), and give rise to the Soret band experimentally observed at 2.87 eV (432 nm). The lowest triplet states of **7** are calculated at 1.61 and 1.64 eV (T_1_ and T_2_) and 2.01 and 2.04 eV (T_3_ and T_4_). In contrast to porphyrin, which presents a few low-energy excited states, the C_60_ derivative **6** has a manifold of states, the lowest two singlets being found at 1.99 and 2.04 eV and the lowest triplets at 1.55 and 1.73 eV. For monoadduct **2**, the lowest-lying singlet excited states correspond to intramolecular ZnP^·+^–C_60_^·–^ charge-transfer states in which one electron is transferred from the porphyrin moiety to the C_60_ unit. These states are calculated in the 1.75–2.10 eV range, have zero oscillator strengths, and imply excitations from the HOMO and HOMO–1 located on the ZnP environment to the LUMO, LUMO+1, and LUMO+2 spreading over the C_60_ cage (Table S2). For instance, in the S_1_ state resulting from the HOMO → LUMO excitation, a total charge of 0.99*e* is transferred from the ZnP environment to the C_60_ unit. The states associated with the Q and Soret bands of ZnP are computed at 2.28 and 3.02 eV, respectively, close to the energies found for **7**. A practically identical distribution of localized and charge-transfer states is computed for bisadduct **1**, the main difference being the doubling of the states concerning the ZnP moiety. To estimate the energy position of the intermolecular ZnP^·+^···C_60_^·–^ charge-transfer states that are expected to appear for the aggregate of bisadduct **1**, we have used a supramolecular **7**···**6** dimer extracted from the (**1**)_10_ aggregate and reoptimized at the B3LYP/6-31G** level including the D3 dispersion term.^35^ TDDFT calculations predict intermolecular ZnP^·+^···C_60_^·–^ states in the 1.57–1.88 eV range with small oscillator strengths (*f* = 0.001–0.006) (Table S3), which are located significantly lower in energy than the intramolecular ZnP^·+^–C_60_^·–^ states (Figure 8a). The stabilization of the intermolecular ZnP^·+^···C_60_^·–^ states results from the fact that the donor ZnP moieties and the acceptor C_60_ unit are placed at significantly shorter distances in the aggregate. For instance, the distance between the Zn atom and the nearest carbon atom of the C_60_ unit is of 5.75 Å in **1** and decreases to only 2.78 Å for the aggregate due to the intermolecular interactions.

An energy level diagram depicting different photochemical events in **1** and **2** is shown in Figure 8b. The energy of the different states was calculated from spectral, electrochemical, and computational data, according to the Rehm–Weller approach.^36^ It is clear from the figure that the ^1^ZnP* singlet excited species formed upon photoexcitation can undergo deactivation competitively by at least four thermodynamically allowed processes, viz., fluorescence emission to produce the photosensitizer ground state, intersystem crossing to populate the photosensitizer triplet state ^3^ZnP*, singlet–singlet energy transfer to produce the acceptor singlet excited state ^1^C_60_*, and electron-transfer reaction involving fullerene to produce the ZnP^•**+**^–C_60_^•–^ charge-separated state. Because partial quenching was observed in both **1** and **2** (Figure 6b), part of the ZnP follows the path of fluorescence emission. Singlet–singlet energy transfer, although thermodynamically possible, is less likely as the earlier discussed steady-state fluorescence data did provide sufficient evidence for the occurrence of this process. It appears that electron transfer is likely the mechanism of majority of fluorescence quenching. Another key point is that the experimentally calculated energy of the charge-separated state is below that of ^3^ZnP* and ^3^C_60_*. Under such circumstances, the charge-separated state could charge-recombine directly to the ground state. To secure direct experimental evidence for these envisioned photoprocesses and associated rates, and to seek whether the earlier-discussed aggregation would improve lifetime of charge-separated states, femto- and nanosecond transient absorption studies were performed in benzonitrile, and the results are summarized below. Prior to this, to help interpret the transient–absorption data, **7** was chemically oxidized by using nitrosonium tetrafluoroborate (NOBF_4_), as shown in Figure S45. The oxidized product revealed new peaks at 615, 782, and 870 nm corresponding to the formation of the ZnP^•**+**^ radical cation. The radical anion of C_60_, C_60_^•–^, is known to have an absorption band in the near-IR region of 1020 nm. The absorption spectra of the ZnP–COOH^•**+**^ radical cation and **6**^•–^ radical anion species were also calculated at the B3LYP/6-31G** level after reoptimizing the geometries of the neutral systems. For ZnP–COOH^•+^, absorptions are predicted at 1.48 eV (840 nm, *f* = 0.078), 1.66 eV (748 nm, *f* = 0.057), 1.88 eV (659 nm, *f* = 0.015), and 1.99 eV (623 nm, *f* = 0.006), in rather good correlation with the experimental findings. For **6**^•–^, a low-intensity absorption is predicted at 1.21 eV (1024 nm, *f* = 0.040), slightly shifted with respect to that calculated for the C_60_^•–^ anion (1052 nm, *f* = 0.075) at the same theoretical level.

### Femto- and Nanosecond Transient Absorption Spectral Studies

**Figure 9:**
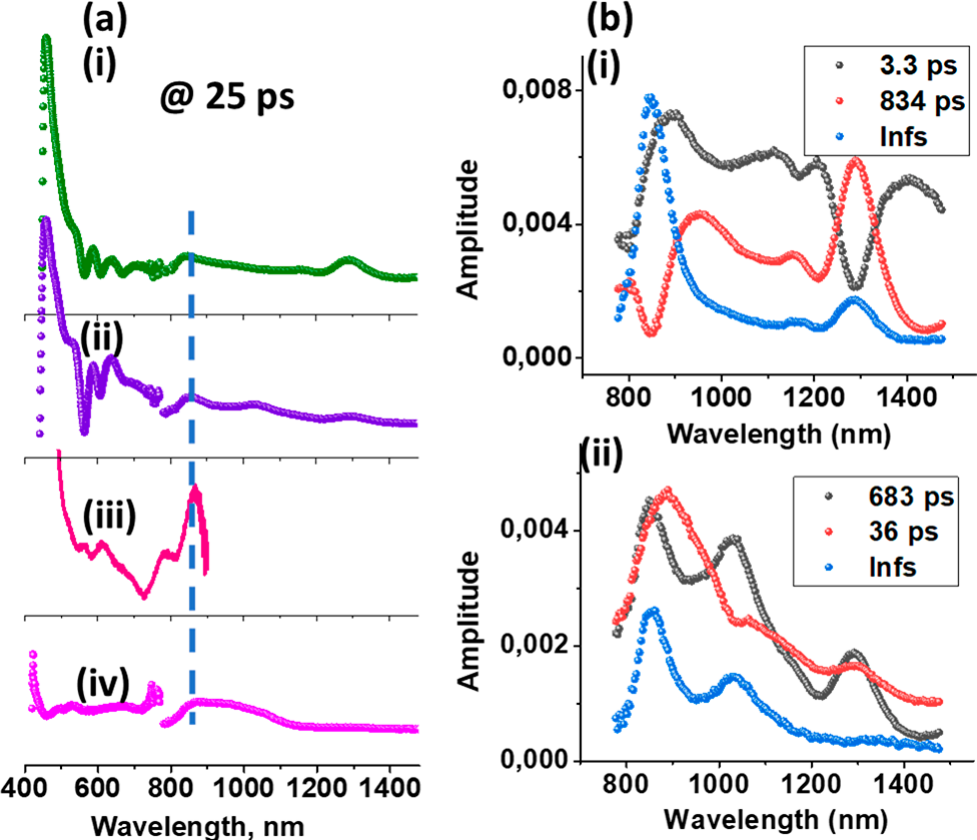
(a) The fs-TA spectrum at the delay time of 25 ps of (i) **7**, (ii) compound **1**, and (iv) compound **6**. Panel (iii) shows the spectrum of chemically generated ZnP^•+^. All spectra were recorded in deaerated benzonitrile. Porphyrin-containing compounds were excited at 435 nm, whereas compound **6** was excited at 363 nm. (b) Decay-associated spectra in the near-IR region for (i) **7** and (ii) compound **1**.

Femtosecond transient absorption spectra (fs-TA) of the control compounds **7** and **6**, along with **1** and **2**, are shown in Figure S46, whereas a snapshot of the spectrum collected at 25 ps delay time is shown in Figure 9a for comparison purposes. The instantaneously formed ^1^ZnP*–COOH revealed positive peaks at 458, 588, 641, and 1288 nm due to excited-state absorption (ESA), a negative peak at 564 due to ground-state bleaching (GSB), and two negative peaks at 607 and 665 nm due to stimulated emission (SE) of ^1^ZnP* (see Figure S46a and Figure 9a(i)). The 607 nm peak also had contributions from GSB. Decay of the positive peaks and recovery of the negative peaks was slow in accordance with relatively long-lived ^1^ZnP* and was accomplished by two new signals at 480 and 848 nm due to the formation of ^3^ZnP* via the process of intersystem crossing. Confirmation for the formation of ^3^ZnP* came from nanosecond transient absorption (ns-TA) spectral studies as shown in Figure S47a. The ^3^ZnP* decayed with a time constant of 15.4 μs. The fs-TA spectra of **6** are shown in Figure S46b and Figure 9a(iv). ESA peaks at 528 nm and a broad peak covering the 800–1100 nm range were observed. The decay of the ESA peaks of ^1^C_60_* was accompanied by a new peak at 690 nm due to the formation of ^3^C_60_*.^37^ The fs-TA spectral data of **7** and **6** were further analyzed by developing decay-associated spectra (DAS). The DAS of **7** as shown in Figure 9b(i) revealed three components. The 3.3 and 834 ps spectra with peaks at 890, 1112, 1205, and 1290 nm were mirror images representing formation and decay of ^1^ZnP*. The third spectrum revealed a major peak at 848 nm and a minor peak at 1290 nm. The 848 nm peak is consistent with the earlier discussed ^3^ZnP*. The DAS analysis of ^1^6* yielded a lifetime of 1.43 ns.

The fs-TA spectra of compounds **1** and **2** are shown in Figures S46c and S46d, respectively (also see Figure 9a(ii) for the spectrum of **1** at 25 ps). Peaks associated with the ^1^ZnP*’s ESA, GSB, and SE revealed faster decay/recovery with new transient peaks at 634, 855, and 1020 nm. The first two peaks agree well with the ZnP^•**+**^ spectrum (also see Figure 9a(iii) for the overlaid spectrum of ZnP^•**+**^), whereas the 1020 nm is consistent with the formation of C_60_^•–^, thus providing evidence for photoinduced electron transfer. As expected, the decay of ^1^ZnP* did not populate ^3^ZnP*, indicating that intersystem crossing is not the main deactivation path in **1** and **2**. The DAS generated for **1** revealed three components (Figure 9b(ii)). The first one at 36 ps had features of ^1^ZnP*, discussed earlier for **7**. The short time constant for this species is consistent with ^1^ZnP* undergoing faster deactivation due to the occurrence of electron transfer. The second component at 683 ps reveals features of both charge-separated state and ^1^ZnP* (note: compound **1** possesses two ZnP entities; only one ZnP entity is required to promote electron transfer), whereas the third DAS with infinity time constant (>3 ns) shows peaks at 856 and 1028 nm, expected for the ZnP^•**+**^–C_60_^•–^ charge-separated state in the near-IR region. DAS generated for compound **2** revealed three components (see Figure S46d inset). The first one at a time constant of 225 ps has negative peaks at 880 and 1095 nm (represent growth of new species) and positive peaks at 1185 and 1294 nm (represent decay), and the second DAS at 1.29 ns has peaks at 870 and 1030 nm. The third DAS with infinity time constant (>3 ns) reveals peaks expected for the ZnP^•**+**^–C_60_^•–^ charge-separated state. These results establish successful occurrence of excited-state electron transfer in **1** and **2**. The DAS at 683 ps in the case of **1** and 225 ps in the case of **2**, attributed to time constants for charge separation, resulted in a charge-separation rate constant (*k*_CS_) of 4.44 × 10^9^ s^–1^ for **1** and 1.46 × 10^9^ s^–1^ for **2**.

**Figure 10:**
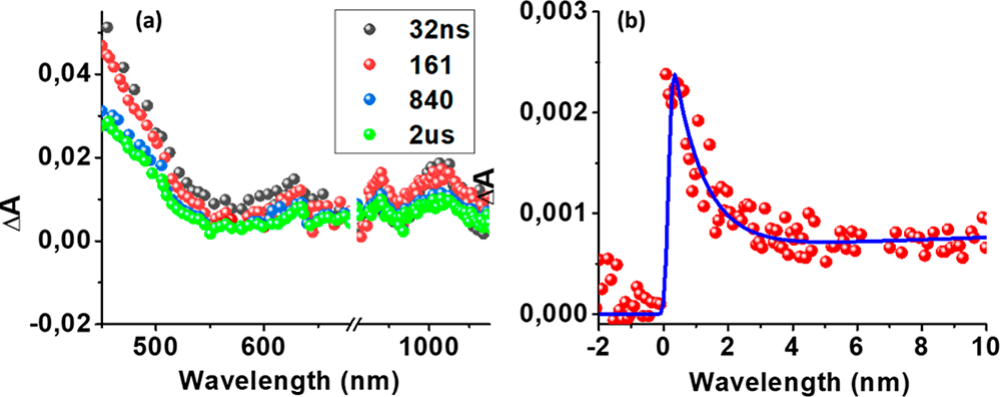
(a) Nanosecond transient absorption spectra of **1** at the indicated delay times in deaerated benzonitrile (λ_ex_ = 430 nm). (b) Time profile of the 1020 nm peak corresponding to C_60_^•–^.

The final DAS of both compounds **1** and **2** indicate persistence of charge-separated states beyond the monitoring time window of our fs-TA setup being 3 ns. Earlier-discussed AFM studies revealed large donut-shaped aggregate formation in the case of **1**. To find out the final lifetime and to see whether the aggregates could extend the lifetime of the charge-separated states, ns-TA spectral studies were performed. As shown in Figure S47c, the ns-TA spectrum of **2** in benzonitrile was largely featureless; that is, no peak supporting the existence of ZnP^•**+**^–C_60_^•–^ charge-separated state could be clearly seen. Indeed, weak features of ^3^ZnP* in the near-IR region were observed, indicating that the lifetime of the charge-separated state is <30 ns, the earliest detectable time window of our instrumental setup. In contrast, the ns-TA spectra of **1**, as shown in Figure 10a, revealed transient features of both radical cation and radical anion that lasted for few microseconds. The lifetime of the final charge-separated state, obtained from monitoring the decay of the 1020 nm peak (Figure 10b), was biexponential, with decay time constants of about 1 μs (major, ∼80%) and 40 μs (minor, ∼20%), indicating the formation of a long-lived charge-separated state in compound **1**. Increasing the concentration of **1** from the initial 1 mM to 2 or 3 mM, without compromising optical transparency, only slightly affected the final lifetime of the charge-separated states.

## Conclusions

The unprecedented results summarized here have successfully demonstrated how lifetime of the charge-separated state could be extended by means of organized molecular donor–acceptor assemblies built by using **1**. Compound **2**, having a single donor and a single acceptor entity, revealed lack of aggregation formation signifying the importance of the second ZnP in the formation of well-defined donut-shaped assemblies. Charge stabilization via electron delocalization in donor–acceptor assemblies having closely associated donors or acceptors is a well-known phenomenon.^38^ The present system differs, however, in that the organization pattern is much better defined compared to previous systems. Energy-optimized structures revealed intermolecular interactions between the donor and acceptor entities with a curvature needed to build the donut–aggregates. The primary forces directing such assembly were π···π and C–H···π interactions, along with short C–H···O contacts between *tert*-butyl hydrogen atoms and nitro groups, creating the curvature over a period of time, prompting the formation of donut structures exhibiting an impressive inner cavity size estimated as 23 nm, close to that observed in natural photosynthetic antenna systems. The lifetime of the final radical ion pair, on the order of 40 μs, is one of the highest reported to date, highlighting the significance of giant donor–acceptor structures for light energy harvesting applications. The current strategy of building functional, photoactive structures is expected to lead to the design of next generation of photocatalysts for light-to-fuel and light-to-commodity chemicals production. Our laboratories are currently looking into such applications.

## Supporting Information Available

Synthetic details, additional spectral, computational and transient absorption data (PDF)

## References

[ref1] Ariga K.; Kunitake T. Supramolecular Chemistry-Fundamentals and Applications: Advanced Textbook; Springer-Verlag: Berlin, 2006.

[ref2] Cragg P. J. Supramolecular Chemistry: From Biological Inspiration to Biomedical Applications; Springer: Netherlands, 2010.

[ref3] Lehn J. M. Supramolecular chemistry. Science 1993, 260, 1762–1763. 10.1126/science.8511582. 8511582

[ref4] Uhlenheuer D. A.; Petkau K.; Brunsveld L. Combining supramolecular chemistry with biology. Chem. Soc. Rev. 2010, 39, 2817–2826. 10.1039/b820283b. 20461247

[ref5] Cogdell R.; Mullineaux C. Photosynthetic Light Harvesting; Springer Nature: Switzerland, 2008. 10.1007/s11120-007-9270-1 18172745

[ref6] Blankenship R. E. Molecular Mechanisms of Photosynthesis; John Wiley & Sons: Chichester, 2008.

[ref7] Deisenhofer J.; Norris J. R. Photosynthetic Reaction Center; Academic Press: San Diego, CA, 2013; Vol. 2.

[ref8] Barber J. Photosynthetic energy conversion: Natural and artificial. Chem. Soc. Rev. 2009, 38, 185–196. 10.1039/B802262N. 19088973

[ref9] Fukuzumi S. Development of bioinspired artificial photosynthetic systems. Phys. Chem. Chem. Phys. 2008, 10, 2283–2297. 10.1039/b801198m. 18414719

[ref10] Hambourger M.; Moore G. F.; Kramer D. M.; Gust D.; Moore A. L.; Moore T. A. Biology and Technology for Photochemical Fuel Production. Chem. Soc. Rev. 2009, 38, 25–35. 10.1039/B800582F. 19088962

[ref11] Armaroli N.; Balzani V. Solar electricity and solar fuels: Status and perspectives in the context of the energy transition. Chem. - Eur. J. 2016, 22, 32–57. 10.1002/chem.201503580. 26584653

[ref12] Fukuzumi S.; Ohkubo K.; Suenobu T. Long-lived charge separation and applications in artificial photosynthesis. Acc. Chem. Res. 2014, 47, 1455–1464. 10.1021/ar400200u. 24793793

[ref13] Imahori H.; Mori Y.; Matano Y. Nanostructured artificial photosynthesis. J. Photochem. Photobiol., C 2003, 4, 51–83. 10.1016/S1389-5567(03)00004-2.

[ref14] Guldi D. M. Fullerene-porphyrin architectures; photosynthetic antenna and reaction Center models. Chem. Soc. Rev. 2002, 31, 22–36. 10.1039/b106962b. 12108980

[ref15] Gust D.; Moore T. A.; Moore A. L. Solar fuels via artificial Photosynthesis. Acc. Chem. Res. 2009, 42, 1890–1898. 10.1021/ar900209b. 19902921

[ref16] Wasielewski M. R. ‘Self-assembly strategies for integrating light harvesting and charge separation in artificial photosynthetic systems,’. Acc. Chem. Res. 2009, 42, 1910–1921. 10.1021/ar9001735. 19803479

[ref17] Fiechter S. Artificial photosynthesis - An inorganic approach. Adv. Bot. Res. 2016, 79, 99–128. 10.1016/bs.abr.2016.04.002.

[ref18] Bottari G.; de la Torre G.; Guldi D. M.; Torres T. Covalent and noncovalent phthalocyanine-carbon nanostructures systems. Chem. Rev. 2010, 110, 6768–6816. 10.1021/cr900254z. 20364812

[ref19] Aida T.; Meijer E. W.; Stupp S. I. Functional supramolecular polymers. Science 2012, 335, 813–817. 10.1126/science.1205962. 22344437 PMC3291483

[ref20] Ariga K.; Nishikawa M.; Mori T.; Takeya J.; Shrestha L. K.; Hill J. P. Self-assembly as a key player for materials nanoarchitectonics. Sci. Technol. Adv. Mater. 2019, 20, 51–95. 10.1080/14686996.2018.1553108. 30787960 PMC6374972

[ref21] D’Souza F.; Ito O. Photoinduced electron transfer in supramolecular systems of fullerenes functionalized with ligands capable of binding to zinc porphyrins and zinc phthalocyanines. Coord. Chem. Rev. 2005, 249, 1410–1422. 10.1016/j.ccr.2005.01.002.

[ref22] El-Khouly M. E.; Ito O.; Smith P. M.; D’Souza F. Intermolecular and supramolecular photoinduced electron transfer processes of fullerene-porphyrin/phthalocyanine systems. J. Photochem. Photobiol., C 2004, 5, 79–104. 10.1016/j.jphotochemrev.2004.01.003.

[ref23] Barrejon M.; Arellano L. M.; D’Souza F.; Langa F. Bidirectional charge transfer in carbon- based hybrid nanomaterials. Nanoscale 2019, 11, 14978–14992. 10.1039/C9NR04388H. 31372604

[ref24] KC C. B.; D’Souza F. Design and photochemical study of supramolecular donor-acceptor systems assembled via metal-ligand axial coordiation. Coord. Chem. Rev. 2016, 322, 104–141. 10.1016/j.ccr.2016.05.012.

[ref25] Adhikari S.; Ghosh A.; Mandal S.; Guria S.; Banerjee P. P.; Chatterjee A.; Das D. Colorimetric and fluorescence probe for the detection of nano-molar lysine in aqueous medium. Org. Biomol. Chem. 2016, 14, 10688–10694. 10.1039/C6OB01704E. 27801458

[ref26] aCuesta V.; Urbani M.; de la Cruz P.; Welte L.; Nierengarten J. F.; Langa F. Regioselective preparation of a bis-pyrazolinofullerene by a macrocyclization reaction. Chem. Commun. 2016, 52, 13205–13208. 10.1039/C6CC06549J. 27713942

[ref27] Because of the dissymmetry of the pyrazolino rings, the number of conceivable regioisomeric bisadducts is doubled. Out and in denote the relative position of the *p*-nitrophenyl groups. All possible isomers of the bisadduct are depicted in Figure S33.

[ref28] Prato M.; Soombar C.; Vazquez E.; Niziol J.; Gondek E.; Rau I.; Kajzar F. Synthesis and Spectroscopic Properties of Porphyrin Derivatives of C_60_. Mol. Cryst. Liq. Cryst. 2010, 521, 253–264. 10.1080/15421401003726907.

[ref29] aLiu Y.; Wang Z.; Zhang X. Characterization of supramolecular polymers. Chem. Soc. Rev. 2012, 41, 5922–5932. 10.1039/c2cs35084j. 22674180

[ref30] aHaino T.; Fujii T.; Watanabe A.; Takayanagi U. Supramolecular polymer formed by reversible self-assembly of tetrakisporphyrin. Proc. Natl. Acad. Sci. U. S. A. 2009, 106, 10477–10481. 10.1073/pnas.0809602106. 19289843 PMC2705564

[ref31] Spicher S.; Grimme S. Robust Atomistic Modeling of Materials, Organometallic, and Biochemical Systems. Angew. Chem., Int. Ed. 2020, 59, 15665–15673. 10.1002/anie.202004239. PMC7267649 32343883

[ref32] Bannwarth C.; Ehlert S.; Grimme S. GFN2-xTB—An Accurate and Broadly Parametrized Self-Consistent Tight-Binding Quantum Chemical Method with Multipole Electrostatics and Density-Dependent Dispersion Contributions. J. Chem. Theory Comput. 2019, 15, 1652–1671. 10.1021/acs.jctc.8b01176. 30741547

[ref33] aSmith P. M.; McCarty A. L.; Nguyen N. Y.; Zandler M. E.; D’Souza F. D. Bis-functionalized fullerene-dibenzo[18]crown-6 conjugate: synthesis and cation-complexation dependent redox behavior. Chem. Commun. 2003, 1754–1755. 10.1039/b303127f. 12877537

[ref34] Becke A. D. Density-functional thermochemistry. III. The role of exact exchange. J. Chem. Phys. 1993, 98, 5648–5652. 10.1063/1.464913.

[ref35] Risthaus T.; Grimme S. Benchmarking of London Dispersion-Accounting Density Functional Theory Methods on Very Large Molecular Complexes. J. Chem. Theory Comput. 2013, 9, 1580–1591. 10.1021/ct301081n. 26587619

[ref36] Rehm D.; Weller A. Kinetics of Fluorescence Quenching by Electron and Hydrogen-Atom Transfer. Isr. J. Chem. 1970, 8, 259–271. 10.1002/ijch.197000029.

[ref37] Guldi D. M.; Kamat P. V. Photophysical Properties of Pristine Fullerenes, Functionalized Fullerenes, and Fullerene-Containing Donor-Bridge Acceptor Systems. In Fullerenes; Wiley: New York, 2000; pp 225–281.

[ref38] aFukuzumi S.; Saito K.; Ohkubo K.; Khoury T.; Kashiwagi Y.; Absalom M. A.; Gadde S.; D’Souza F.; Araki Y.; Ito O.; Crossley M. J. Multiple photosynthetic reaction centres composed of supramolecular assemblies of zinc porphyrin dendrimers with a fullerene acceptor. Chem. Commun. 2011, 47, 7980–7982. 10.1039/c1cc11725d. 21681311

