# Supplementary material for: Self-Assembly-Directed Organization of a Fullerene–Bisporphyrin into Supramolecular Giant Donut Structures for Excited-State Charge Stabilization
Source: J Am Chem Soc. 2021 Jul 14;143(29):11199–208. doi: 10.1021/jacs.1c05133 (PMC8397305; doi:10.1021/jacs.1c05133)
Supplement: Supplementary file 1 — ja1c05133_si_001.pdf [file ja1c05133_si_001.pdf]

Supporting Information

Self-Assembly-Directed Organization of a Fullerene-Bisporphyrin into Supramolecular Giant Donut Structures for Excited-State Charge Stabilization

Rubén Caballero,[a] Myriam Barrejón,[a,b] Jesús Cerdá,[c] Juan Aragó,[c] Sairaman Seetharaman,[d] Pilar de la Cruz,[a] Enrique Ortí,[c,*] Francis D'Souza,[d,*] Fernando Langa.[a,*]

[a]*Instituto de Nanociencia, Nanotecnología y Materiales Moleculares (INAMOL), Universidad de Castilla-La Mancha, Campus de la Fábrica de Armas, 45071 Toledo, Spain;* *fernando.langa@uclm.es*

[b]*Neural Repair and Biomaterials Laboratory, Hospital Nacional de Parapléjicos (SESCAM), Finca la Peraleda s/n, 45071 Toledo, Spain.*

[c]*Instituto de Ciencia Molecular, Universidad de Valencia, 46950 Paterna, Spain;* *enrique.orti@uv.es*

[d]*Department of Chemistry, University of North Texas, 1155 Union Circle, #305070, Denton, Texas TX 76203-5017, United States;* *francis.dsouza@unt.edu*

Table of contents

I.General Information........ S2
II.Synthesis........ S3
III.Collection of spectra........ S10
IV.Theorethical calculations........ S29
V.Photophysical measurements........ S37

I. General Information

C_{60} (+99.95%) was purchased from BuckyUSA (Bellaire, TX). Solvents and chemicals were purchased from Aldrich Chemicals (Milwaukee, WI). Anhydrous solvents, when indicated, were dried using a Pure-Sov 400 or using standard techniques. Chromatographic purifications were performed using silica gel 60 VWR (particle size 0.040–0.063 mm). Analytical thin-layer chromatography was performed using Merck (TLC) silica gel 60 F254. ^{1}H NMR spectra were recorded for solutions in a partial deuterated solvent on a Brüker-Topspin AV 400 instrument. Chemical shifts are given as δ values. ^{1}H NMR chemical shifts are reported relative to residual non deuterated solvent peaks. ^{13}C NMR chemical shifts are reported relative to the deuterated solvent peak. FT-IR spectra were recorded in an AVATAR 370 FT-IR Thermo Nicolet spectrometer. Mass spectra (MALDI-TOF) were recorded on a VOYAGER DETM STR mass spectrometer using dithranol as matrix or on a ULTRAFLEX III TOF/TOF, using DCTB as matrix. Mass spectra (ESI-QTOF) were recorded on a 3200 QTRAP LC-MS/MS. The main peaks are expressed as *m/z*. Steady state UV/Vis spectra were recorded on a Shimadzu UV-VIS-NIR spectrophotometer UV-3600 in quartz cuvettes with a path length of 1 cm. The emission measurements were carried out on a Cary Eclipse fluorescence spectrophotometer. Cyclic and Oyster-Young square-wave voltammetries were performed in a µAUTOLAB Type II potentiostat, using 0.1M solution of tetrabutylammonium hexafluorophosphate in 1,2-dichlorobenzene:acetonitrile 4:1 as a solvent. Solutions were deoxygenated by bubbling argon through prior to each measurement. Experiments were carried out in a one-compartment cell equipped with a glassy carbon electrode, a platinum wire counter electrode, and an $Ag/AgNO_3$ wire as pseudo-reference electrode. All Potentials were checked against the ferrocene/ferrocenium couple (Fc/Fc^+) after each experiment. AFM images were recorded in tapping mode using a Multimode 8 system (Veeco Instruments Inc., Santa Barbara, USA) with a NanoScope V controller (Digital Instruments, Santa Barbara, USA) operating at room temperature in ambient air conditions. RTESP-300 Bruker silicon cantilevers with a resonance frequency of 300 kHz and a nominal force constant of 40 N m^{-1} were used for AFM measurements. The images were processed using WSxM[1] (freely downloadable scanning probe microscopy software from http://www.wsxmsolutions.com).

II. Synthesis

Synthesis of 3-hydroxymethylphenyl-p-nitrophenylhydrazone (3).

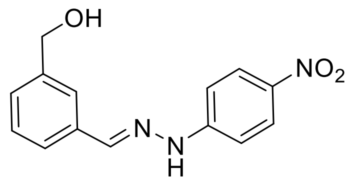

Under argon atmosphere, over a stirred solution of 3-hydroxymethylbenzaldehide[2] (0.330 g, 2.40 mmol) and 4-nitrophenylhydrazine (0.37 gr, 2.40 mmol) in 35 mL of ethanol under reflux, two drops of glacial acetic acid are added. The reaction mixture is stirred 10 minutes, then cooled to 0 °C and the formed solid is filtered and recrystallized in ethanol to obtain **3** as an orange solid (0.59 g, 91%). ^{1}H-NMR (DMSO-D$_6$, 298K) δ (ppm): 11.30 (s, 1H), 8.14 (d, 2H, ^{3}J = 9 Hz), 8.05 (s, 1H), 7.70 (s, 1H), 7.58 (d, 1H, ^{3}J = 8 Hz), 7.38 (t, 1H, ^{3}J = 8 Hz), 7.32 (d, 1H, ^{3}J = 8 Hz), 7.17 (d, 2H, ^{3}J = 9 Hz), 5.29 (t, 1H, ^{3}J = 5.7 Hz), 4.55 (d, 2H, ^{3}J = 5.7 Hz). ^{13}C NMR (DMSO-D$_6$, 298K) δ (ppm): 151.06, 143.64, 142.52, 138.82, 134.96, 129.02, 126.69, 125.60, 124.67, 111.72, 63.12. FT-IR (ATR) ν (cm^{-1}): 1600 (C=N), 1491 (NO_2) 1328 (NO_2). UV-Vis (CH_2Cl_2) λ_{max} (nm) (log ε): 390 (4.39). EM (ESI Q-TOF) (*m/z*): calculated for $C_{14}H_{13}N_3O_3$: 271.1; found: 272.1 (M-H)$^+$.

Synthesis of 4.

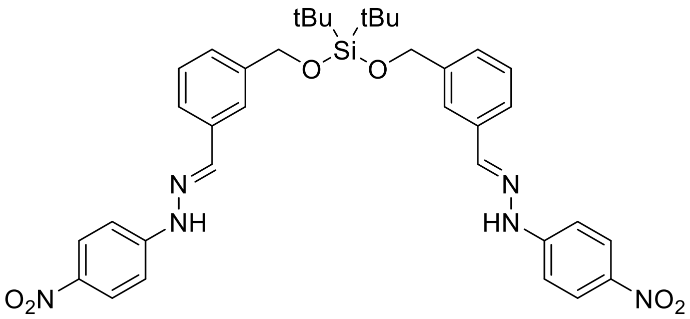

Under argon atmosphere, over a stirred solution of **3** (0.1 g, 0.36 mmol) and anhydrous pyridine (0.03 mL, 0.36 mmol), in 10 mL of anhydrous dimethylformamide at 0 °C, di-*tert*-butylsilylbis(trifluoromethanesulfonate) (0.081 g, 0.18 mmol) is added dropwise and the mixture is stirred at room temperature for 12 hours. Next, the solvent is removed under reduced pressure and the crude is purified by column chromatography (SiO_2, hexane:ethyl acetate 1:2). The obtained solid is recrystallized in dichloromethane affording **4** as a red solid (0.063 g, 50%). ^{1}H-NMR (acetone-D$_6$, 298K) δ (ppm): 10.26 (s, 1H), 8.11 (d, 2H, ^{3}J = 12 Hz), 8.01 (s, 2H), 7.88 (s, 2H), 7.57 (d, 2H, ^{3}J =12 Hz), 7.41 (t, 4H, ^{3}J = 8 Hz), 7.22 (d, 4H, ^{3}J = 8 Hz), 5.10 (s,4H), 1.20 (s, 18H). ^{13}C-NMR (acetone-D$_6$, 298K) δ (ppm): 150.59, 141.88, 141.59, 139.52, 135.13, 128.64, 125.78,

125.74, 123.34, 111.37, 65.24, 25.74, 21.18. FT-IR (ATR) ν (cm^{-1}): 1590 (C=N), 1483 (NO_2), 1309 (NO_2),1097 (Si-O). UV-Vis (CH_2Cl_2) λ_{max} (nm) (log ε): 390 (4.51).

Synthesis of 5.

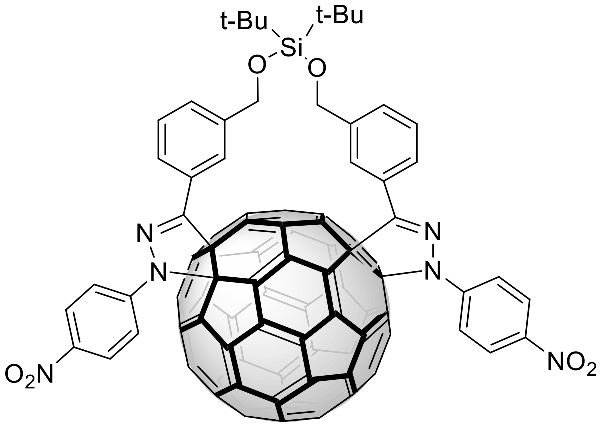

Under argon atmosphere, over a stirred solution of **3** (133 mg, 0.19 mmol) in 30 mL of anhydrous THF, *N*-bromosuccinimide (0.133 g, 0.76 mmol) is added. The mixture is stirred at room temperature for 30 minutes, then the solvent is evaporated and a solution of C_{60} (0.137 g, 0.19 mmol) in 137 mL of anhydrous toluene is added. Under bubbling argon, triethylamine (0.077 g, 0.76 mmol) is added and the reaction mixture is stirred at 40 °C for 12 hours. After removing the solvent under reduced pressure, the crude is purified by column chromatography (SiO_2, carbon disulphide:toluene 1:1). The fraction corresponding to the fullerene bisadduct is further purified by preparative HPLC (Buckyprep 4.6 IDx250 mm, toluene 2 mL/min). The obtained solid is washed several times with ethanol and diethylether to obtain a mixture of regioisomers of **5** as a dark brown solid (80 mg, 35%). Recycling HPLC allowed the separation of the pure *e*-isomer of **5** (named **5a**) and a mixture of other two isomers (named **5b** and **5c**) as ^{1}H NMR evidence. ^{1}H NMR (CD_2Cl_2, 298K) δ (ppm) for **5a**: 8.29 (d, 2H, ^3J = 7 Hz), 8.16 (d, 2H, ^3J = 7 Hz), 8.14 (d, 2H, ^3J = 7 Hz), 8.04 (d, 2H, ^3J = 7 Hz), 7.88 (s, 1H), 7.85 (s, 1H), 7.79 (d, 1H, ^3J = 8 Hz), 7.70 (d, 1H, ^3J = 8 Hz), 7.57 (d, 1H, ^3J = 8 Hz), 7.52 (t, 1H, ^3J = 8 Hz), 7.41 (t, 1H, ^3J = 8 Hz), 7.30 (d, 1H, ^3J = 8 Hz), 5.19 (d, 1H, ^2J = 14 Hz), 5.11 (d, 2H, ^2J = 14 Hz), 4.98 (d, 1H, ^2J = 14 Hz), 1.19 (s, 1H). 0.93 (s, 1H). δ (ppm) for **5b**: 8.51 (d, 4H, ^3J = 9 Hz), 8.42 (d, 4H, ^3J = 9 Hz), 7.93 (d, 2H, ^3J = 8 Hz), 7.79 (s, 2H), 7.55 (t, 2H, ^3J = 8 Hz), 7.42 (d, 2H, ^3J = 8 Hz), 4.95 (d, 2H, ^2J = 13 Hz), 4.78 (d, 2H, ^2J = 13 Hz), 0.87 (s, 18H). FT-IR (KBr) ν (cm^{-1}): 1587 (NO_2), 1331 (NO_2), 1039 (Si-O), 527 (C_{60}). EM (*m/z*) (MALDI TOF): calculated for $C_{96}H_{38}N_6O_6Si$: 1398.26; found: 1398.40 (M^+).

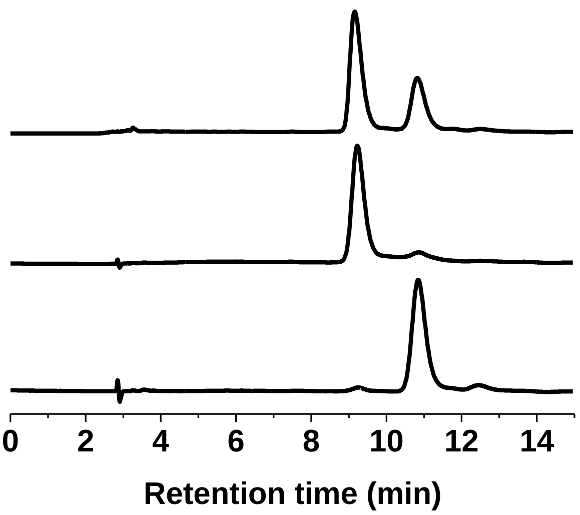

Figure S1. HPLC profiles of the mixture of isomers of **5** (top) and of the purified fractions of **5a** (middle) and **5b/5c** (bottom) (column: Buckyprep 4.6IDx250 mm, solvent: toluene 1 mL/min).

Synthesis of 6.

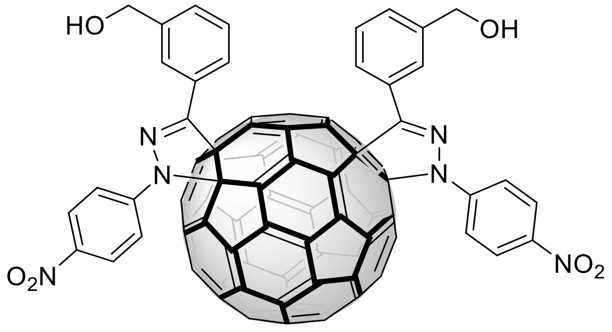

Under argon atmosphere, over a stirred solution of a mixture of regioisomers of **5** (0.1 g, 0.071 mmol) in 30 mL of dichloromethane:acetonitrile 2:1, boron trifluoro etherate (48%, 1 mL, 2.86 mmol) is added and the reaction mixture is stirred at room temperature for 36 h. After this time, a saturated solution of ammonium chloride in water (30 mL) is added, the phases are separated and the organic phase washed several times with water, dried, and the solvent evaporated under reduced pressure. The crude obtained is purified by column chromatography (SiO_2, CS_2:ethyl acetate 9:1) to obtain a single isomer of **6** as a brown solid. (18 mg, 20%). ^{1}H-NMR (400 MHz, $CDCl_3$:CS_2 1:1, 298K) δ (ppm): 8.34 (d, 2H, ^3J =9 Hz), 8.23 (d, 2H, ^3J =9 Hz), 8.16 (d, 2H, ^3J =9 Hz), 8.11 (s, 1H), 8.08 (d, 2H, ^3J =9 Hz), 8.02 (s, 1H), 7.93 (d, 1H, ^3J =6 Hz), 7.50 (t, 1H, ^3J=8Hz), 7.44 (m. 4H), 4.80 (d, 2H, ^2J =13 Hz), 4.75 (s, 2H) 4.75 (d, 2H, ^2J=13 Hz), 3,50 (s, 2H). ^{13}C NMR (100 MHz, $CDCl_3$:CS_2 1:1, 298K) δ (ppm): 166.35, 149.85, 149.63, 149.46, 149.09, 147.93, 147.26, 146.62, 146.22, 145.42, 145.10, 144.38, 143.55, 152.87, 142.58, 142.40, 141.87, 141.77, 141.61, 141.27, 140.78, 140.32, 137.74, 136.73, 136.63, 131.57, 131.27, 129.76, 129.31, 128.91, 128.67, 128.57, 127.95, 127.88, 126.43, 125.44, 125.28, 1198.44, 119.22, 89.14, 81.59, 64.85, 64.70. FT-

IR (KBr) ν(cm^{-1}): 1587(NO_2), 1328 (NO_2). UV-Vis (CH_2Cl_2) λ_{max} (nm), (log ε): 257 (4.65), 384 (3.95). MS (*m/z*) (MALDI TOF): calculated for $C_{88}H_{22}N_6O_6$: 1258.16; found: 1258.44 (M+); 989.21; 720 (C_{60}).

Synthesis of 1.

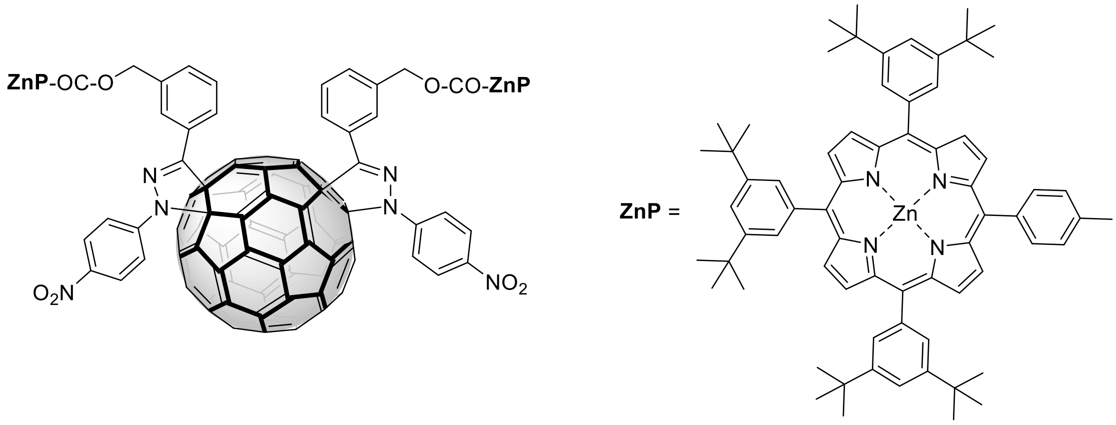

Under argon atmosphere, over a stirred solution of ZnP-COOH **(7)**[3] (0.063 g, 0.06 mmol) in 10 mL of anhydrous dichloromethane at 0 °C, 4-dimethylaminopyridine (0.024 g, 0.2 mmol) and 1-ethyl-3-(3-dimethylaminopropyl)carbodiimide hydrochloride (0.024 g, 0.12 mmol) are added successively. Then, a suspension of **6** (0.020 g, 0.02 mmol) in 5 mL of dry dichloromethane is added and the mixture is allowed to reach room temperature and stirred for 24 h. Next, the solvent is evaporated and the crude purified by column chromatography (SiO_2, toluene) and gel permeation chromatography (Biobeads SX1, Dichloromethane) to obtain **1** as a purple solid (15 mg, 30%). ^{1}H-NMR (400 MHz, $CDCl_3$, 298K) δ (ppm): 9.00 (m, 10H), 8.94 (d, 2H, ^3J =5 Hz), 8.78 (d, 2H, ^3J=5 Hz), 8.71 (d, 2H, ^3J =5 Hz), 8.2-7.9 (m, 20H), 8.08 (m, 12H), 7.85-7.70 (m, 6H), 7.62 (d, 1H, ^3J =8 Hz), 7.56 (t, 1H, ^3J = 8 Hz), 7.45 (t, 1H, ^3J = 8 Hz), 7.38 (d, 1H, ^3J =8 Hz), 5.24 (d, 1H, ^2J=12Hz), 5.15 (d, 1H, ^2J =12 Hz), 4.95 (d, 1H, ^2J =13 Hz), 4.82 (d, 1H, ^2J =13 Hz), 1.56-1.43(m, 108H). ^{13}C-NMR (100 MHz, $CDCl_3$, 298K) δ (ppm): 166.28, 166.10, 154.78, 150.56, 150.40, 150.30, 149.21, 148.52, 148.44. 148.17, 144.56, 142.45, 142.30, 141.8, 141.7, 141.20, 140.12, 136.84, 134.31, 132.53, 132.3, 131.6, 129.68, 129.55, 128.71, 127.61, 125.21, 122.62, 120.81, 119.65, 119.08, 118.81,89.14, 88.92, 81.25, 81.10, 66.30, 65.85, 35.01, 34.95, 31.75, 31.69. FT-IR (KBr) ν(cm^{-1}): 2952 (^{t}Bu), 1714 (C=O), 1594 (NO_2), 1334 (NO_2). UV-Vis (CH_2Cl_2) λ_{max} (nm), (log ε): 422 (5.74), 549 (4.47), 587(3.99). MS (*m/z*) (MALDI TOF): calculated for $C_{226}H_{170}N_{14}O_8Zn_2$: 3335.2; found: 3335.2 (M^+); 2439.4.

Synthesis of 2

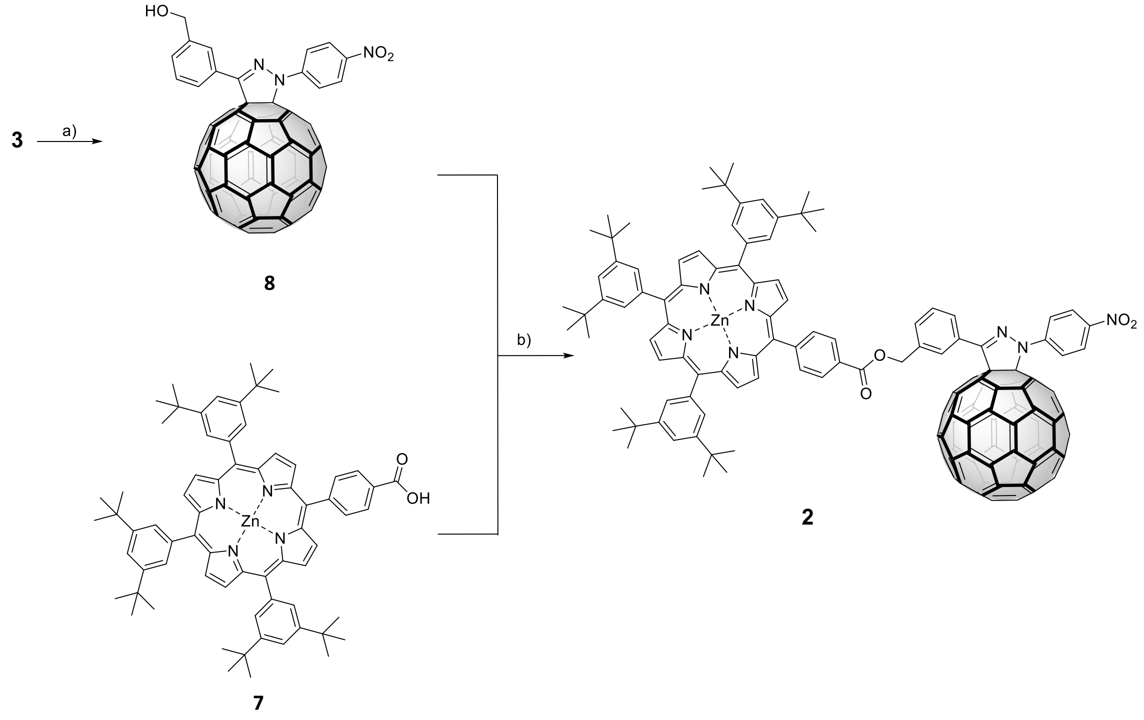

Scheme S1. Reactions and conditions a) i: NBS, $CHCl_3$, 30 min. ii:C_{60}, $(Et)_3N$, Toluene, 2h, 60 °C, 35%. b) EDCI, DMAP, CH_2Cl_2, r.t. 17 h, 67%.

Synthesis of 8.

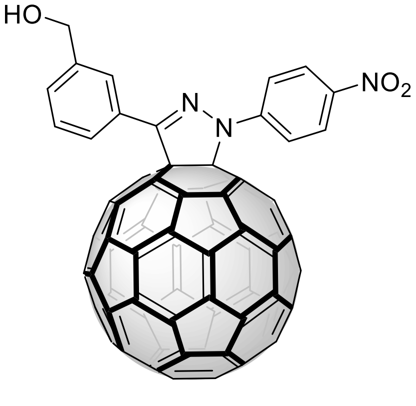

Under argon atmosphere, over a stirred solution of **3** (30 mg, 0.11 mmol) in 20 mL of anhydrous THF, *N*-bromosuccinimide (0.038 g, 0.22 mmol) is added. The mixture is stirred at room temperature for 30 m, then the solvent is evaporated and a solution of C_{60} (160 mg, 0.022 mmol) in 50 mL of anhydrous toluene is added. Under bubbling argon, triethylamine (0.010 g, 0.11 mmol) is added and the reaction mixture is stirred at 40 °C for 2 h. Next, the solvent is evaporated under reduced pressure and the crude is purified by column chromatography (SiO_2, CS_2:toluene 1:1). The obtained solid is washed several times with ethanol and diethylether yielding **8** as a dark brown solid (42 mg, 39%). ^{1}H-NMR ($CDCl_3$:CS_2 1:1, 298K) δ (ppm): 8.33 (d, 2H, ^3J = 9 Hz), 8.28 (d, 2H, ^3J = 9 Hz), 8.23 (s, 1H), 8.14 (d, 1H, ^3J = 6 Hz), 7.53 (m, 2H), 4.84 (s, 2H).

^{13}C-NMR ($CDCl_3$:CS_2 1:1, 298K) δ (ppm): 149.67, 147.72, 147.23, 146.9, 146.51, 146.45, 146.15, 146.03, 145.70, 145.65, 145.48, 145.35, 144.62, 144.42, 144.19, 143.28, 143.08, 143.01, 142.51, 142.39, 142.20, 142.07, 141.92, 140.55, 139.5, 137.09, 136.23, 131.76, 129.15, 128.56, 128.29, 128.13, 127.83, 125.41, 120.81, 119.38, 119.38, 90.53, 82.57, 65.98, 15.41. FT-IR (KBr) ν(cm^{-1}): 1587 (NO_2), 1321 (NO_2), 527 (C_{60}). UV-Vis (CH_2Cl_2) λ_{max} (nm), (log ε): 317 (4.68), 380 (4.59). MS (*m/z*) (MALDI TOF): calculated for $C_{74}H_{11}N_3O_3$: 989.08; found: 989.57 (M+), 720.41 (C_{60}).

Synthesis of 2.

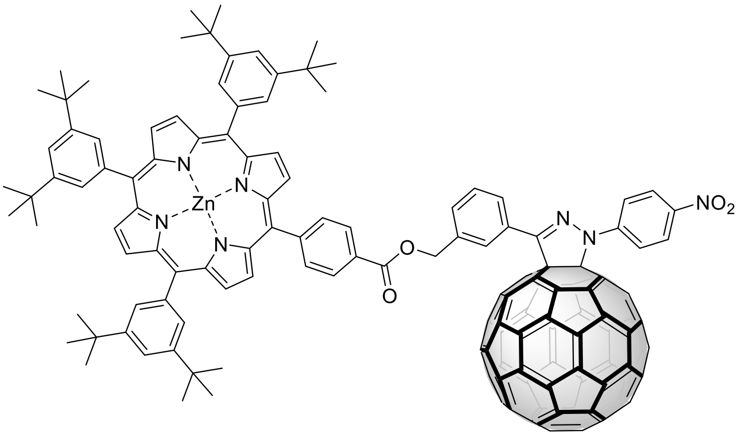

Under argon atmosphere, to a stirred solution of ZnPor-COOH (**7**)[3](0.063 g, 0.06 mmol) in 10 mL of anhydrous dichloromethane at 0 °C, 4-dimethylaminopyridine (0.015 g, 0.124 mmol) and 1-ethyl-3-(3-dimethylaminopropyl)carbodiimide hydrochloride (0.024 g, 0.12 mmol) are added successively. Then, a suspension of **8** (0.020 g, 0.030 mmol) in 5 mL of dry dichloromethane is added and the mixture is allowed to reach room temperature and stirred for 24 hours. Next, the solvent is evaporated and the crude purified by column chromatography (SiO_2, toluene) and gel permeation chromatography (Biobeads SX1, Dichloromethane) to obtain **2** as a purple solid (40 mg, 67%). ^{1}H-NMR (400 MHz, $CDCl_3$, 298K) δ (ppm): 8.99 (m, 4H), 8.93 (d, 2H, ^3J=5 Hz), 8.77 (d, 2H, ^3J=5 Hz), 8.37 (s, 1H), 8.3-8.1 (m, 8H), 8.08 (m, 2H) 8.05 (m, 4H), 7.79 (m, 3H), 7.70 (d, 1H, , ^3J=8 Hz), 7.61 (t, 1H, , ^3J=8 Hz), 7.56 (d, 1H, , ^3J=8 Hz), 5.53 (s, 2H), 1.51 (m, 54H). ^{13}C-NMR (100 MHz, $CDCl_3$, 298K) δ (ppm): 166.49, 150.60, 150.45, 150.29, 149.56, 149.50, 149.29, 148.59, 148.40, 147.67, 147.60, 147.07, 146.41, 145.72, 145.57, 145.29, 145.09, 144.98, 144.74, 144.17, 143.97, 143.80, 143.53, 143.16, 142.58, 142.37, 142.29, 142.18, 141.92, 141.75, 141.64, 141.60, 141.52, 141.32, 140.10, 139.98, 138.97, 138.70, 138.52, 138.43, 137.07, 137.00, 136.65, 135.87, 135.64, 134.36, 132.59, 132.48, 132.33,131.87, 131.73, 131.18, 129.98, 129.58, 129.41, 129.18, 129.11, 128.72, 128.63, 127.95, 125.37, 124.48, 124.01, 123.06, 122.73, 120.91, 119.31, 119.10, 118.98, 90.19, 82.06, 66.44, 35.09. FT-IR (KBr) ν(cm^{-1}): 2955 (^{t}Bu), 1717 (C=O), 1590 (NO_2), 1328

(NO_2), 527 (C_{60}). UV-Vis (CH_2Cl_2) λ_{max} (nm), (log ε): 316 (4.58), 421 (5.39), 549 (4.08), 587(3.58). MS (*m/z*) (MALDI TOF): calculated for $C_{143}H_{85}N_7O_4Zn$: 2030.7; found: 2030.3 (M^+).

III. Collection of spectra

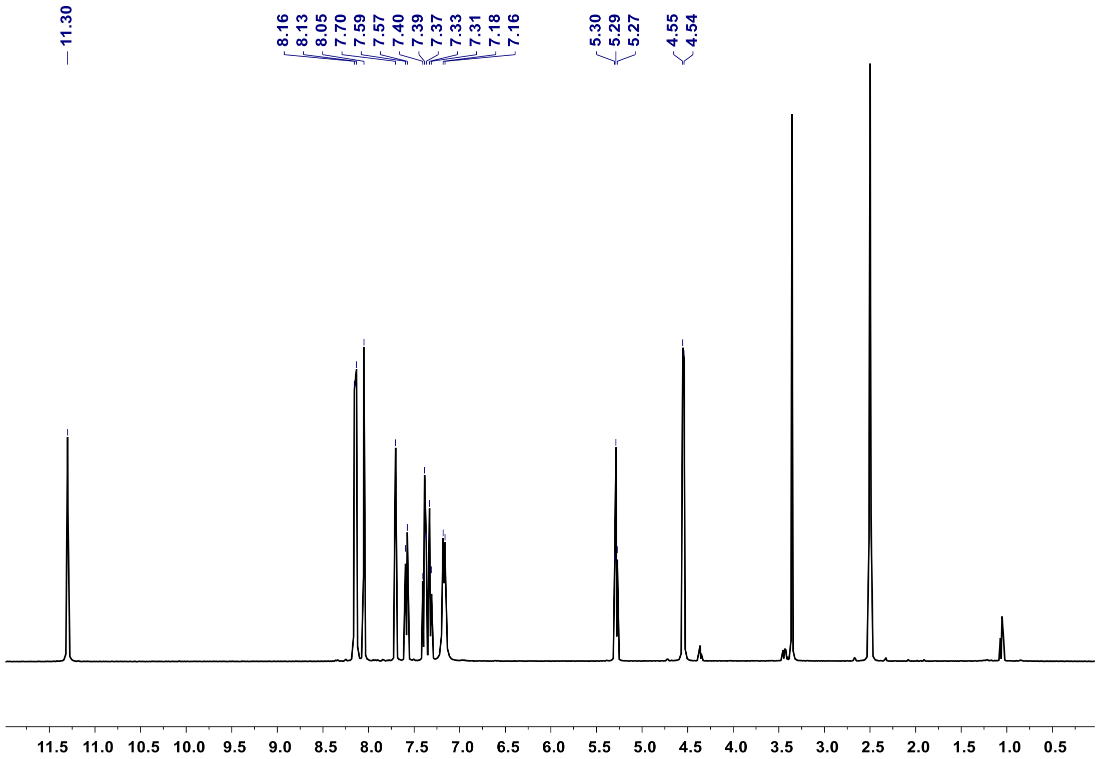

Figure S2. ^{1}H-NMR (400 MHz, DMSO-D$_6$, 298 K) spectrum of **3.**

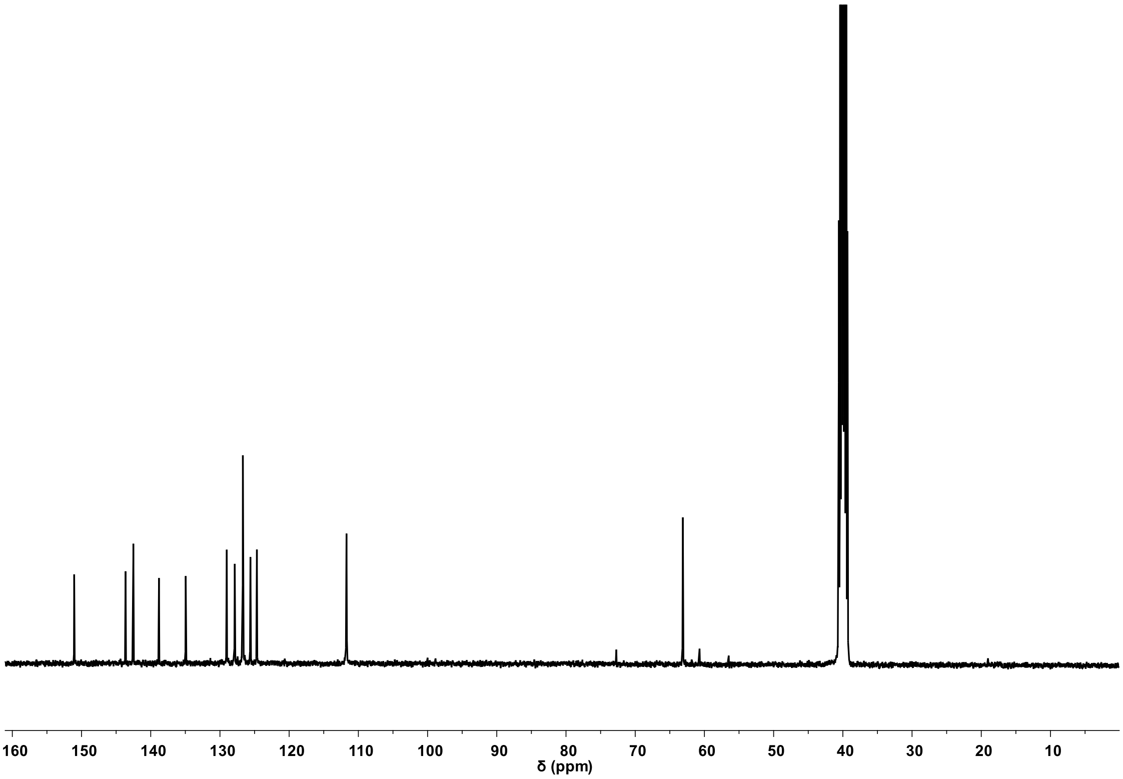

Figure S3. ^{13}C-NMR (400 MHz, DMSO-D$_6$, 298 K) spectrum of **3**.

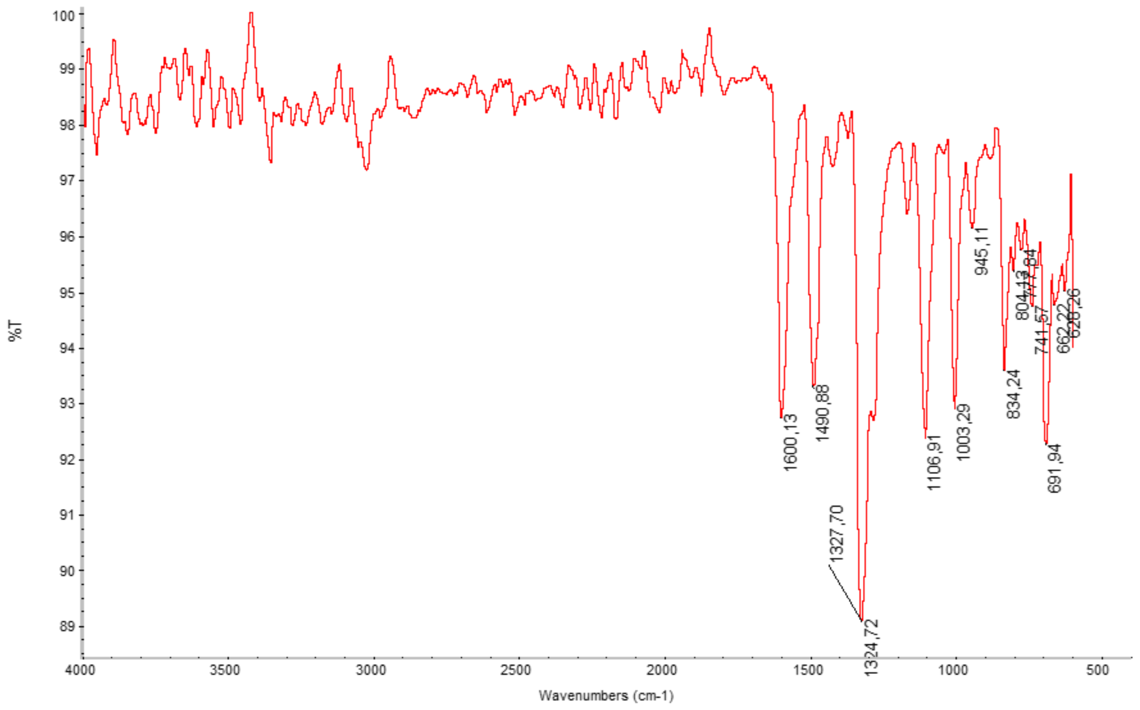

Figure S4. FT-IR (neat) spectrum of **3**.

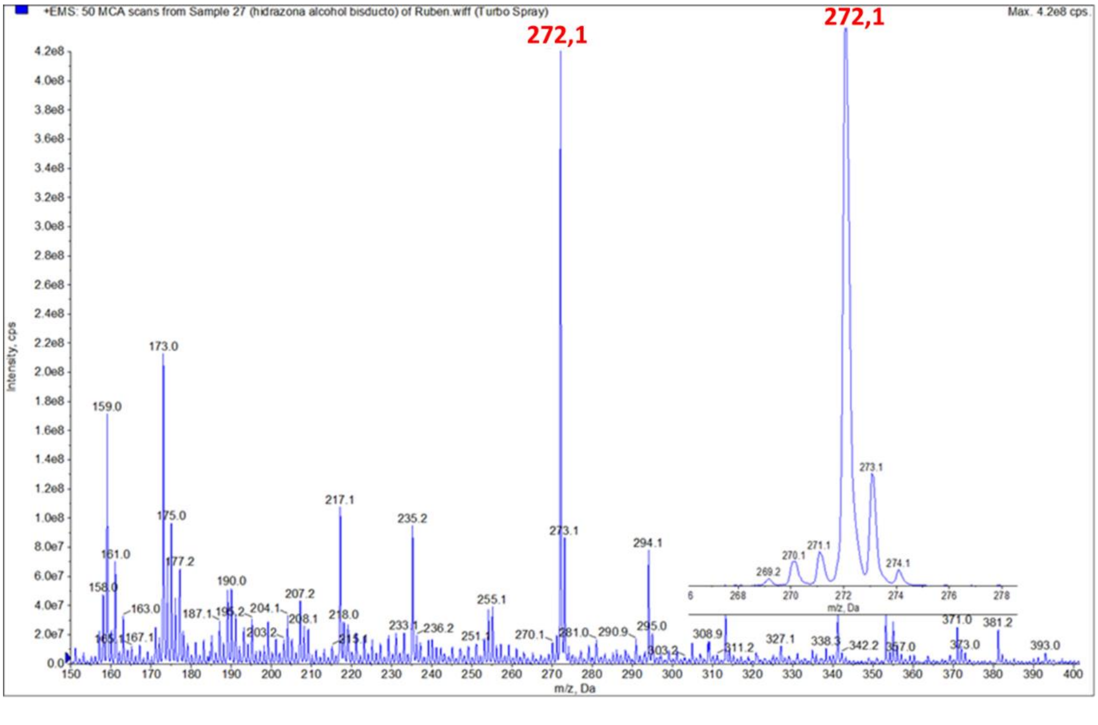

Figure S5. MS (ESI-QTOF) spectrum of **3**.

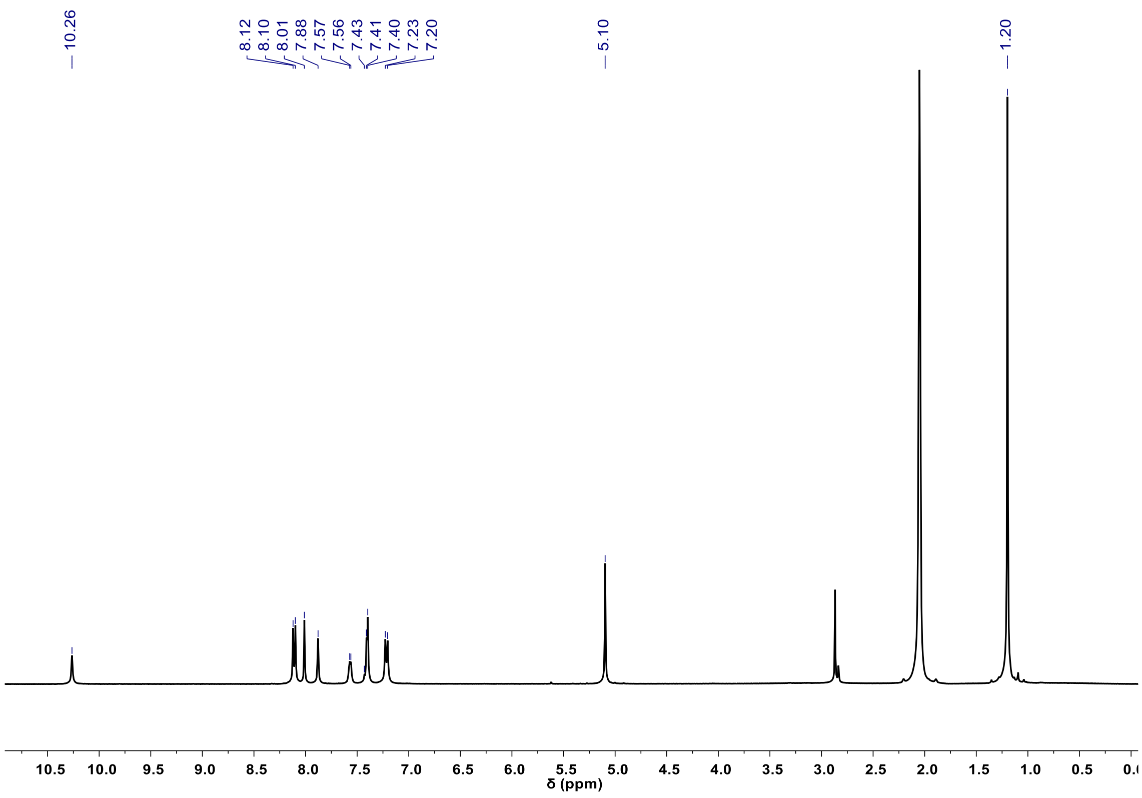

Figure S6. ^{1}H-NMR (400 MHz, Acetone-D$_6$, 298 K) spectrum of **4**.

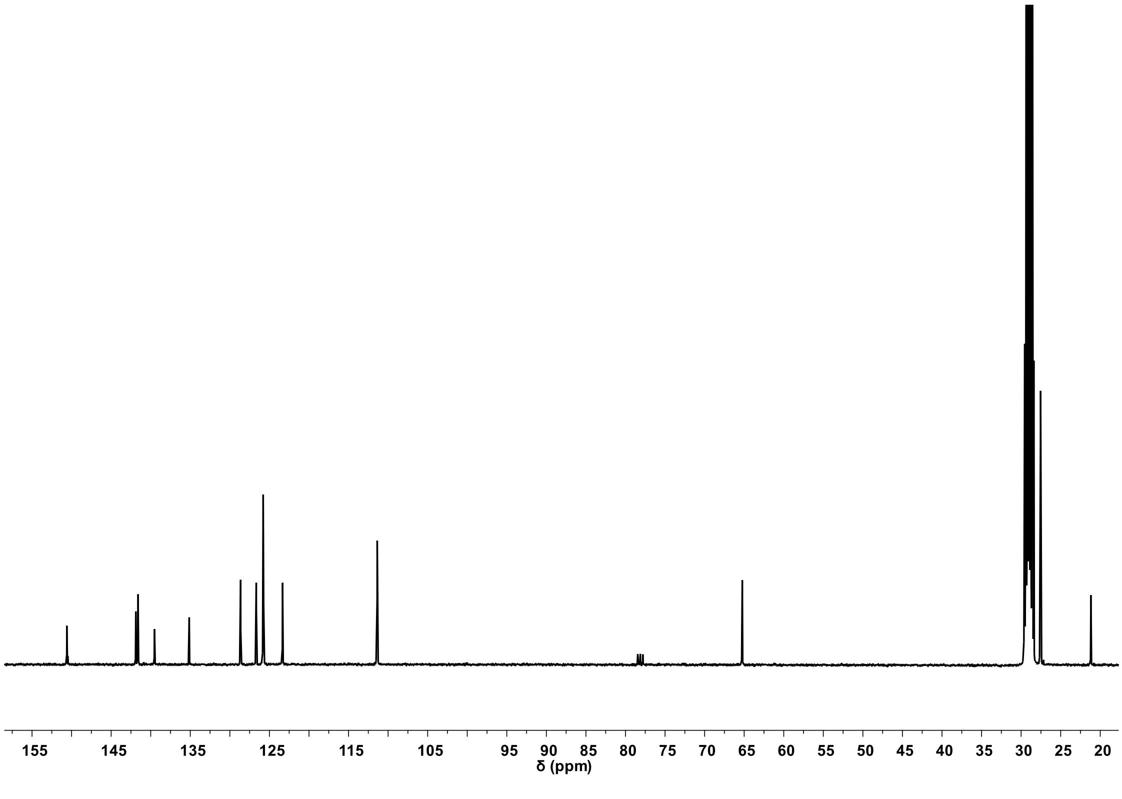

Figure S7. ^{13}C-NMR (400 MHz, Acetone-D$_6$, 298 K) spectrum of **4**.

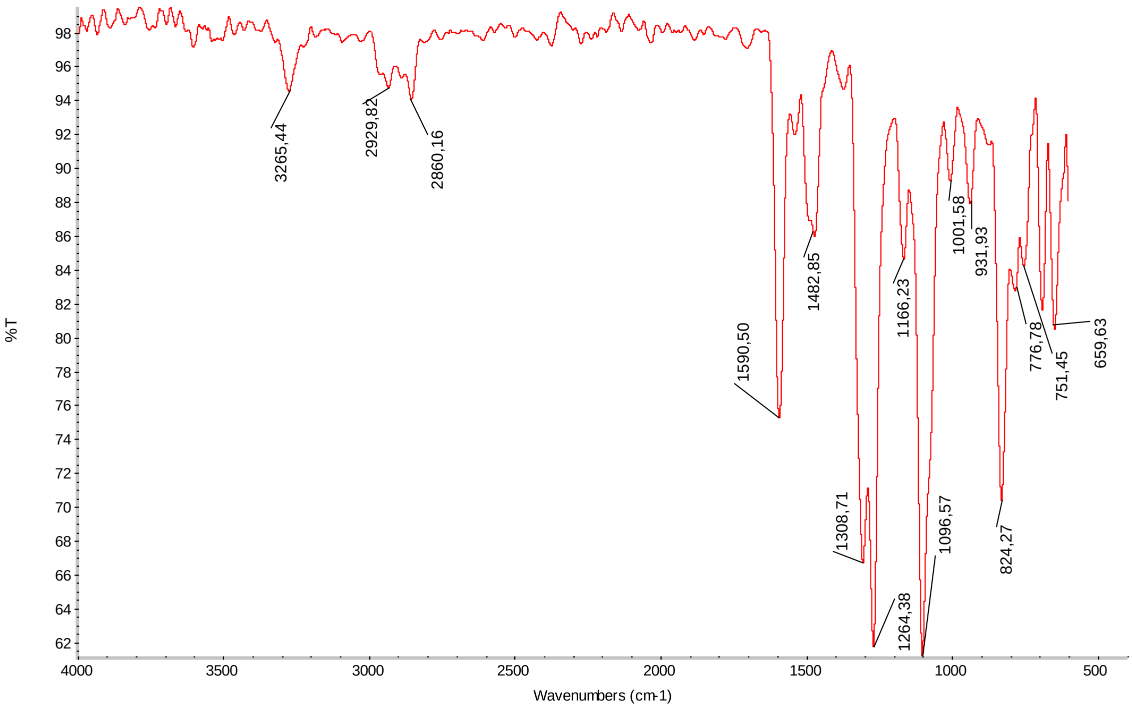

Figure S8. FT-IR (neat) spectrum of **4**.

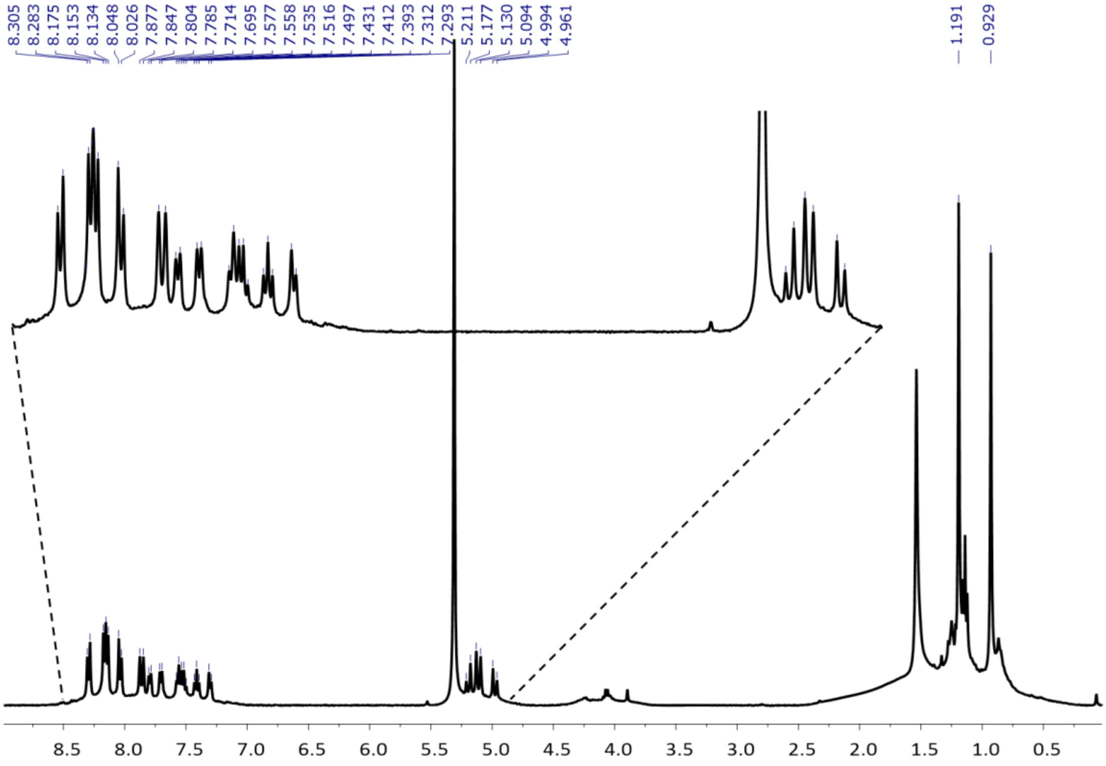

Figure S9. ^{1}H NMR (400 MHz, CD_2Cl_2, 298 K) spectrum of **5a**.

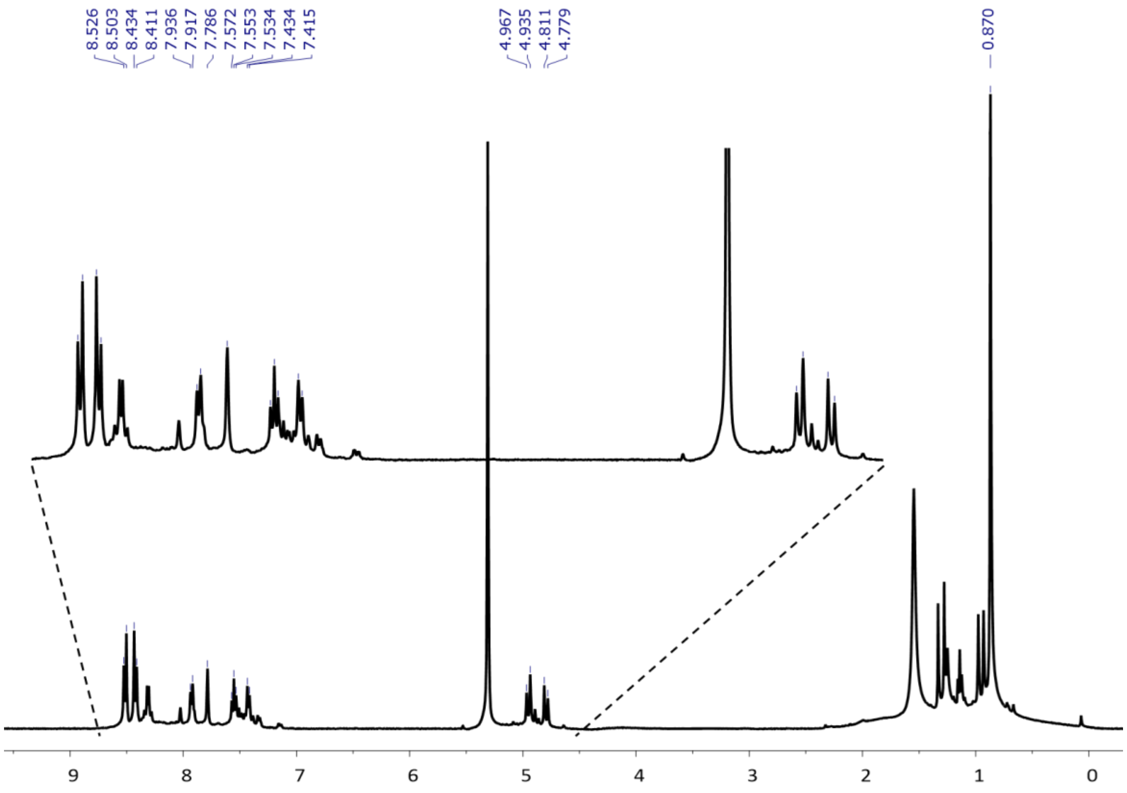

Figure S10.^{1}H NMR 400 MHz, (400 MHz, CD_2Cl_2, 298 K) spectrum of the mixture **5b** + **5c**.

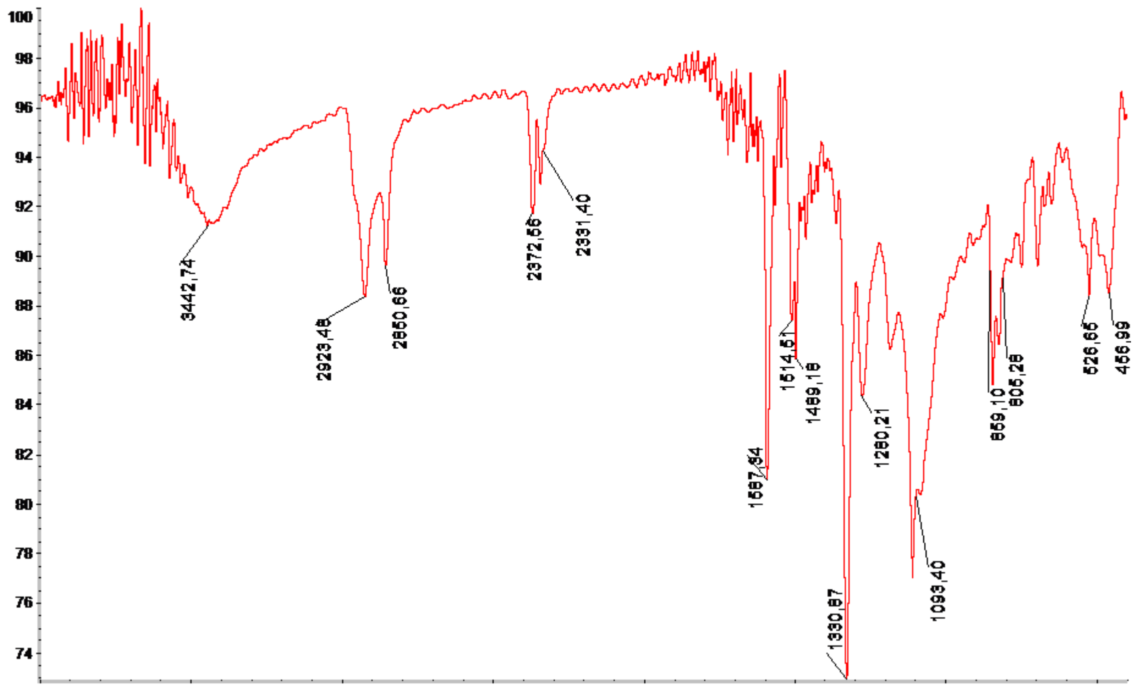

Figure S11. FT-IR (KBr) spectrum of **5**.

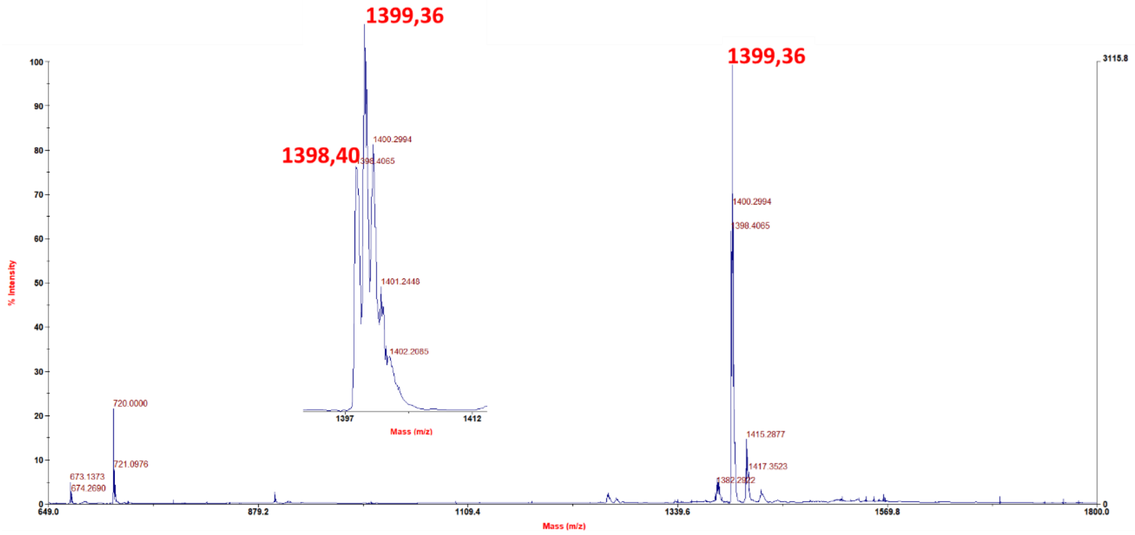

Figure S12. MS (MALDI-TOF) spectrum of **5.**

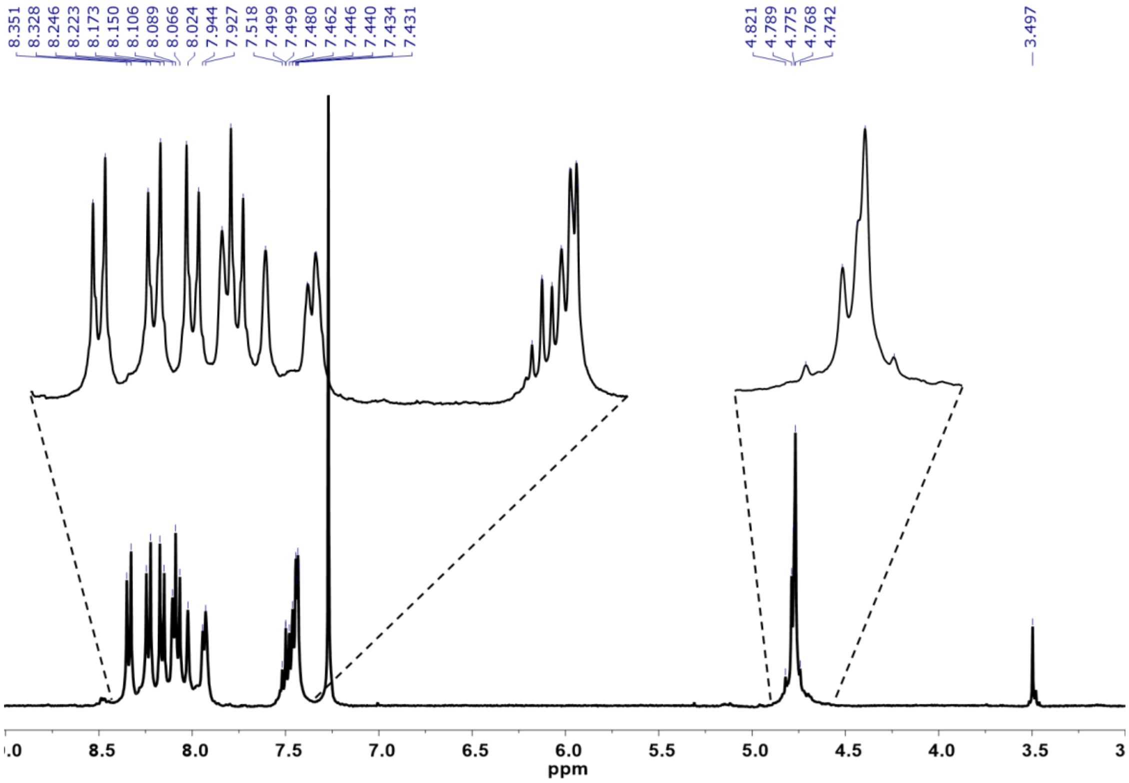

Figure S13. ^{1}H-NMR (400 MHz, $CDCl_3$, 298 K) spectrum of **6**

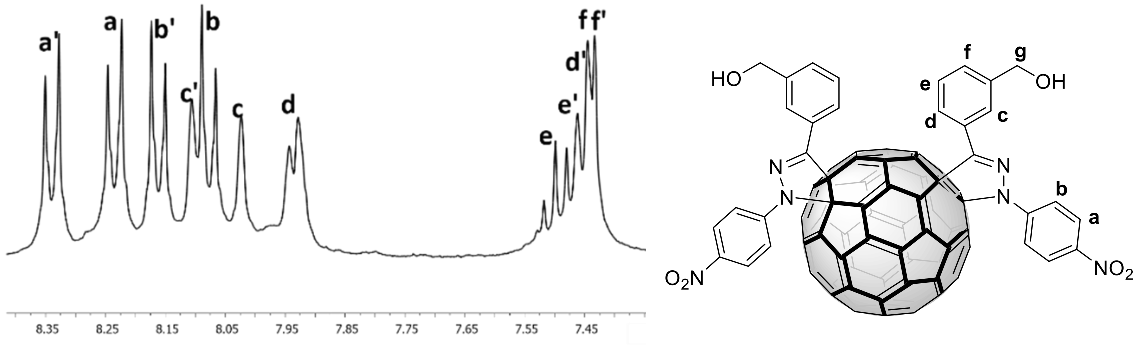

Figure S14. Aromatic region of the ^{1}H NMR spectrum of **6** (solvent CS_2:$CDCl_3$).

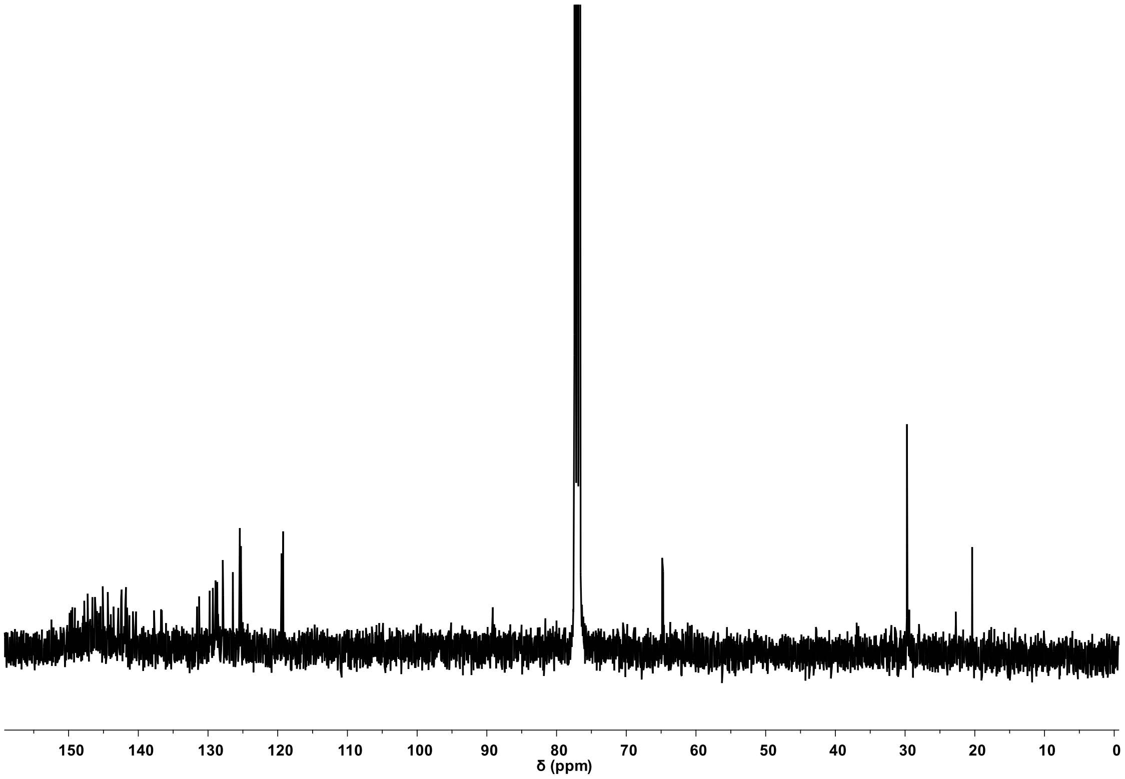

Figure S15. ^{13}C-NMR (400 MHz, $CDCl_3$, 298 K) spectrum of **6**.

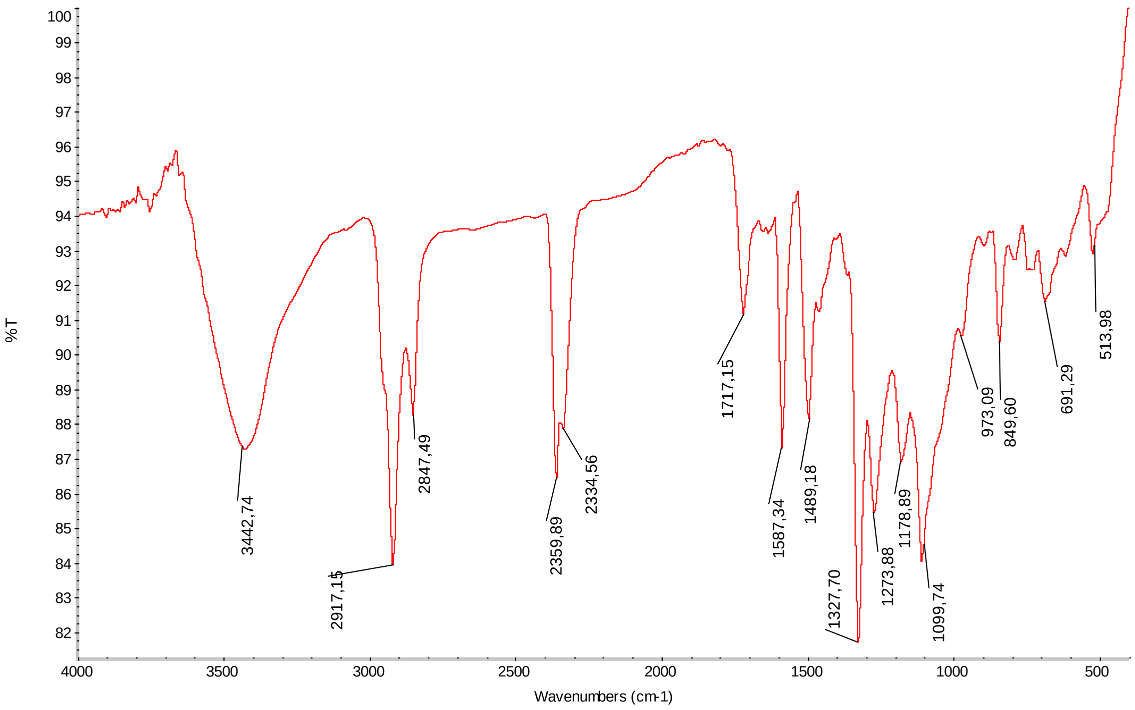

Figure S16. FT-IR (KBr) spectrum of **6**.

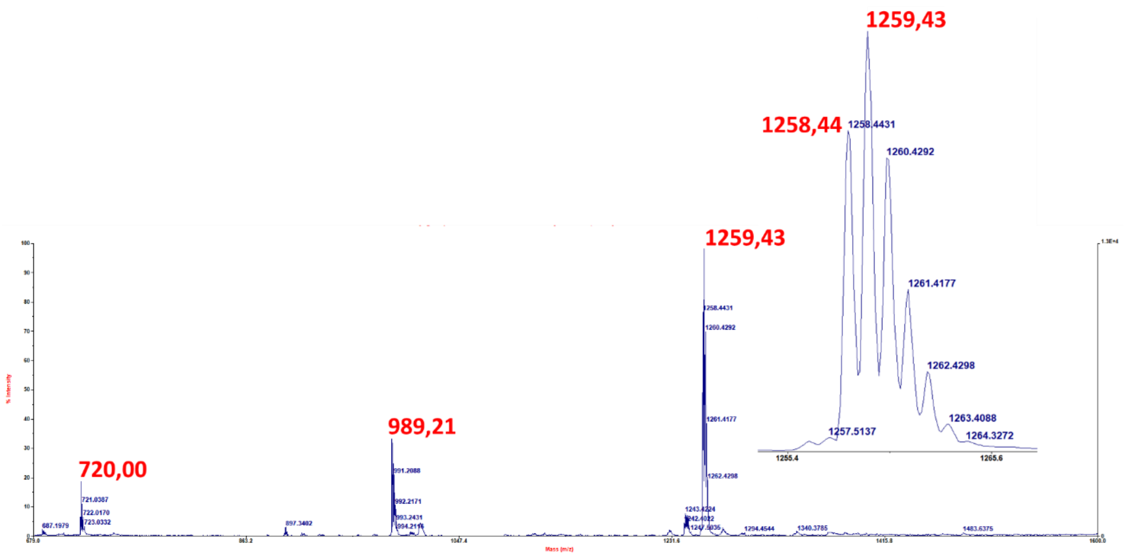

Figure S17. MS (MALDI-TOF) spectrum of **6**.

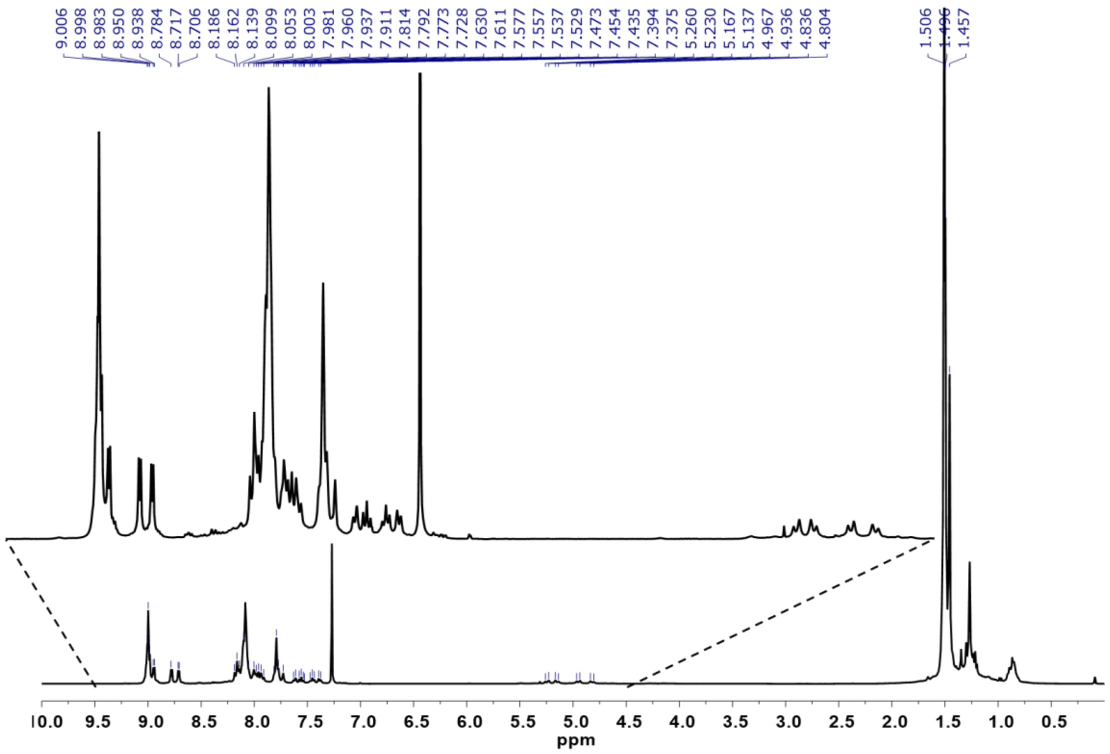

Figure S18. ^{1}H-NMR (400 MHz, $CDCl_3$, 298 K) spectrum of **1**.

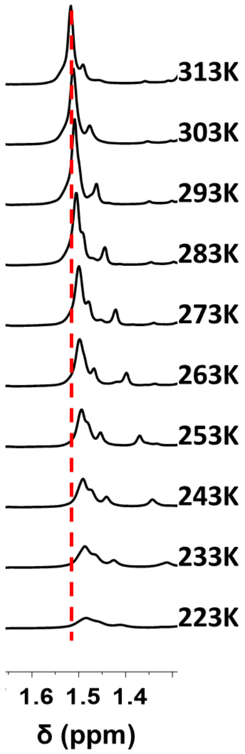

Figure S19. Part of the ^{1}H-NMR spectra recorded for **1** at different temperatures (1.5 mM $CDCl_3$, 400 MHz).

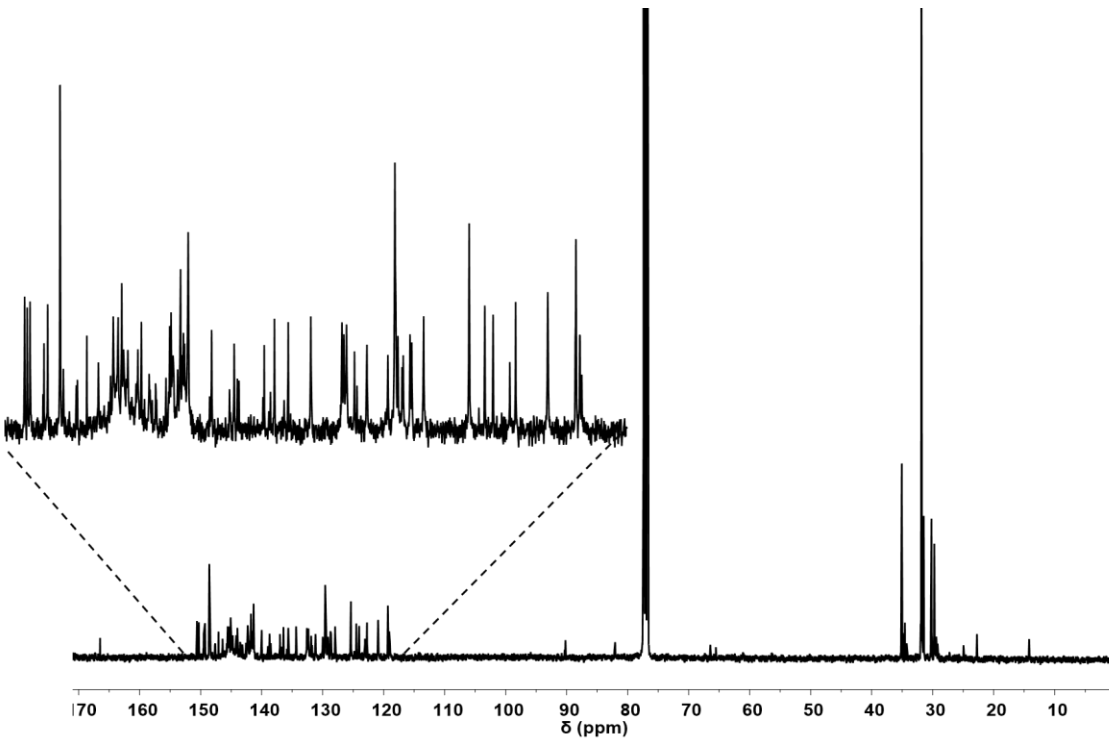

Figure S20. ^{13}C-NMR (400 MHz, $CDCl_3$, 298 K) spectrum of **1**.

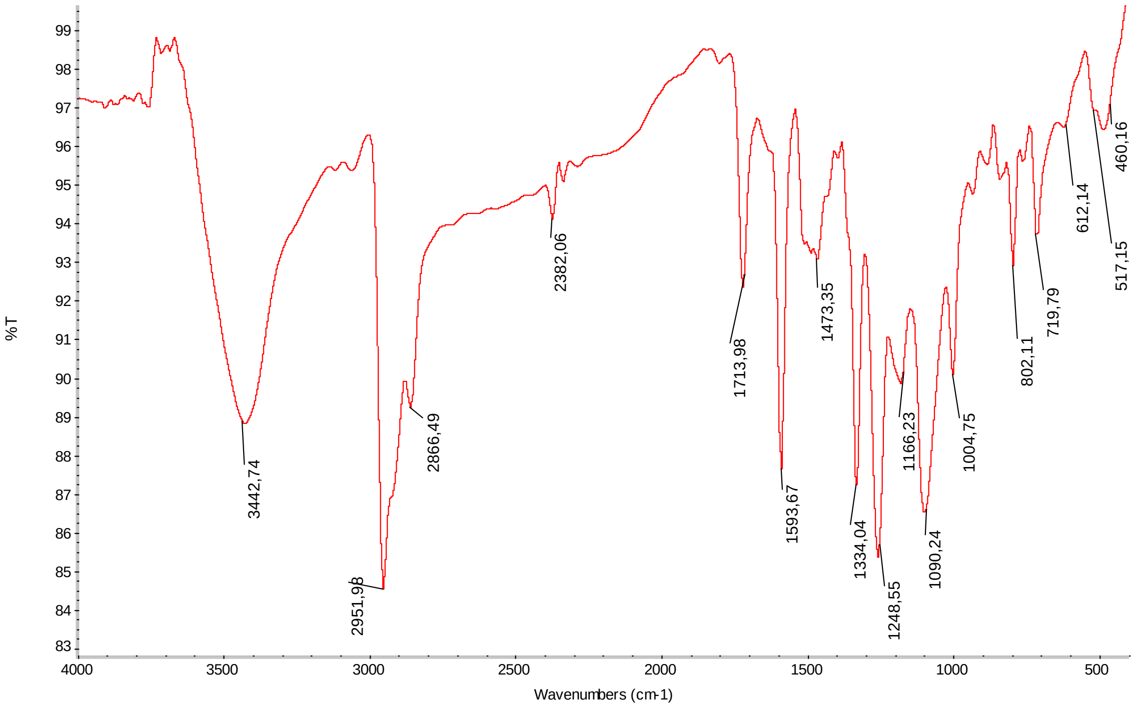

Figure S21. FT-IR (KBr) spectrum of **1**.

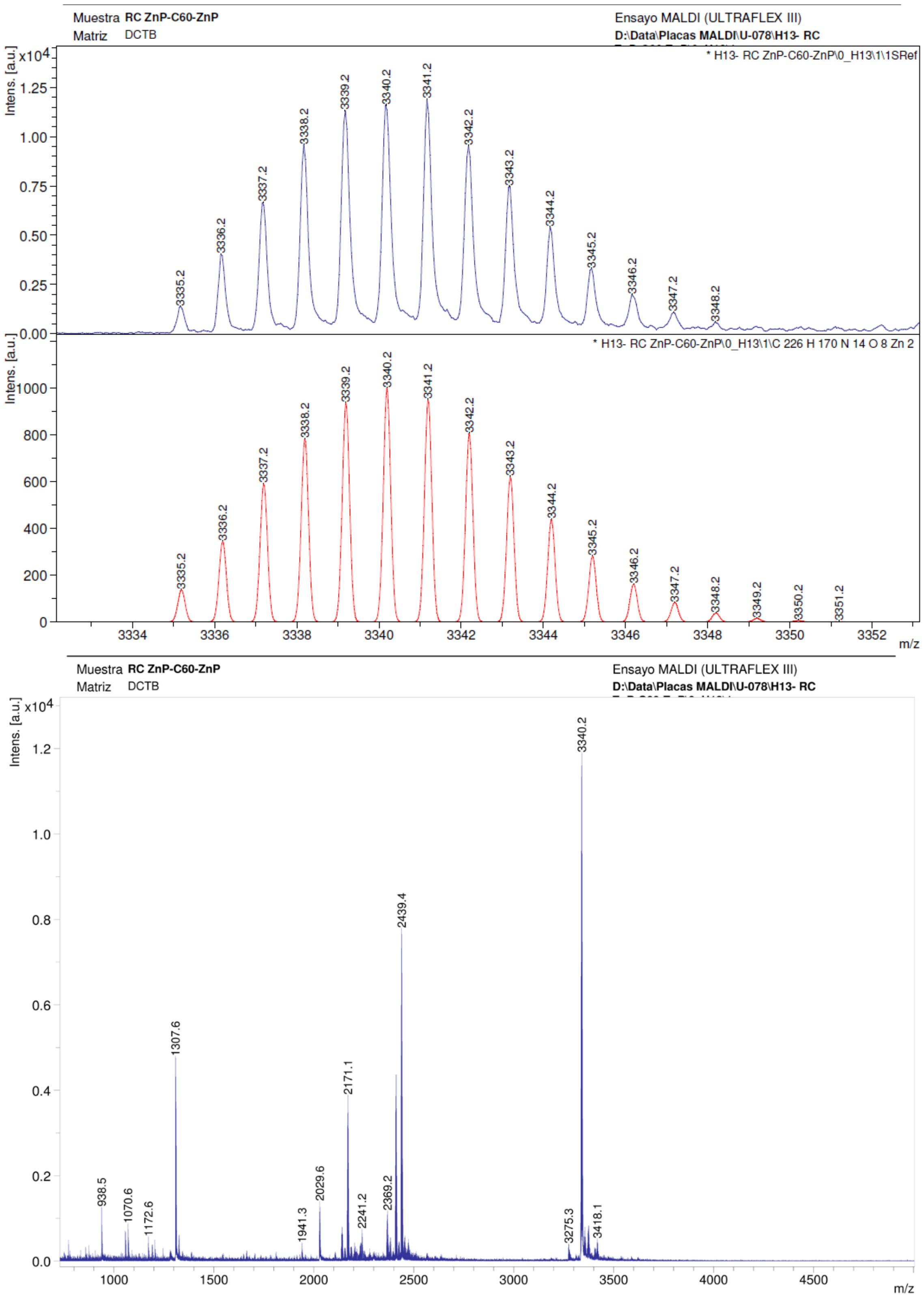

Figure S22. MS (MALDI-TOF) spectrum of **1**.

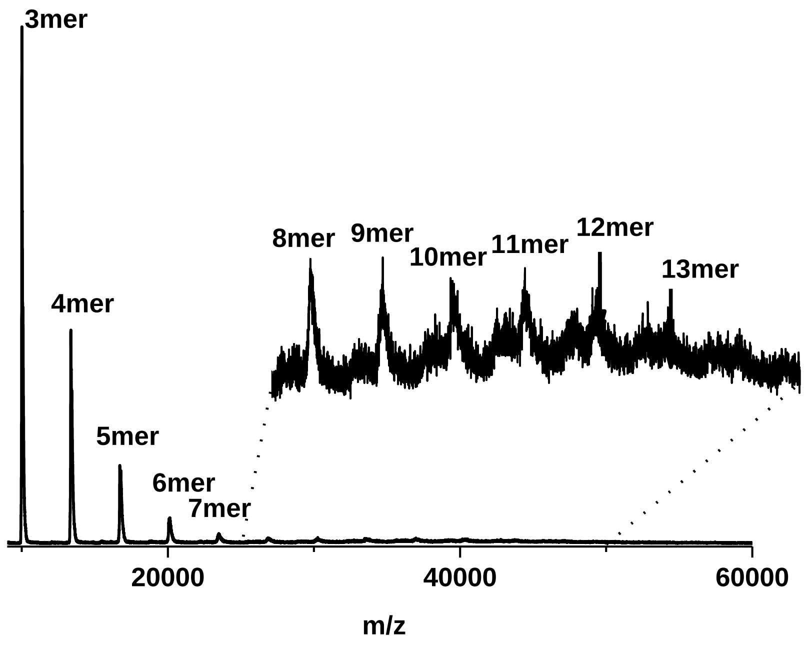

Figure S23. High m/z zoom of the MS (MALDI-TOF) spectrum of **1**.

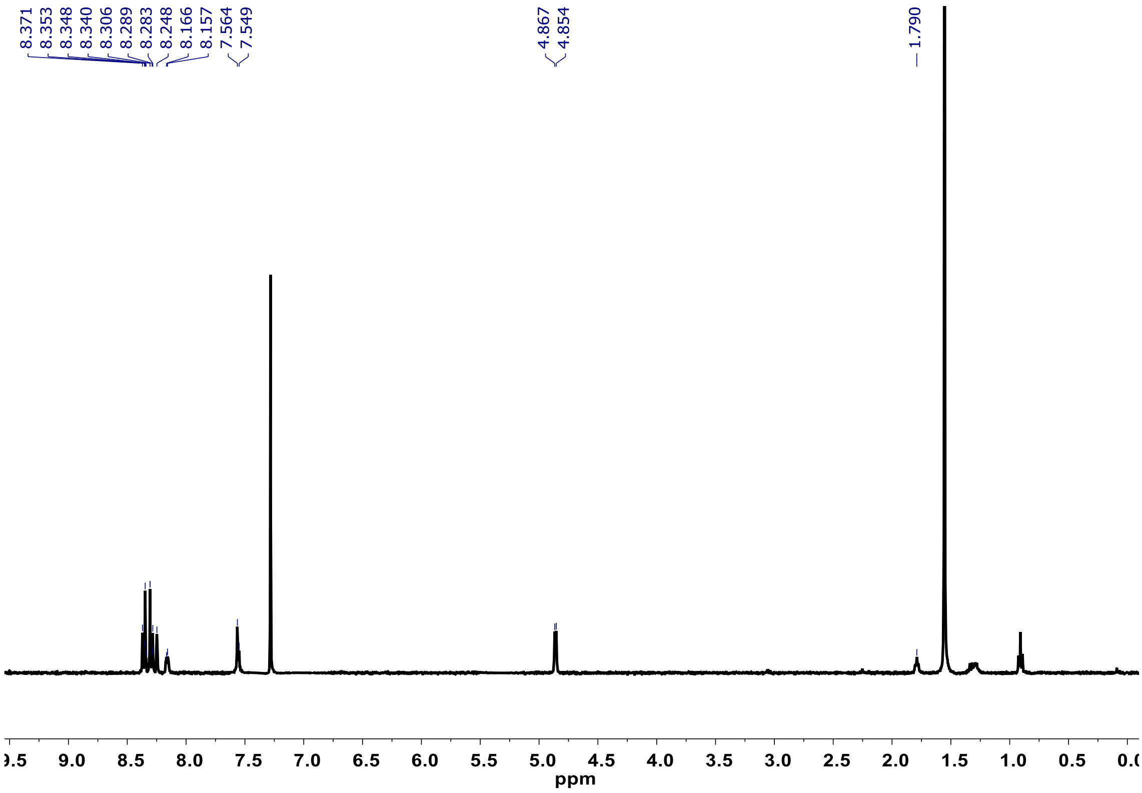

Figure S24. ^{1}H-NMR (400 MHz, $CDCl_3$, 298 K) spectrum of **9**.

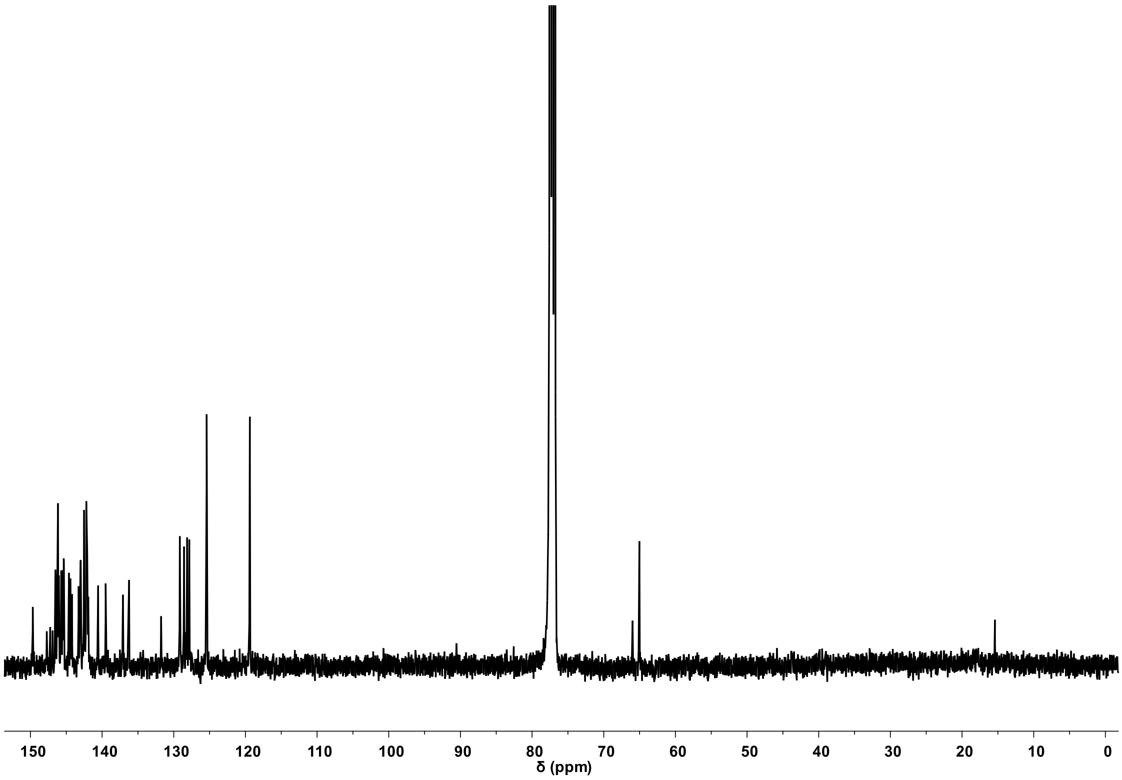

Figure S25. ^{13}C-NMR (400 MHz, $CDCl_3$, 298 K) spectrum of **9**.

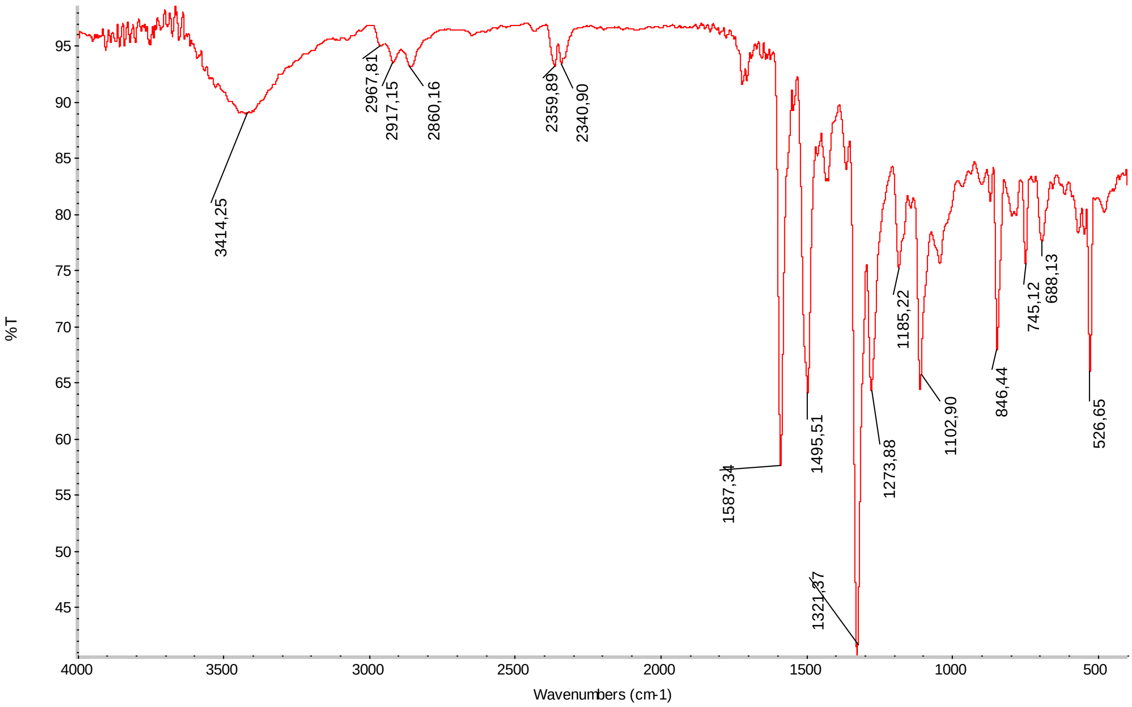

Figure S26. FT-IR (KBr) spectrum of **9**.

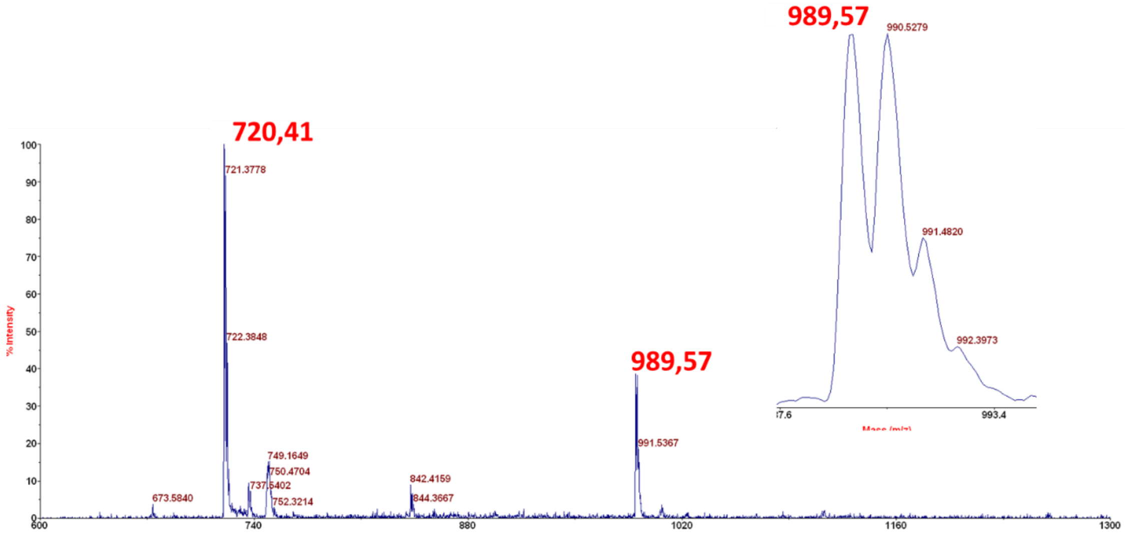

Figure S27. MS (MALDI-TOF) spectrum of **9**.

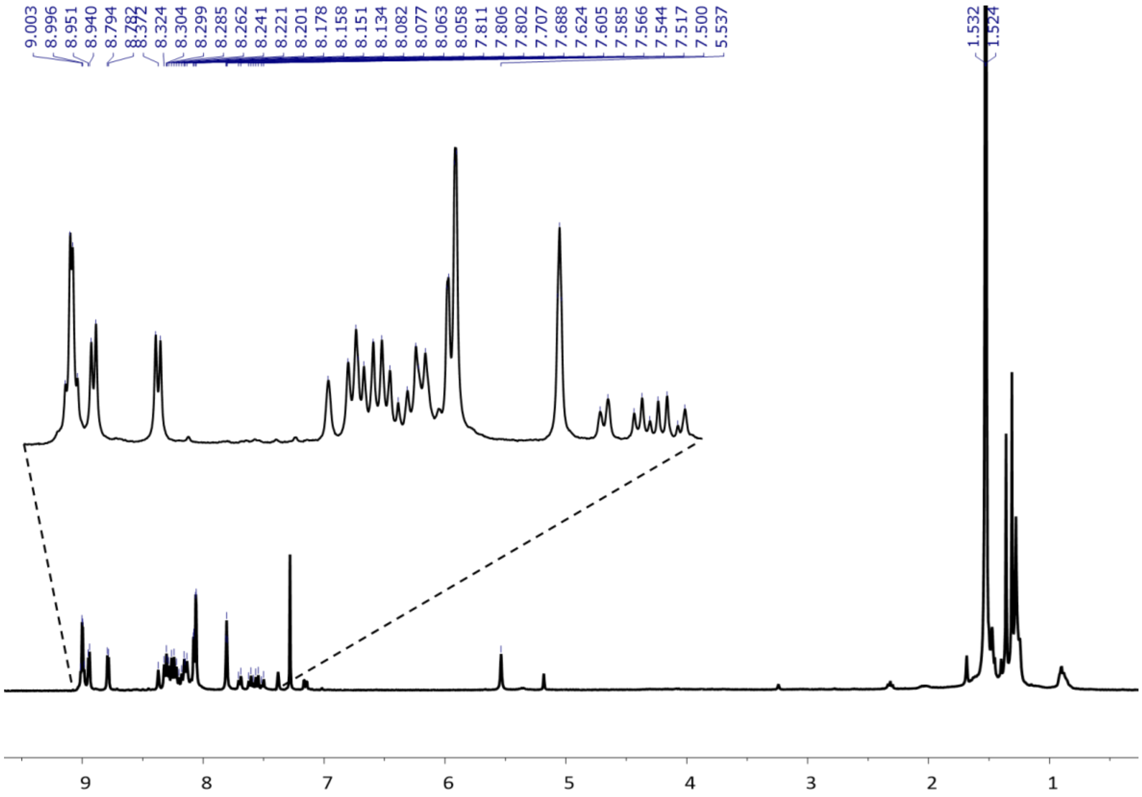

Figure S28. ^{1}H-NMR (400 MHz, $CDCl_3$, 298 K) spectrum of **2**.

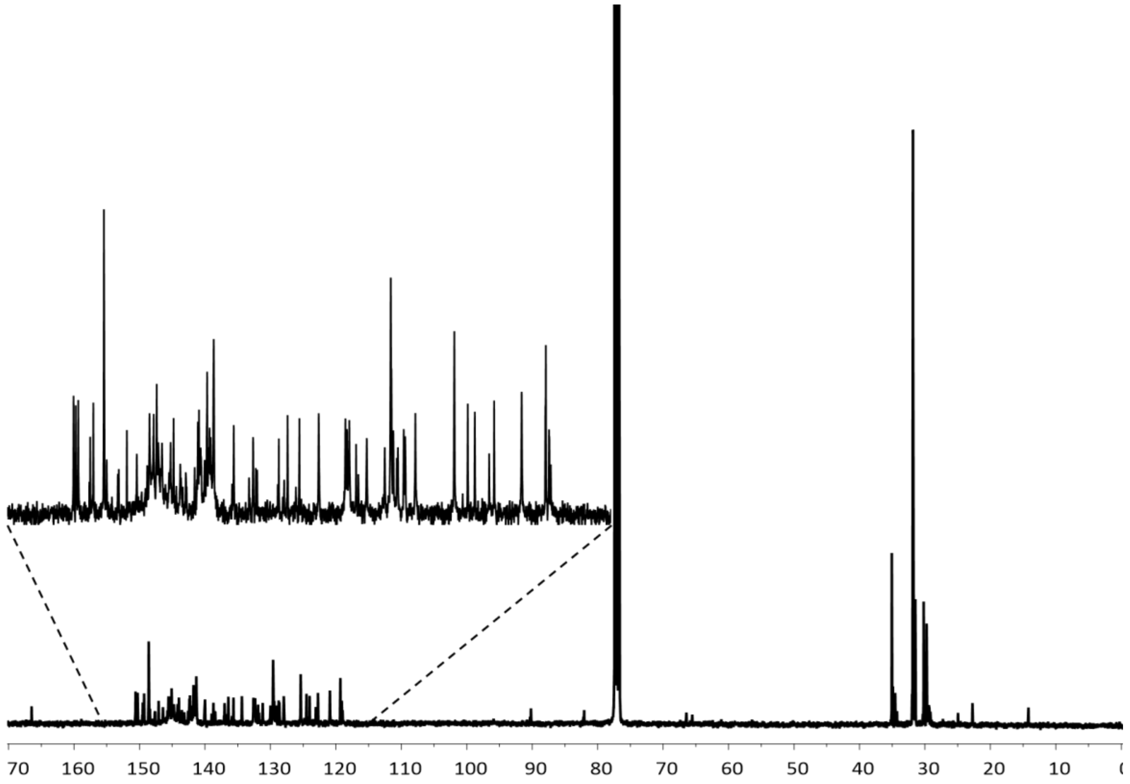

Figure S29. ^{13}C-NMR (400 MHz, $CDCl_3$, 298 K) spectrum of **2**.

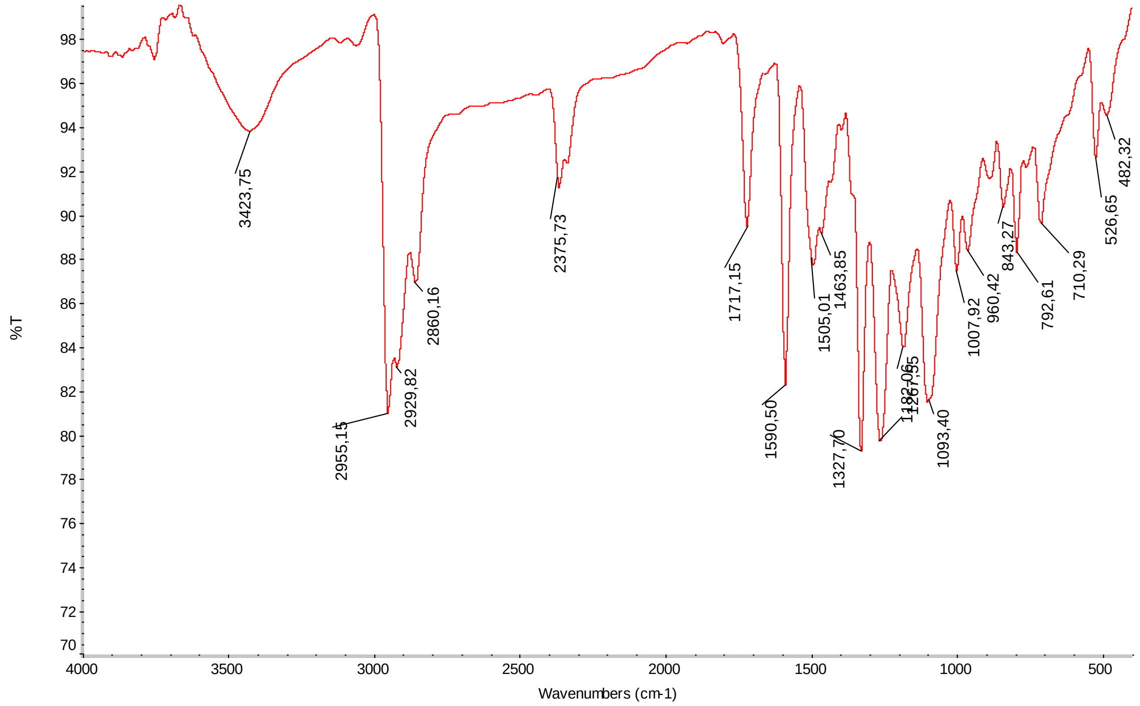

Figure S30. FT-IR (KBr) spectrum of **2**.

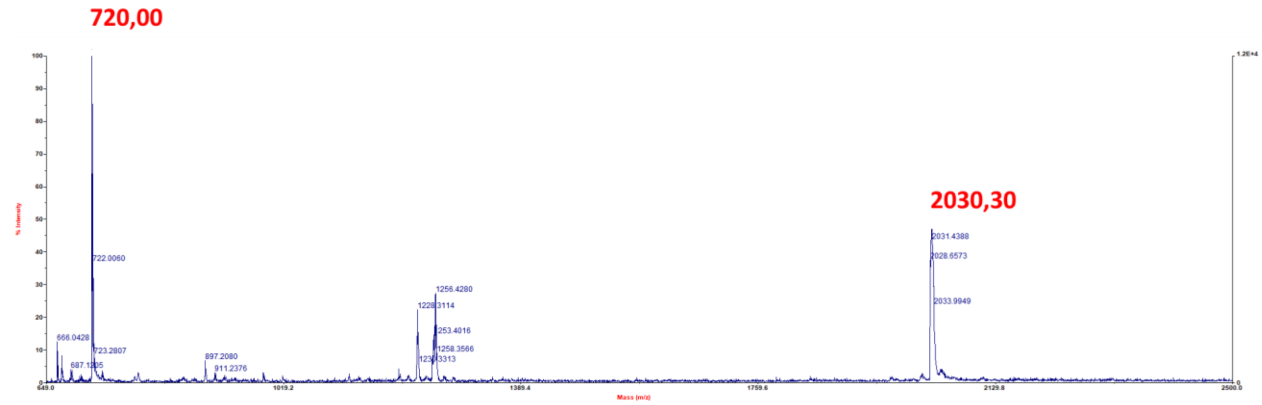

Figure S31. MS (MALDI-TOF) spectrum of **2**.

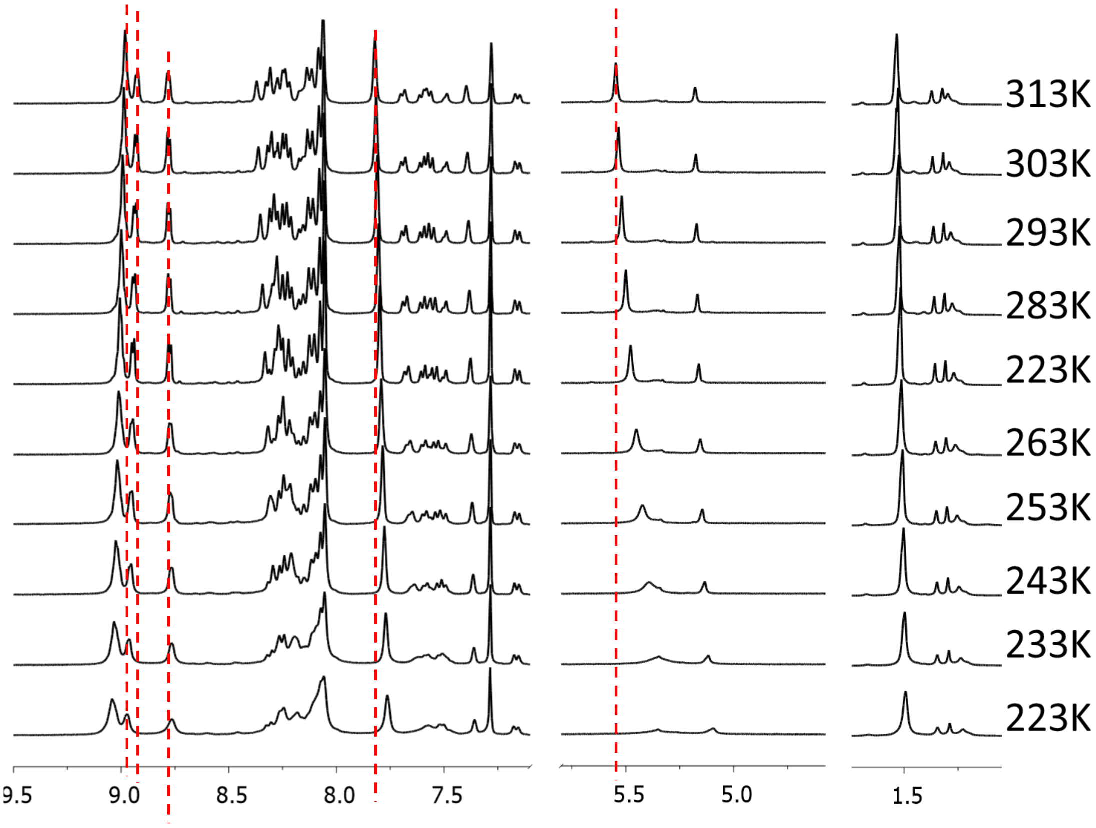

Figure S32. ^{1}H-NMR spectra of compound **2** at different temperatures (3.5 mM $CDCl_3$, 400 MHz)

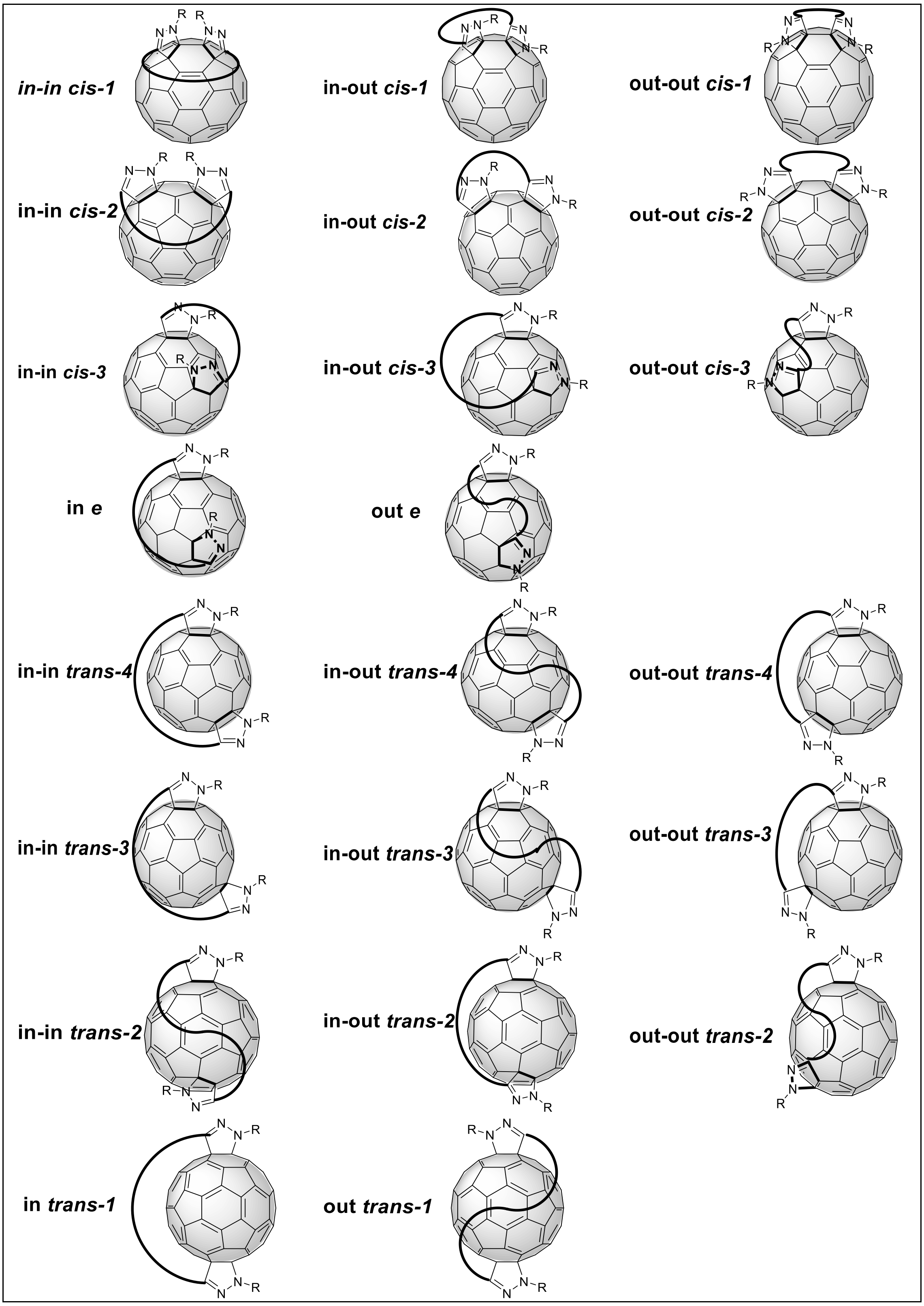

Figure S33. All possible isomers of bispyrazolino[60]fullerene.

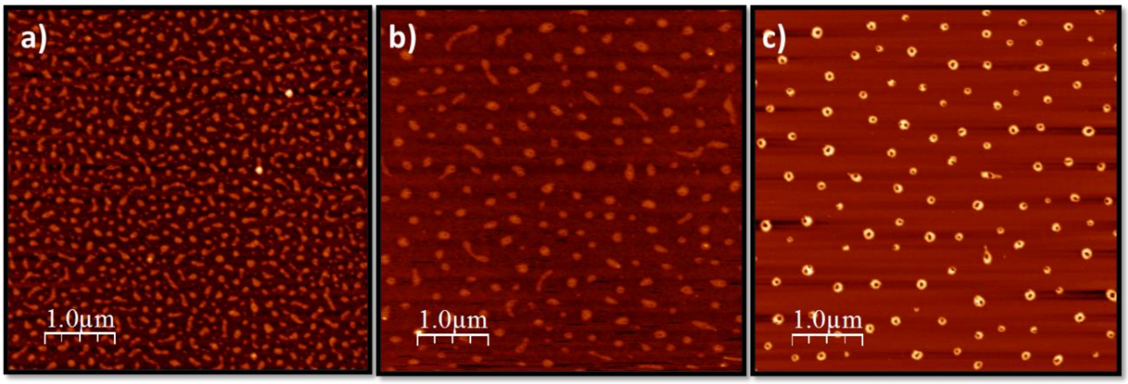

Figure S34. AFM follow-up study of the supramolecular aggregates of **1**: a) freshly prepared CH_3Cl solution spin-coated onto mica surface, b) spin-coated after 2 hours ,and c) spin-coated after 24 hours.

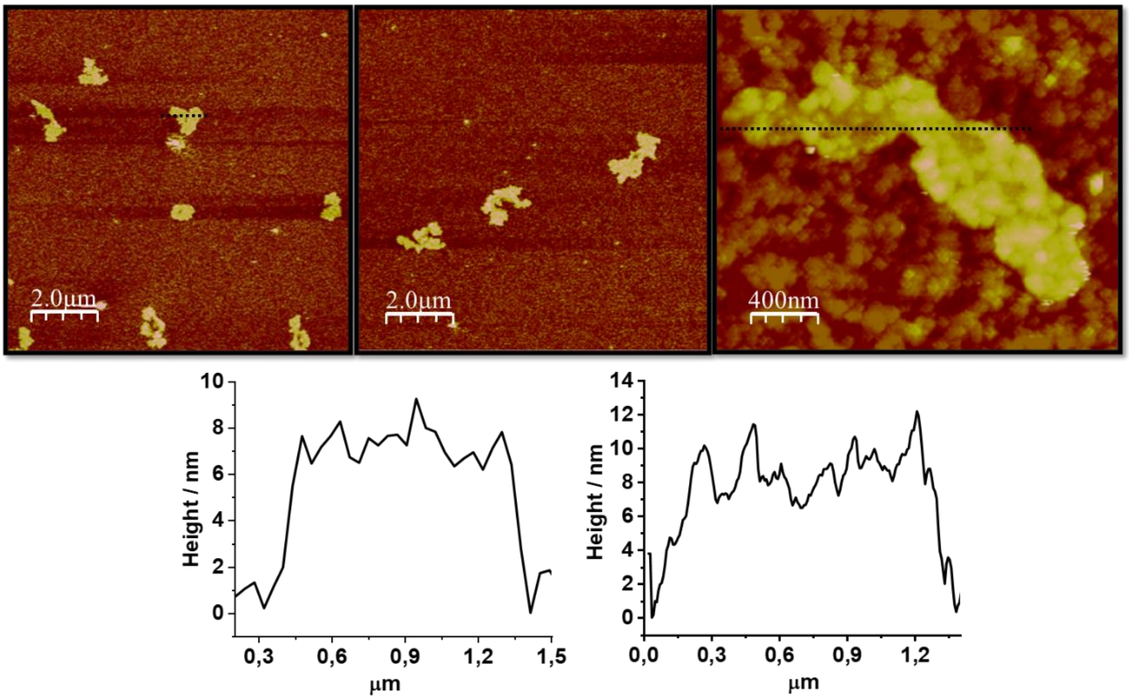

Figure S35. Top: AFM images corresponding to monoadduct **2** aggregates at different magnification. Bottom: AFM height profiles corresponding to the black dotted lines.

Table S1. Relative energy difference and molecular symmetry of the most stable regioisomers of **5**.

Isomer	Relative energy (kcal mol^{-1})	Group of symmetry
**out *e***	0	C_1
**out-out *trans-3***	+5.72	C_2
**out-out *trans-4***	+6.55	C_s
**in-out *cis-3***	+9.78	C_s
**out-out *cis-3***	+16.65	C_2
**in-out *cis-2***	+25.40	C_1
**out out *cis-2***	+31.97	C_s
**in *e***	+49.13	C_1
**out-out *trans-2***	+52.87	C_2
**out-out *trans-4***	+65.99	C_s
**in-in *cis-2***	+85.09	C_s
**in-out *cis-3***	+112.05	C_1

IV. Theoretical calculations

Supramolecular aggregates of bisadduct **1**, (**1**)$_n$ with n = 2, 3, 4, 5, 6, 8, 10, 12, 14, 16, and 18, were first fully optimized at the molecular mechanics (MM) level by using the generic GFN-FF force field recently developed by Spicher and Grimme.[4] Linear and curved structures were considered for each oligomer as shown in Figure S36 for n = 6, 14, and 18 and in Figure 4 in the main text for n = 10. Curved structures were computed to be more stable than linear structures by 2.6 kcal mol^{-1} per monomeric unit. This energy difference remains constant from the hexamer, due to the reduced relative weight of the geometrical effects associated to the terminal units of the oligomer. The intermediate (**1**)$_9$ oligomer, which bears 3780 atoms, was also optimized at the more accurate semiempirical GFN2-xTB level (Figure S3) using the Grimmes xTB program.[5] The GFN2-xTB method is based on a Hamiltonian similar to the well-known DFTB3,[6] uses a minimal valence basis set centered on atoms (STO-mG), and includes the latest density-dependent D4 dispersion correction. The structural parameters calculated for (**1**)$_9$ at the GFN2-xTB level are similar to those obtained at the MM/GFN-FF level. For the curved aggregate, that is more stable than the linear structure by 0.73 kcal/mol per monomeric unit, the central three fullerenes are separated by 21.50 Å and form an angle of 174° compared with the values of 21.12 Å and 171° obtained, respectively, at the MM/GFN-FF level. The distances between the two Zn atoms and the mot external *tert*-butyl hydrogens are 9.35 and 26.06 Å, respectively, for the curved aggregate, that compare well with the MM/GFN-FF values of 9.44 and 25.76 Å, respectively.

The electronic properties of compounds **1** and **2** along with their disubstituted C_{60} (**6**) and ZnP-COOH (**7**) molecular fragments were computed at the density functional theory (DFT) B3LYP/6-31G** level,[7] using the A03 revision of the Gaussian 16 package.[8] The molecular structures of **6**, **7**, monoadduct **2**, and bisadduct **1**, together with those of the **6**$^{\bullet-}$ and **7**$^{\bullet+}$ radical species, were optimized in vacuum using B3LYP/6-31G**. To investigate the effect on the electronic properties of the supramolecular ZnP···C_{60} interactions taking place in the aggregates of bisadduct **1**, the structure of a **7**···**6** dimer extracted from the (**1**)$_{10}$ aggregate was reoptimized at the B3LYP-D3/6-31G** level including the D3 dispersion term.[9] Single-point calculations on neutral, cation, and anion optimized species were performed in o-dichlorobenzene within the polarizable continuum model (PCM) approach[10] for better comparison with the experimental electrochemical properties. Excited-state calculations were performed on the optimized geometries using the time-dependent density functional theory (TDDFT) at the B3LYP/6-31G** level with benzonitrile as solvent.

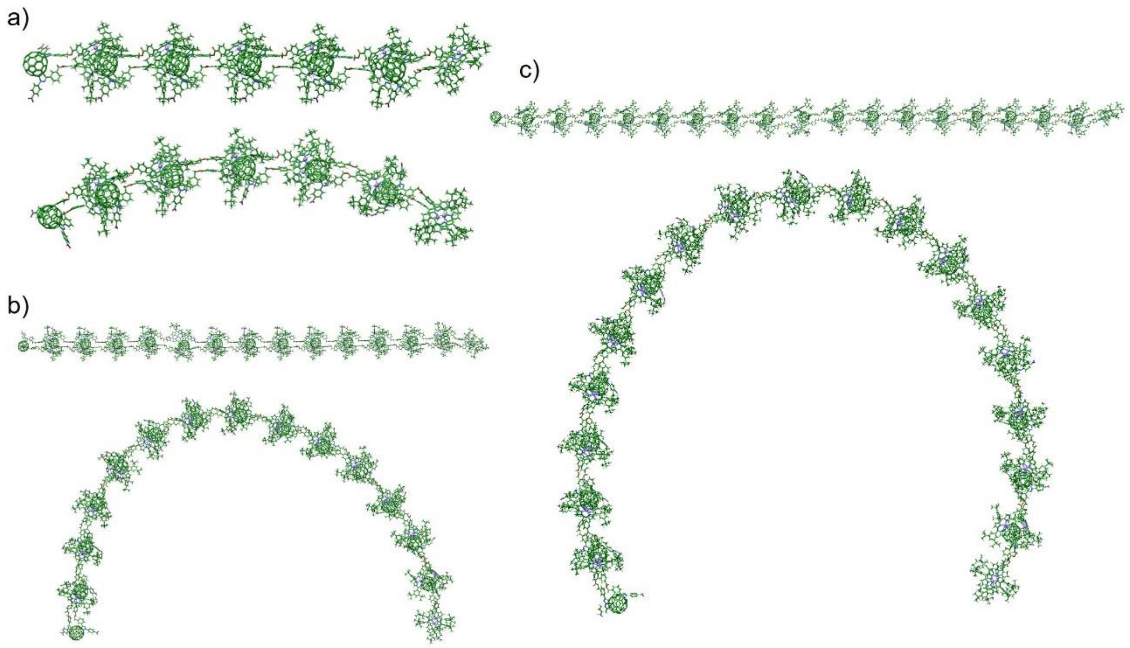

Figure S36. GFN-FF-optimized structures calculated for linear and curved aggregates of bisadduct **1** incorporating 6 (a), 14 (b), and 18 (c) monomer units.

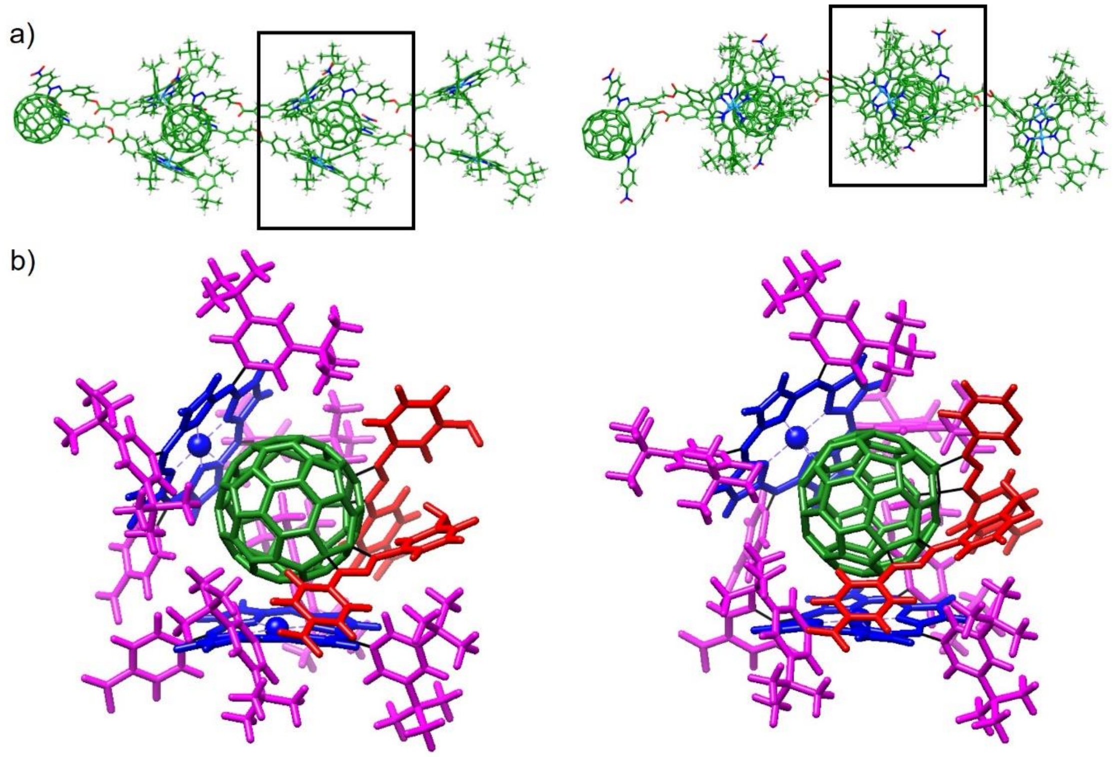

Figure S37. (a) Central trimer of the linear (left) and curved (right) structure optimized at the GFN-xTB level for a nonamer aggregate of bisadduct **1**. (b) Zoomed view of the interaction center (square in (a)) for linear (left) and curved (right) aggregates. C_{60} fragment in green, C_{60} substituents in red, porphyrin ring in blue, and porphyrin di-tert-butylphenyl meso substituents in magenta.

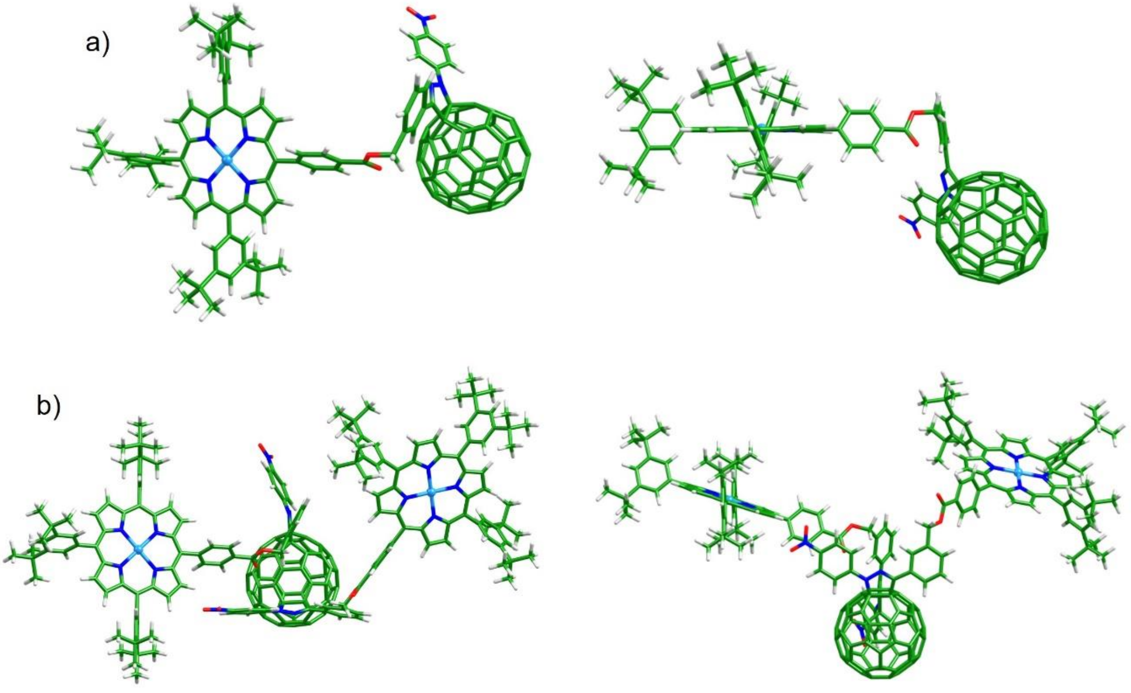

Figure S38. B3LYP-6-31G**-optimized structures calculated for (a) monoadduct **2** and (b) bisadduct **1**. Two different perspectives are shown for each molecule.

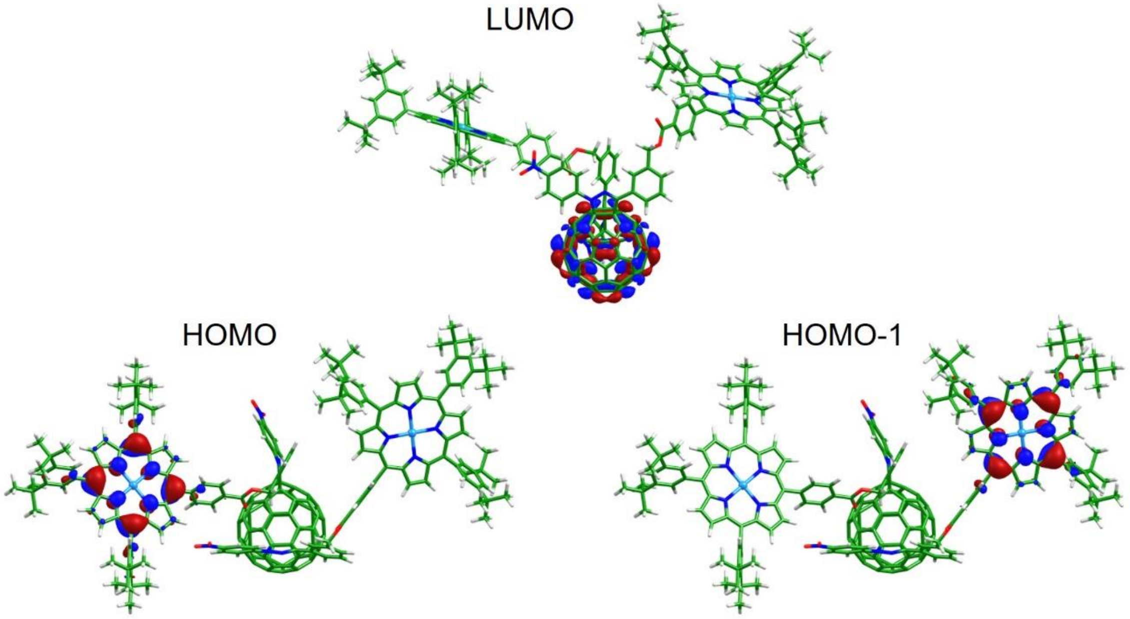

Figure S39. Isovalue contour plots (±0.03 a.u.) calculated at the B3LYP-6-31G** level for the HOMO-1, HOMO, and LUMO of bisadduct **1**.

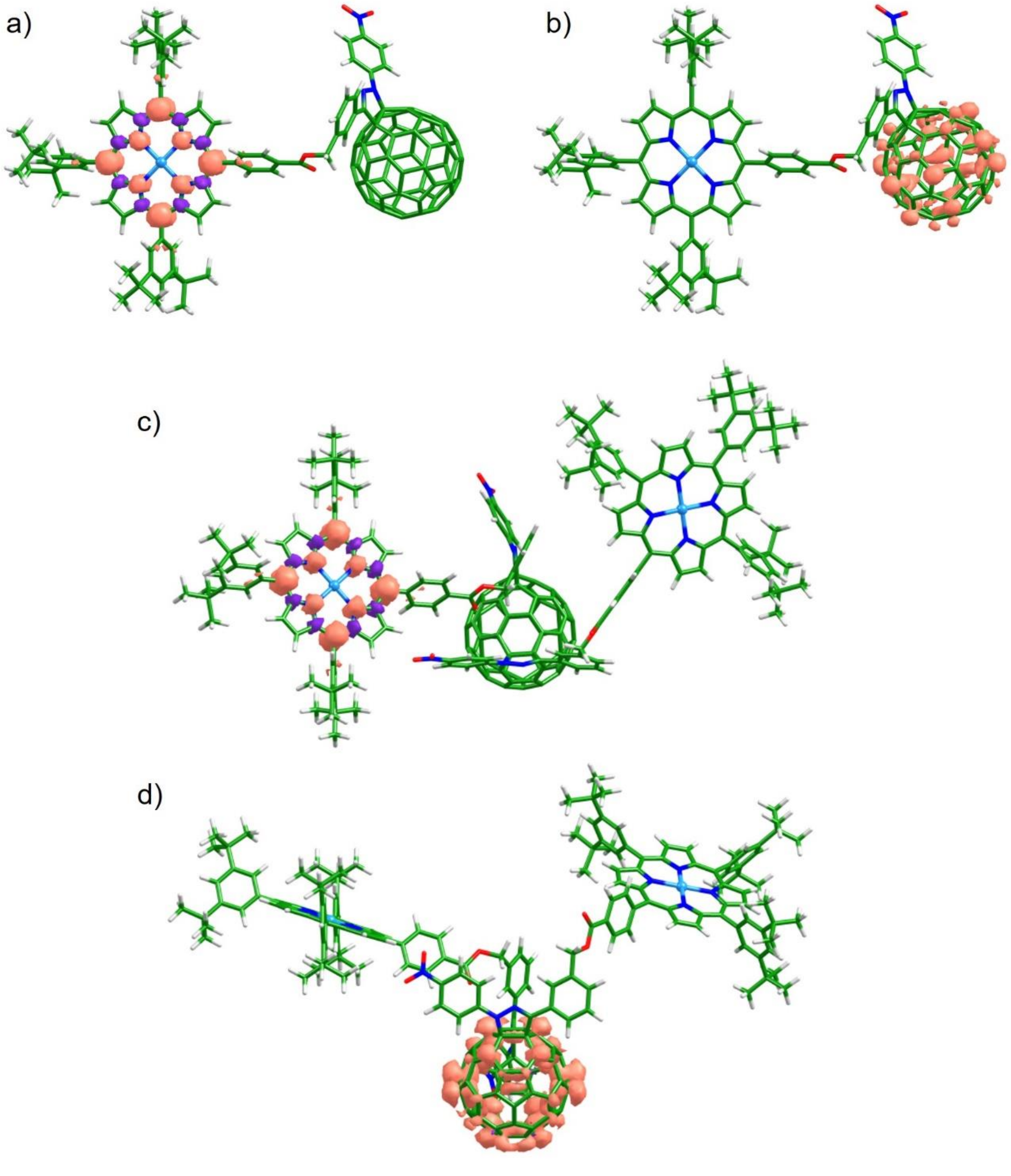

Figure S40. Unpaired-electron spin-density plots (isocontours of 0.002 a.u.) computed at the spin-unrestricted UB3LYP-6-31G** level for the cation (a and c) and anion (b and d) of monoadduct **2** (a, b) and bisadduct **1** (c, d).

Table S2. Low-lying singlet excited states calculated at the TD-DFT B3LYP/6-31G** level for monoadduct **2**. Vertical excitation energies (*E*), oscillator strengths (*f*), dominant monoexcitations with contributions (within parentheses) greater than 20%, and description of the excited state (CT: charge-transfer state, ZnP*: excited state localized over the ZnP moiety, C_{60}*: excited state localized over C_{60}. H and L denote HOMO and LUMO, respectively.

S_n	*E* (eV/nm)	*f*	Monoexcitations (%)	Description
1	1.76 / 703	0.000	H⟶L (99)	CT
2	1.83 / 676	0.000	H⟶L+1 (99)	CT
3	1.96 / 633	0.002	H-3⟶L (89)	C_{60}*
4	1.97 / 631	0.000	H-1⟶L (99)	CT
5	2.01 / 617	0.001	H-2⟶L (58)<br>H-5⟶L (21)	C_{60}*
6	2.03 / 610	0.000	H-3⟶L (75)	C_{60}*
7	2.04 / 609	0.000	H-1⟶L+1 (99)	CT
8	2.08 / 597	0.000	H-4⟶L (96)	C_{60}*
9	2.08 / 595	0.000	H⟶L+2 (99)	CT
10	2.11 / 586	0.005	H-2⟶L+1 (82)	C_{60}*
15	2.28 / 543	0.060	H⟶L+4 (64)<br>H-1⟶L+5 (35)	ZnP*
16	2.29 / 542	0.000	H-1⟶L+2 (99)	CT
17	2.30 / 540	0.022	H⟶L+5 (59)<br>H-1⟶L+4 (40)	ZnP*
34	3.02 / 410	1.280	H-1⟶L+5 (40)<br>H⟶L+4 (21)<br>H⟶L+8 (21)	ZnP*
36	3.05 / 407	1.571	H-1⟶L+4 (75)	ZnP*

Table S3. Low-lying singlet excited states calculated at the TD-DFT B3LYP/6-31G** level for the structural model of the intermolecular ZnP···C60 interaction taking place in the aggregates of bisadduct **1**. Vertical excitation energies (*E*), oscillator strengths (*f*), dominant monoexcitations with contributions (within parentheses) greater than 20%, and description of the excited state (ICT: intermolecular charge-transfer state, ZnP*: excited state localized over the ZnP moiety, C_{60}*: excited state localized over C_{60}. H and L denote HOMO and LUMO, respectively.

S_n	*E* (eV/nm)	*f*	Monoexcitations (%)	Description
1	1.53 / 812	0.027	H→L (98)	ICT
2	1.75 / 710	0.003	H→L+2 (88)	ICT
3	1.76 / 703	0.001	H-1→L (79)<br>H→L+1 (20)	ICT
4	1.77 / 699	0.028	H→L+1 (71)<br>H-1→L (20)	ICT
5	2.00 / 620	0.004	H-1→L+2 (38)<br>H-1→L+1 (36)	ICT
6	2.01 / 618	0.018	H-2→L (52)<br>H-1→L+2 (36)	C_{60}*/ICT
7	2.01 / 617	0.008	H-1→L+1(61)<br>H-1→L+2(23)	ICT/C_{60}*
8	2.08 / 595	0.001	H-4→L (64)	C_{60}*
9	2.14 / 579	0.001	H-5→L (63)	C_{60}*
10	2.21 / 561	0.001	H→L+3 (98)	C_{60}*
11	2.24 / 554	0.062	H→L+5 (61)<br>H-1→L+6 (27)	ZnP*
13	2.26 / 549	0.022	H→L+6 (59)<br>H-1→L+5 (35)	ZnP*
43	2.89 / 428	0.366	H-1→L+5 (16)	ZnP*
44	2.90 / 427	0.421	H-1→L+6 (22)	ZnP*

V.Electrochemical measurements

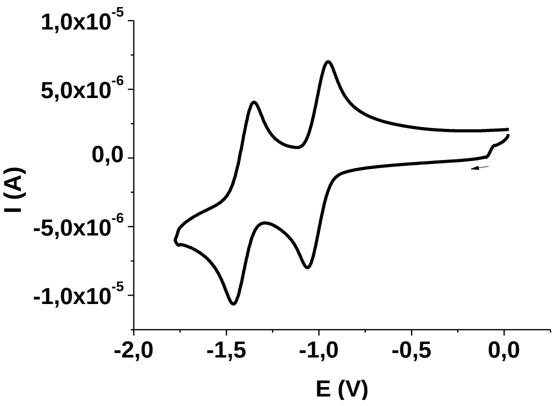

Figure S41. Cyclic voltammetry of C_{60}.

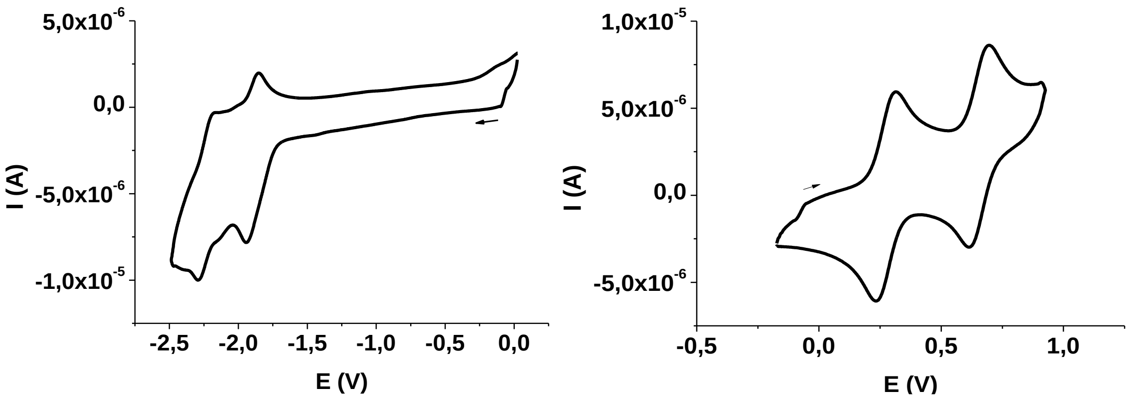

Figure S42. Cyclic voltammetry of compound **7**.

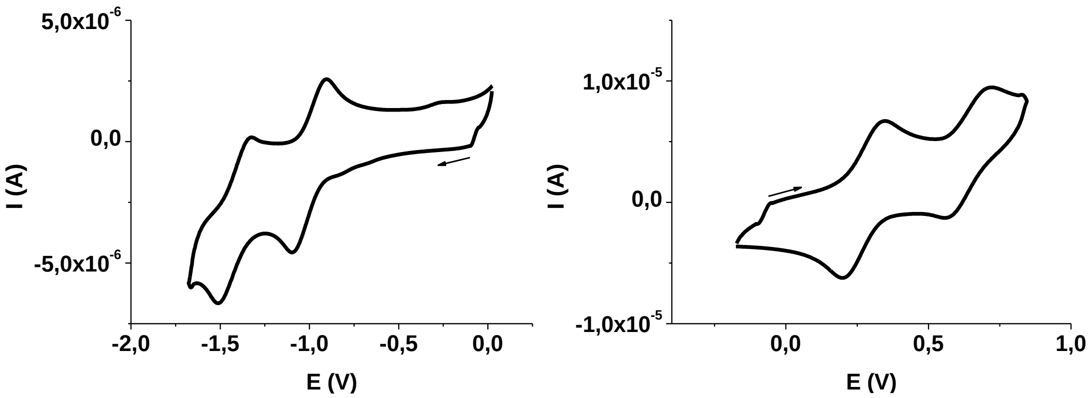

Figure S43. Cyclic voltammetry of compound **1**.

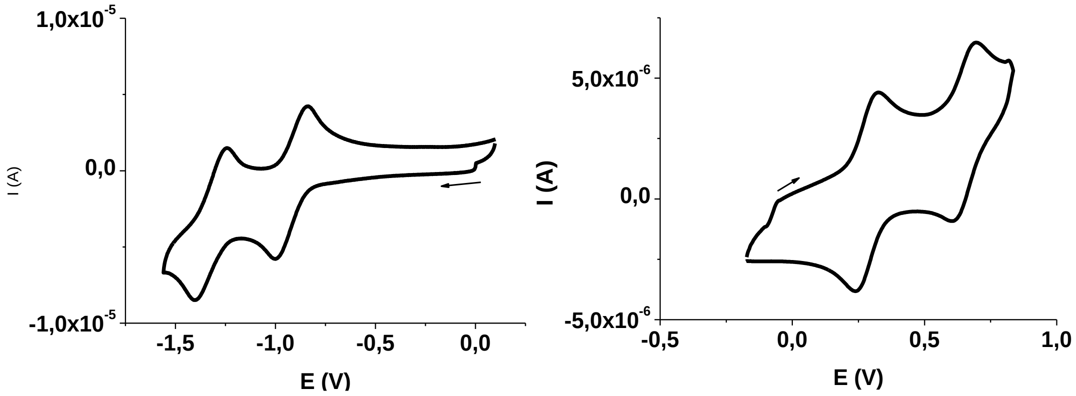

Figure S44. Cyclic voltammetry of compound **2**.

Table S4. Redox potentials (V vs. Fc/Fc^{+}) of the processes determined by OSWV for bisadduct **1,** monoadduct **2** and reference compounds. [a]

	E_1^{Red}	E_2^{Red}	E_1^{Ox}	E_2^{Ox}
**C_{60}**	-1.03	-1.42	-	-
**7**	-1.91	-2.17	+0.28	+0.66
**1**	-1.01	-1.41	+0.29	+0.66
**2**	-1.00	-1.40	+0.30	+0.67

[a] 0.5 mM in *o*-dichlorobenzene:acetonitrile (4:1), $Ag/AgNO_3$ (0.01 M) electrode was used as reference and checked against the ferrocene/ferrocenium couple (Fc/Fc^{+}), glassy carbon working electrode, Pt counter electrode, 298 K, 0.1 M Bu_4NClO_4, scan rate = 100 mV s^{-1}.

V. Photophysical measurements

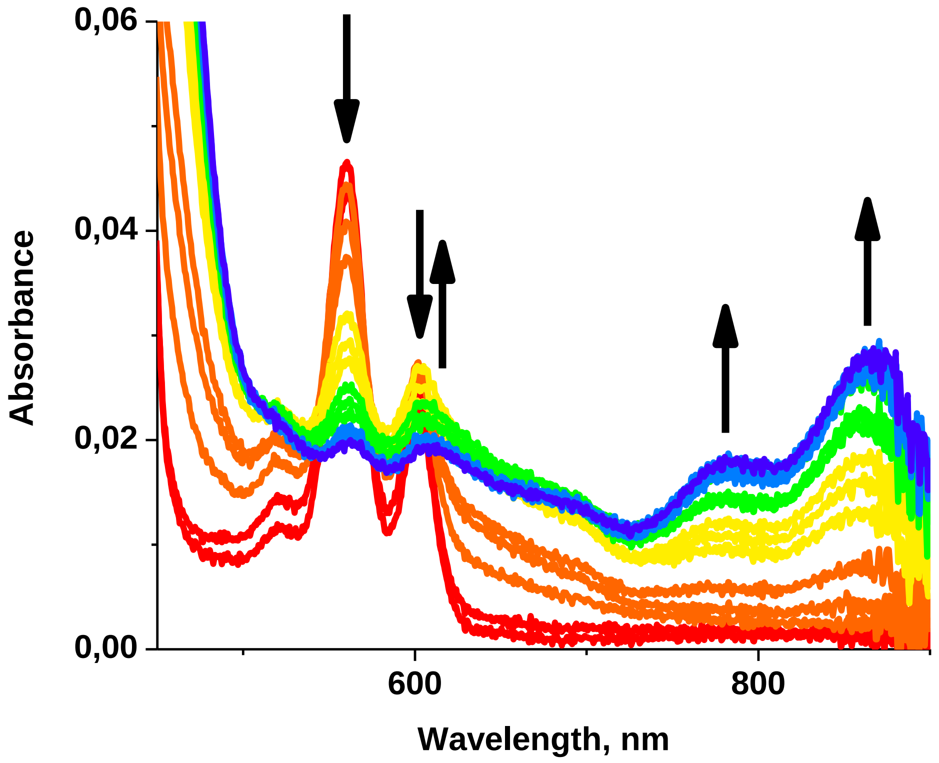

Figure S45. Spectral changes observed during increased addition of NOBF (in methanol) to ZnP-COOH (**7**) in benzonitrile.

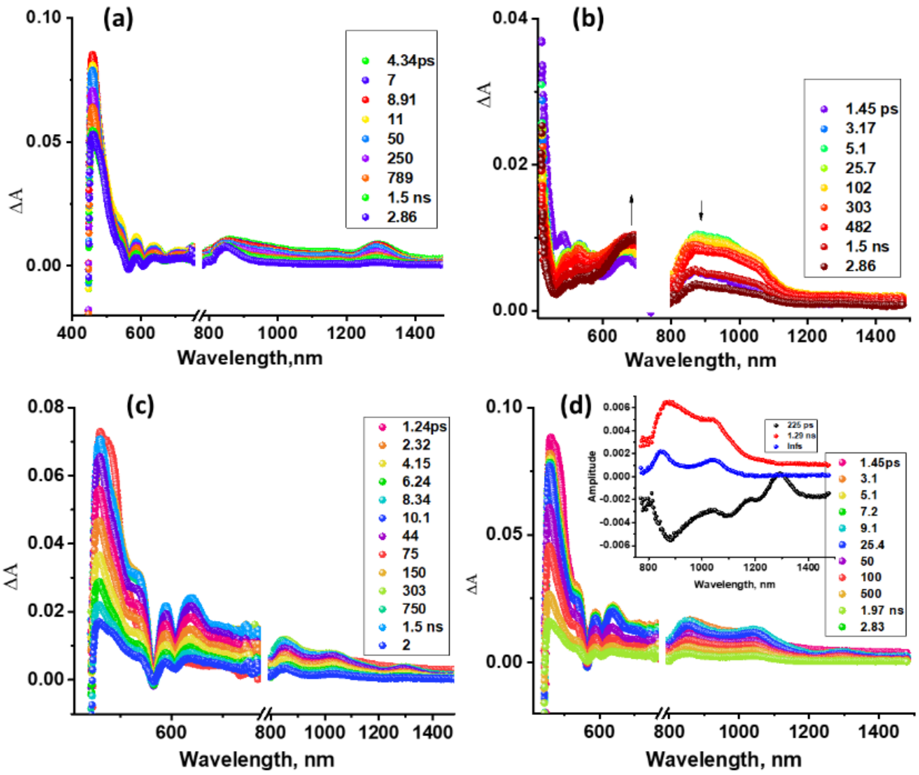

Figure S46. Femtosecond transient-absorption (fs-TA) spectra at the indicated delay times of (a) ZnP-COOH (**7**), (b) compound **6**, (c) compound **1**, and (d) compound **2**. All spectra were recorded in deaerated benzonitrile. Porphyrin-containing compounds were excited at 435 nm, whereas compound **6** was excited at 363 nm. Decay-associated spectra in the near-IR region for **2** are shown in Figure d inset.

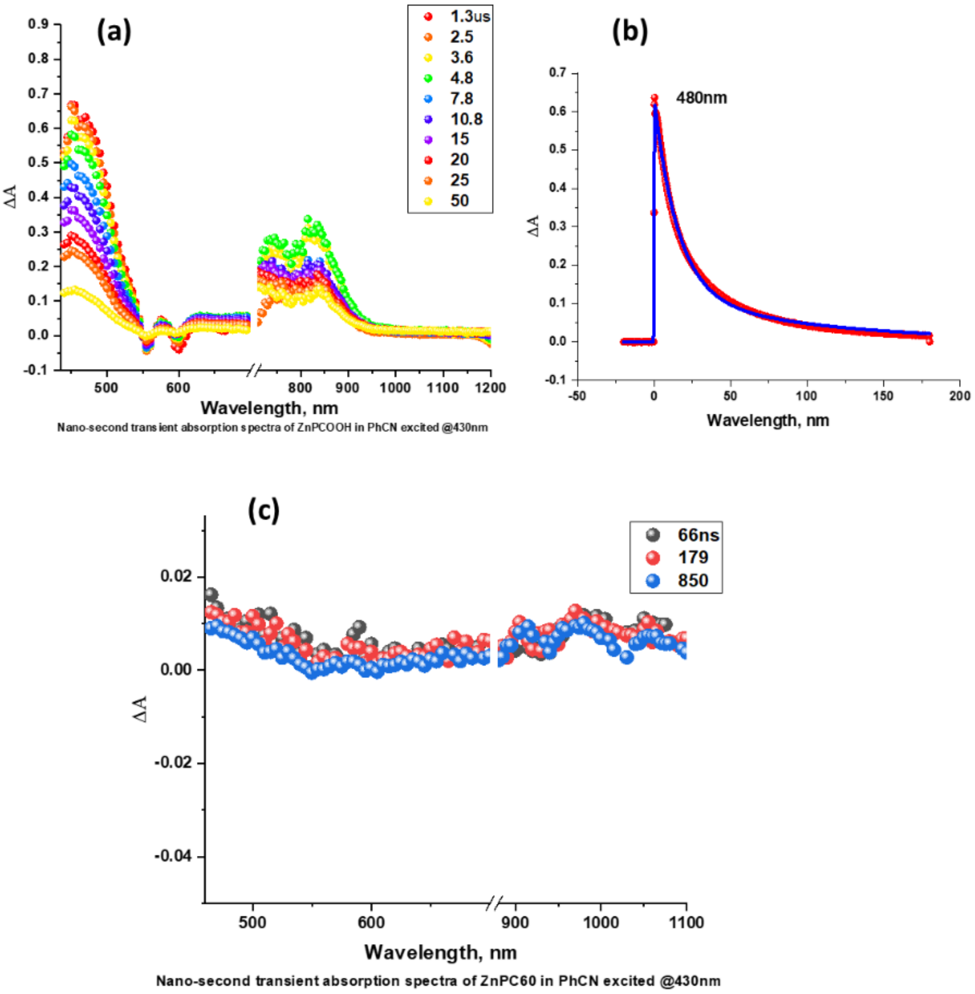

Figure S47. Nanosecond transient-absorption (ns-TA) spectra of (a) ZnP-COOH (**7**) and (c) compound **2** in benzonitrile. Both compounds were excited at 430 nm. The time profile of the 480 nm peak of ^{3}ZnP* is shown in figure b.

References

[1] Horcas, I.; Fernandez, R.; Gomez-Rodriguez, J.M.; Colchero, J.; Gomez-Herrero J.; Baro, A. M. *Rev. Sci. Instrum.* **2007**, 78, 13705.

[2] Hao, X.Q.; Wang, Y.N.; Liu, J.R.; Wang, K.L.; Gong, J.F.; Song, M.P. *J. Organomet. Chem.* **2010,** *695*, 82

[3] Prato, M.; Soombar, C.; Vazquez, E.; Niziol, J.; Gondek, E.; Rau, I.; Kajzar, F. *Molecular Crystals and Liquid Crystals*, **2010,** *521*, 253.

[4] Spicher, S.; Grimme, S. *Angew. Chemie Int. Ed.* **2020**, *59*, 15665–15673.

[5] Bannwarth, C.; Ehlert, S.; Grimme, S. *J. Chem. Theory Comput.* **2019**, *15*, 1652–1671.

[6] Yang, Y.; Yu, H.; York, D.; Cui Q.; Elstner, M. *J. Phys. Chem. A.* **2007**, *111*, 10861–10873. Gaus, M.; Goez, A. Elstner, M. *J. Chem. Theory Comput.* **2013**, *9*, 338‑354.

[7] a) Becke, A. D. *Phys. Rev. A* **1988**, *38*, 3098–3100. b) Lee, C.; Yang, W.; Parr, R. G. *Phys. Rev. B* **1988**, *37*, 785–789. c) Francl, M. M.; Pietro, W. J.; Hehre, W. J.; Binkley, J. S.; Gordon, M. S.; DeFrees, D. J.; Pople, J. A. *J. Chem. Phys.* **1982**, *77*, 3654–3665.

[8] Frisch, M. J.; Trucks, G. W.; Schlegel, H. B.; Scuseria, G. E.; Robb, M. A.; Cheeseman, J. R.; Scalmani, G.; Barone, V.; Petersson, G. A.; Nakatsuji, H.; Li, X.; Caricato, M.; Marenich, A. V.; Bloino, J.; Janesko, B. G.; Gomperts, R.; Mennucci, B.; Hratchian, H. P.; Ortiz, J. V.; Izmalov, A. F.; Sonnenberg, J. L.; Williams-Young, D.; Ding, F.; Lipparini, F.; Egidi, F.; Goings, J.; Peng, B.; Petrone, A.; Henderson, T.; Ranasinghe, D.; Zakrzewski, V. G.; Gao, J.; Rega, N.; Zheng, G.; Liang, W.; Hada, M.; Ehara, M.; Toyota, K.; Fukuda, R.; Hasegawa, J.; Ishida, M.; Nakajima, T.; Honda, Y.; Kitao, O.; Nakai, H.; Vreven, T.; Throsell, K.; Montgomery, J. A.; Jr.; Peralta, J. E.; Ogliaro, F.; Beapark, M. J.; Heyd, J. J.; Brothers, E. N.; Kudin, K. N.; Staroverov, V. N.; Keith, T. A.; Kobayash, R.; Normand, J.; Raghavachari, K.; Rendell, A. P.; Burant, J. C.; Iyengar, S. S.; Tomasi, J.; Cossi, M.; Millam, J. M.; Klene, M.; Adamo, C.; Cammi, R.; Ochterski, J. W.; Martin, R. L.; Morokuma, K.; Farkas, O.; Foresman, J. B.; Fox, D. J. Gaussian Inc.: Wallingford CT 2016.

[9] Risthaus, T.; Grimme, S. *J. Chem. Theory Comput.* **2013**, *9*, 1580–1591.

[10] Scalmani, G.; Frisch, M. J. *J. Chem. Phys.* **2010**, *132*, 114110.
